# Military service, deployments, and exposures in relation to amyotrophic lateral sclerosis survival

**DOI:** 10.1371/journal.pone.0185751

**Published:** 2017-10-10

**Authors:** John D. Beard, Lawrence S. Engel, David B. Richardson, Marilie D. Gammon, Coleen Baird, David M. Umbach, Kelli D. Allen, Catherine L. Stanwyck, Jean Keller, Dale P. Sandler, Silke Schmidt, Freya Kamel

**Affiliations:** 1 Department of Epidemiology, Gillings School of Global Public Health, University of North Carolina at Chapel Hill, Chapel Hill, North Carolina, United States of America; 2 Epidemiology Branch, National Institute of Environmental Health Sciences, Research Triangle Park, North Carolina, United States of America; 3 Environmental Medicine Program, US Army Public Health Command, Aberdeen Proving Ground, Maryland, United States of America; 4 Biostatistics and Computational Biology Branch, National Institute of Environmental Health Sciences, Research Triangle Park, North Carolina, United States of America; 5 Durham VA Medical Center, Durham, North Carolina, United States of America; 6 Department of Medicine and Thurston Arthritis Research Center, University of North Carolina at Chapel Hill, Chapel Hill, North Carolina, United States of America; 7 Department of Medicine, Duke University Medical Center, Durham, North Carolina, United States of America; 8 Westat, Inc., Durham, North Carolina, United States of America; Institute for Clinical Epidemiology and Applied Biometry, GERMANY

## Abstract

**Background:**

Military veterans may have higher rates of amyotrophic lateral sclerosis (ALS) mortality than non-veterans. Few studies, with sparse exposure information and mixed results, have studied relationships between military-related factors and ALS survival. We evaluated associations between military-related factors and ALS survival among U.S. military veteran cases.

**Methods:**

We followed 616 medical record-confirmed cases from enrollment (2005–2010) in the Genes and Environmental Exposures in Veterans with Amyotrophic Lateral Sclerosis study until death or July 25, 2013, whichever came first. We ascertained vital status information from several sources within the Department of Veterans Affairs. We obtained information regarding military service, deployments, and 39 related exposures via standardized telephone interviews. We used Cox proportional hazards regression models to estimate hazard ratios (HRs) and 95% confidence intervals. We adjusted for potential confounding and missing covariate data biases via inverse probability weights. We also used inverse probability weights to adjust for potential selection bias among a case group that included a disproportionate number of long-term survivors at enrollment.

**Results:**

We observed 446 deaths during 24,267 person-months of follow-up (median follow-up: 28 months). Survival was shorter for cases who served before 1950, were deployed to World War II, or mixed and applied burning agents, with HRs between 1.58 and 2.57. Longer survival was associated with exposure to: paint, solvents, or petrochemical substances; local food not provided by the Armed Forces; or burning agents or Agent Orange in the field with HRs between 0.56 and 0.73.

**Conclusions:**

Although most military-related factors were not associated with survival, associations we observed with shorter survival are potentially important because of the large number of military veterans.

## Introduction

Amyotrophic lateral sclerosis (ALS) is a debilitating neurodegenerative disease of motor neurons in the central nervous system [1]. Median survival after diagnosis is three years, but 4% of cases survive past 10 years [1]. Factors consistently associated with rapid progression or shorter survival include older diagnosis age [2–5], bulbar onset [2–6], clinical features (e.g., worse functional/disability status) [2, 3], shorter diagnostic delay [2, 5, 6], and definite diagnosis category [2]. Few studies, however, have considered non-clinical (e.g., occupational or environmental) prognostic factors for ALS survival, but these factors could help explain the extremely heterogeneous survival of ALS patients. In addition, identifying associations between non-clinical factors and ALS survival could help clinicians target interventions that may have the potential to lengthen survival, such as interdisciplinary palliative care [2], to patients at greatest risk of shorter survival.

Limited evidence suggests ALS rates may be higher among military veterans, particularly those deployed to the 1990–1991 Persian Gulf War (hereafter “Gulf War”) [7–10]. The few studies that examined associations between military-related factors and ALS survival or progression [5, 6, 11, 12] conflict and contain sparse exposure information. A study of the ALS Clinical Assessment, Research and Education database, a non-military database of North American patients [13], found no association between veteran status and survival [11], whereas a study in Massachusetts reported being a male veteran was associated with faster progression after one year of follow-up [6]. Deployment to the Gulf War was associated with shorter survival in one study [12], but not another [5]. The latter, based on the U.S. National Registry of Veterans with ALS (hereafter “Registry”) [14], also found shorter survival was associated with deployment to Vietnam, but not with cumulative military service, service branch, or deployment to Korea [5]. We evaluated associations between ALS survival and aspects of military service, deployments, and 39 related exposures using additional data on a subset of Registry cases enrolled in the Genes and Environmental Exposures in Veterans with Amyotrophic Lateral Sclerosis study (GENEVA) [15].

## Materials and methods

### Study population and outcome assessment

GENEVA is a case-control study of veterans, conducted from 2005–2010, that includes ALS cases from the Registry [14] and controls identified via U.S. Department of Veterans Affairs (VA) databases [15]. Although participants did not have to have been deployed to war to enroll in GENEVA, they did have to have been members of the U.S. Army, Air Force, Navy, Marine Corps, Coast Guard, activated Reserves or National Guard at some point in time [16, 17]. We previously used data on GENEVA cases and controls to evaluate associations between military-related factors and ALS etiology [17]. The present analysis focused on ALS survival and included cases only.

Methods used to enroll cases first in the Registry, then in GENEVA, were published previously [14, 15]. Briefly, the VA started the Registry because of increased ALS rates among veterans who had been deployed to the Gulf War [14, 15, 18]. Recruitment of potential cases occurred from 2003 to 2007 (i.e., the year the original Gulf War ALS studies [9, 10] were published to when funding expired) and included searches of national VA databases for patients with *International Classification of Diseases*, *9th Revision* [19], codes of the form 335.2X (motor neuron diseases) and publicizing the Registry [14]. In total, there were 7,116 potential cases identified, 4,626 who completed a telephone screening [16], and 2,600 who reported a past diagnosis [14]. Of these, there were 2,400 who consented to join, 2,265 who allowed their medical records to be reviewed by ALS specialist neurologists, and 2,122 who had a diagnosis of ALS or another motor neuron disease confirmed using the Revised El Escorial Criteria [14, 20]. These 2,122 cases were followed until 2009 via standardized semi-annual telephone interviews [17].

**Fig 1:**
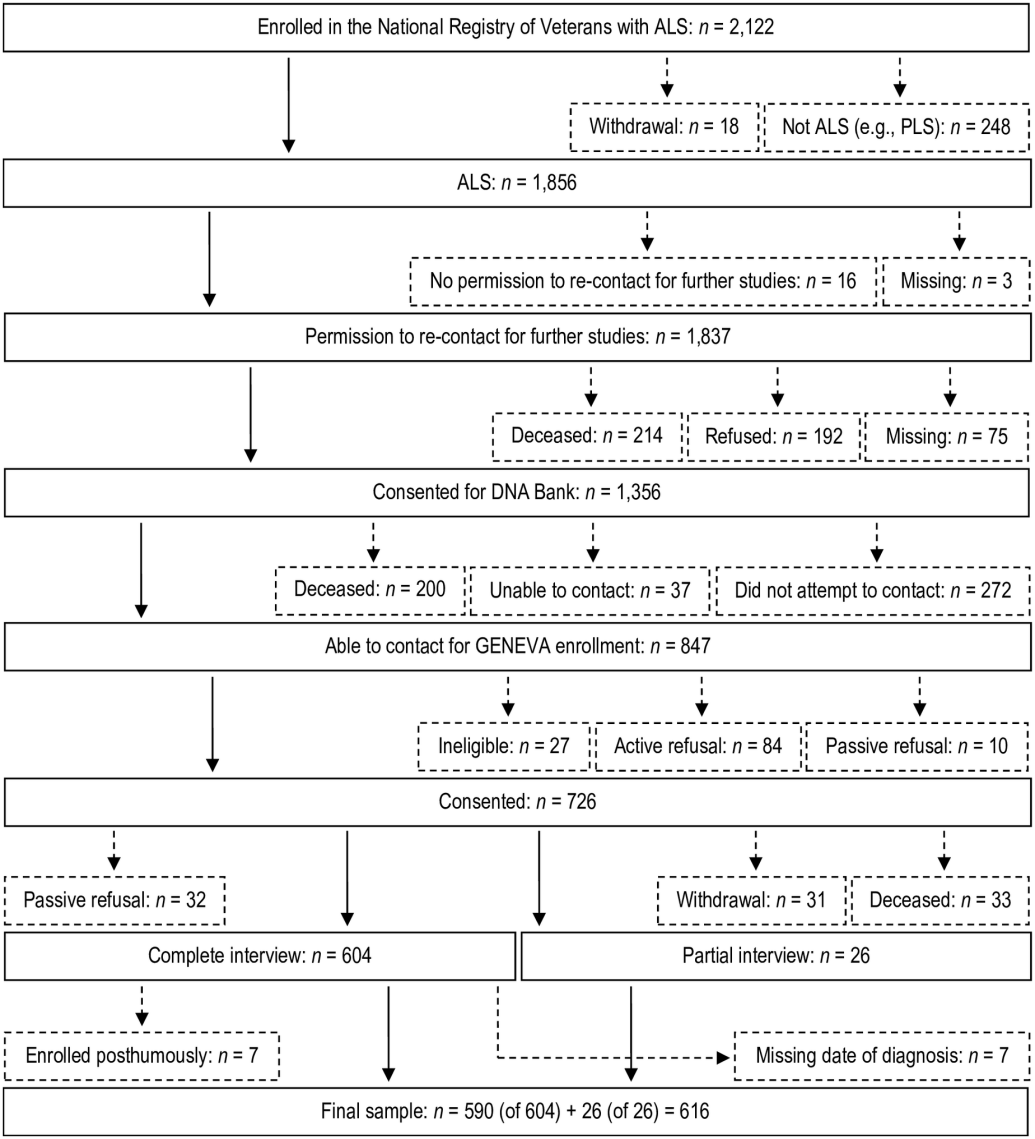
Flow chart showing the ascertainment of GENEVA cases, United States of America, 2005–2010. Solid boxes or lines depict cases who progressed past each step shown. Small-dashed boxes or lines depict cases who did not progress past each step shown, but who were incorporated into the analysis indirectly via inverse probability weights (see Statistical Analyses and Section C in S1 File for more details). Among the 27 cases ineligible for GENEVA, reasons included dementia (n = 9), diagnosis change (n = 4), withdrawal from the Registry DNA bank (n = 3), or interview infeasibility (n = 11). Abbreviations: ALS, amyotrophic lateral sclerosis; GENEVA, Genes and Environmental Exposures in Veterans with Amyotrophic Lateral Sclerosis study; PLS, primary lateral sclerosis.

The ascertainment of GENEVA cases from the Registry is shown in Fig 1. Of 1,856 ALS cases, there were 1,837 who gave permission to be re-contacted for further studies, 1,356 who consented to join the Registry DNA bank, and 847 who were able to be contacted regarding GENEVA enrollment. Of these, there were 726 who consented to join and 630 who enrolled, of which we included 616 in this analysis because they had information on diagnosis date and were not enrolled posthumously by proxy. These cases were comprised of patients with clinically definite, probable, possible, or suspected ALS.

The outcome we used was days from diagnosis date to death date or end of follow-up (July 25, 2013), with late entry at GENEVA enrollment date. Time since diagnosis was the most clinically meaningful time scale and allowing for late entry at enrollment date addressed potential immortal person-time bias, which occurs when follow-up time that occurs before the last event required to enter the study is counted as person-time at risk for death [21], from left truncation. Time to death is recommended over other ALS survival outcomes (e.g., time to use of ventilator or death), which are not uniformly defined or used across centers [22].

We extracted information on diagnosis date from medical records [16]. We obtained vital status information from the Austin Vital Status File, which contains information from several sources within the VA [23]. Using the National Death Index [24] as the gold standard, a validation study found the Austin Vital Status File was 98.3% sensitive and 99.8% specific (κ = 0.981) for identifying deaths among veterans [23]. Thus, it is likely we missed only a small proportion of deaths among GENEVA cases.

The Institutional Review Boards (IRBs) of the Durham VA Medical Center, Duke University Medical Center, and the National Institute of Environmental Health Sciences approved GENEVA; this ancillary study was approved by the IRB of the University of North Carolina at Chapel Hill. All participants provided written or oral informed consent before enrollment, which we tracked via Microsoft Access databases and documented by maintaining copies of signed informed consent forms. We also mailed copies of informed consent forms to each participant. For the Registry, oral informed consent was sought and approved by the IRB of the Durham VA Medical Center when participants were unable to provide written informed consent due to the progression of their disease. For GENEVA, oral informed consent was sought and approved by the IRBs of the Durham VA Medical Center and Duke University Medical Center for all ALS cases because the standardized telephone interview was deemed to involve no more than minimal risk of harm to the participants.

### Exposure assessment

We used medical records as the source of information on clinical features and the Registry semi-annual telephone interviews [16] as the source of baseline (closest to GENEVA enrollment) ALS Functional Rating Scale-Revised (ALSFRS-R) score [17, 25]. Standardized telephone interviews for GENEVA [16] provided information on potential confounders and military service, deployments, and exposures that occurred before diagnosis date [15]. We had to conduct interviews via proxy for 27 (4%) cases. Interview procedures have been described in more detail elsewhere [15].

We collected information on military service—including longest service branch (i.e., the branch in which the veteran served the longest total time), number of branches, rank, total service time, and end of most recent service (Section A in S1 File)—and on deployments to World War II (WWII) and the Korean, Vietnam, and Gulf Wars (hereafter “four wars”) and operations in nine other locations—including ever deployment to any war/operation, war/operation of longest deployment (i.e., the war or operation to which the veteran was deployed for the longest total time), total time of all deployments, and end of most recent deployment [17] (Section A in S1 File). We also recorded information on ever deployment to any other country; ever receiving imminent danger pay, hardship duty, or combat zone tax exclusion benefits for deployment to 17 foreign countries and five sea regions (plus fill-in options); and total time deployed to those countries/sea regions [17] (Section A in S1 File).

To identify specific military exposures, we adapted questionnaires used in the Iowa Gulf War study [26] and the Millennium Cohort Study [27, 28] for use in GENEVA [15, 16]. We also added a few questions on exposures that were specific to one or more of the four wars (e.g., Agent Orange exposure during the Vietnam War) [17]. In total, we collected information on 39 specific military exposures, 32 of which we queried only in reference to exposure during deployment to the four wars [17], which included 16–228 cases depending on the war(s). For the seven non-war-specific exposures, we asked cases about ever exposure and, for some exposures, a quantification of exposure (e.g., number of pills) [17]. For 31 of 32 war-specific exposures, we collected information on whether cases had ever been exposed, days exposed (Not exposed, ≤ 5, 6–30, > 30), and whether they felt ill after exposure (Not exposed, No, Yes) [17]. For the other war-specific exposure, total vaccinations, we asked cases about number of shots received [17].

### Statistical analyses

We used Cox proportional hazards regression models with time since diagnosis as the time scale to calculate hazard ratios (HRs) and 95% confidence intervals (CIs) for associations between military-related factors and ALS survival. We checked the proportional hazards assumption by evaluating the statistical significance of interactions between each covariate and the natural logarithm of time since diagnosis [29].

We identified prognostic factors for ALS survival from previous literature to consider as potential confounders and used directed acyclic graphs [30] to identify minimally sufficient adjustment sets among these factors (Figure A and Section B in S1 File). For military service and deployment factors the minimally sufficient adjustment set was age and sex and for military exposures the minimally sufficient adjustment set was age, sex, and war/operation of deployment. We did not include sex in our models due to small numbers of female cases (n = 13) and deaths (n = 10).

We calculated three types of stabilized inverse probability weights (hereafter “weights”) [31, 32], a type of propensity score, to adjust for potential bias from (1) confounding by age and war/operation of deployment (for military exposures), (2) missing data on ALSFRS-R score for 8% of Registry cases, and (3) selection arising from a case group that included a disproportionate number of long-term survivors at GENEVA enrollment [15] (Section C and Table A in S1 File). Although we calculated confounding weights for each exposure separately, we used the same missing-covariate-data and selection weights for every exposure. We assessed appropriateness of the weights using established criteria [31] (Section C in S1 File). We calculated overall stabilized weights by multiplying the three types of weights together [31, 33]. We then applied the overall stabilized weight to Cox proportional hazards regression models that contained the exposure of interest as the only explanatory variable (sampling weights are applied in the same way when analyzing data from complex survey sampling designs) [31, 33]. We used robust variance estimates to calculate 95% CIs for the HRs because using weights for analysis induces within-subject correlation [34].

Using weights for analysis creates a pseudo-population in which there is no bias from confounding, missing covariate data, or selection due to measured variables that are incorporated into the calculation of the weights [31, 32]. The weighted HRs and 95% CIs we calculated thus generalize to the 1,786 Registry cases who: had diagnoses of clinically definite, probable, possible, or suspected ALS; were not enrolled in the Registry posthumously by proxy; had diagnosis date available; and had no missing data on any covariates used to calculate the weights.

We used within-category medians as scores to assess linear exposure-response trends for categorized versions of continuous variables [35] and scaled corresponding HRs to one unit or interquartile range (IQR) increases in the original continuous variables. To assess linear trends for days exposed to each of 31 exposures experienced during deployment to the four wars, we used category midpoints, and 50% above the lower bound of the top category, as scores and scaled corresponding HRs to 20-day increases. We assessed linear trends for feeling ill after exposure to each of the same 31 exposures by using ordinal scores.

Our study is largely hypothesis-generating because most of the military-related factors we studied have not been evaluated in relation to ALS survival by previous studies. Therefore, we used the magnitude of associations (e.g., HRs greater than 1.25), the number of exposed ALS deaths (e.g., five or more), and, where appropriate, evidence of exposure-response trends rather than statistical significance to guide our choice of results to highlight in the text. However, we presented results for all military-related factors in the tables and S1 File for transparency and to stimulate further research.

Following the advice of several epidemiologists (e.g., [36–41]), we did not use conventional multiple comparisons adjustments, such as false discovery rate [42], because our study is not based on a random sample, does not include random exposure assignment, and assignment of exposures cannot be thought of as approximately random [43]. In addition, hierarchical regression models [44] were impractical for our study because of differing sample sizes available for the exposures and because it is not clear (1) how one would group the exposures, (2) whether the assumptions made to group the exposures would be valid [45], and (3) whether the groups would be meaningful chemically or biologically.

We performed several secondary analyses including separately analyzing war-specific exposures for veterans who were deployed to the Vietnam War (64% of those with war-specific data); the small number of veterans deployed to other wars precluded performing similar analyses for them. To reduce sample heterogeneity, we separately restricted to cases who: enrolled in GENEVA within two years of diagnosis (39%); had clinically definite or probable ALS (75%); or never visited or resided in western Pacific regions with high endemic ALS [1] (84%). As an alternative way to control for confounding by age, we used age as the timescale, with late entry at GENEVA enrollment date. To control confounding by sex, we restricted to men (98%). We restricted to non-proxy interviews (96%) to mitigate potential bias from exposure misclassification. In addition, we recoded implausible values to missing for four exposures (anthrax vaccination, smallpox vaccination, nasopharyngeal radium, pyridostigmine bromide pills) that had distinct time periods of use. We identified correlations among the 39 military exposures by calculating Spearman correlation coefficients and then mutually-adjusted exposures that were more than weakly correlated (absolute value of coefficients > 0.2). Finally, we adjusted for potential confounding via standard regression methods, but did not weight for potential missing-covariate-data or selection bias. We conducted all analyses using SAS version 9.3 (SAS Institute Inc., Cary, NC, 2011).

## Results

At the end of follow-up, 24,267 person-months had been observed since enrollment in GENEVA (Table 1). Median (IQR) follow-up was 28 (59) months and 446 (72%) cases had died (median [IQR] follow-up among the deceased was 17 [27] months). HRs increased with increasing age (Table 1). No other demographic characteristic was associated with survival.

**Table 1:** Demographic characteristics of amyotrophic lateral sclerosis deaths and total cases^a^ in GENEVA, United States of America, 2005–2013.

Characteristic	Deaths		Total		Crude		Adjusted^b^	
	No.	PM^c^	No.	PM^c^	HR^d^	95% CI^d^	HR^d^	95% CI^d^
Total	446	10,558	616	24,267				
Age (years)								
≤ 39	17	409	42	2,470	0.60	0.36, 1.02		
40–49	35	1,150	68	3,853	0.74	0.50, 1.09		
50–59	117	2,893	171	7,186	1.00	Referent		
60–69	165	3,624	203	6,708	1.45	1.14, 1.84		
70–79	100	2,264	119	3,767	1.45	1.11, 1.90		
> 79	12	219	13	283	1.98	1.09, 3.59		
Median (IQR)	62 (14)		60 (14)		1.43^e^	1.26, 1.63^e^		
Sex								
Male	436	10,240	603	23,698	1.00	Referent	1.00	Referent
Female	10	318	13	569	1.00	0.53, 1.87	1.26	0.66, 2.39
Race/Ethnicity								
Non-Hispanic White	413	9,491	561	21,419	1.00	Referent	1.00	Referent
Other	29	1,009	48	2,569	0.68	0.47, 1.00	0.74	0.51, 1.09
Missing	4		7					
Use of VA health care system								
No	254	5,920	361	14,502	1.00	Referent	1.00	Referent
Yes	192	4,639	254	9,686	1.04	0.86, 1.25	1.00	0.82, 1.21
Missing	0		1					
Ever applied for VA benefits of any type								
No	73	1,536	100	3,741	1.00	Referent	1.00	Referent
Yes	335	7,918	455	17,264	0.90	0.70, 1.16	0.92	0.71, 1.19
Missing	38		61					
Highest degree attained								
Grade school (grades 1–8)	17	496	20	753	1.22	0.74, 2.00	1.00	0.61, 1.66
High school diploma or other	191	4,455	266	10,474	1.00	Referent	1.00	Referent
Technical or vocation school diploma or Associate degree	81	1,604	114	4,207	0.95	0.73, 1.24	0.96	0.73, 1.25
College or graduate school diploma	145	3,692	199	8,152	0.98	0.79, 1.22	0.99	0.80, 1.24
Doctorate	12	310	17	681	0.88	0.49, 1.59	0.84	0.47, 1.52
Cigarette smoking status								
Never	141	3,261	207	8,558	1.00	Referent	1.00	Referent
Past	232	5,531	305	11,380	1.13	0.92, 1.40	1.04	0.84, 1.29
Current	58	1,547	85	3,808	0.96	0.71, 1.31	1.02	0.75, 1.39
Missing	15		19					

Abbreviations: ALS, amyotrophic lateral sclerosis; CI, confidence interval; GENEVA, Genes and Environmental Exposures in Veterans with Amyotrophic Lateral Sclerosis study; HR, hazard ratio; IQR, interquartile range; PM, person-months; VA, Department of Veterans Affairs.

^a^ Fourteen ALS cases were excluded from this analysis because (1) they were missing data on diagnosis date (n = 7) or (2) they died before GENEVA enrollment (i.e., enrollment was completed by proxy after the case died; n = 7).

^b^ Adjusted for age (modeled with indicator variables corresponding to 5-year groups).

^c^ Person-months calculated for time on study (i.e., the difference between the GENEVA enrollment date and the death date or July 25, 2013).

^d^ HRs and 95% CIs correspond to time since diagnosis accounting for late entry into the risk set at the GENEVA enrollment date.

^e^ Scaled the HR to an IQR-unit increase in age among all cases.

After adjustment for age, HRs were higher among cases who had bulbar onset, but lower among those with longer diagnostic delays or higher baseline ALSFRS-R scores (Table 2). HRs decreased with increases in time from diagnosis to Registry enrollment, but increased with increases in time from Registry enrollment to GENEVA enrollment. Treatments associated with poor condition at baseline (e.g., use of ventilator) had elevated HRs.

**Table 2:** Clinical characteristics of amyotrophic lateral sclerosis deaths and total cases^a^ in GENEVA, United States of America, 2005–2013.

Characteristic	Deaths		Total		Crude		Adjusted^b^	
	No.	PM^c^	No.	PM^c^	HR^d^	95% CI^d^	HR^d^	95% CI^d^
Most recent ALS diagnosis category								
Clinically definite	99	2,671	148	6,778	0.99	0.77, 1.26	1.04	0.81, 1.33
Clinically probable	232	5,140	311	11,522	1.00	Referent	1.00	Referent
Clinically possible	37	854	47	1,681	1.18	0.83, 1.67	1.22	0.86, 1.74
Suspected (progressive bulbar palsy or progressive muscular atrophy)	78	1,894	110	4,286	1.04	0.80, 1.35	0.98	0.76, 1.28
Symptom onset site								
Bulbar	70	1,362	85	2,551	1.35	1.04, 1.75	1.23	0.94, 1.61
Extremities	352	8,586	499	20,512	1.00	Referent	1.00	Referent
Other^e^	23	579	31	1,171	0.84	0.55, 1.29	0.96	0.63, 1.48
Missing	1		1					
Time from symptom onset to diagnosis (months)								
≤ 6	90	2,299	116	4,483	0.85	0.65, 1.11	0.88	0.67, 1.15
> 6–12	138	2,868	166	5,041	1.00	Referent	1.00	Referent
> 12–18	73	1,434	104	3,869	0.74	0.56, 0.98	0.77	0.58, 1.03
> 18–24	36	799	46	1,617	0.88	0.61, 1.28	0.85	0.59, 1.24
> 24	104	2,940	172	8,426	0.56	0.43, 0.72	0.49	0.38, 0.64
Missing	5		12					
Median (IQR)	11 (15)		13 (18)		0.82^f^	0.75, 0.91^f^	0.77^f^	0.70, 0.86^f^
Time from diagnosis to enrollment in the Registry (months)								
≤ 12	185	3,372	216	5,699	1.00	Referent	1.00	Referent
> 12–24	116	2,838	142	4,865	0.95	0.74, 1.21	0.86	0.67, 1.11
> 24–36	47	1,091	75	3,347	0.76	0.53, 1.09	0.73	0.50, 1.05
> 36–48	26	643	44	2,071	0.83	0.51, 1.32	0.76	0.47, 1.23
> 48	72	2,614	139	8,284	0.73	0.44, 1.21	0.70	0.42, 1.17
Median (IQR)	15 (25)		19 (32)		0.82^f^	0.66, 1.03^f^	0.82^f^	0.65, 1.02^f^
Time from enrollment in the Registry to enrollment in the GENEVA study (months)								
≤ 6	140	2,795	178	5,634	0.99	0.78, 1.26	1.01	0.79, 1.30
> 6–12	142	3,101	204	8,157	1.00	Referent	1.00	Referent
> 12–18	72	1,951	95	3,896	1.36	1.02, 1.81	1.30	0.97, 1.74
> 18–24	51	1,526	71	3,286	1.34	0.96, 1.86	1.36	0.97, 1.90
> 24	41	1,186	68	3,294	1.31	0.90, 1.90	1.34	0.92, 1.95
Median (IQR)	9 (11)		9 (11)		1.16^f^	1.01, 1.34^f^	1.18^f^	1.02, 1.37^f^
Baseline^g^ ALSFRS-R score (possible range: 0–48)^h^								
≤ 16	108	2,071	133	4,167	1.87	1.41, 2.47	2.09	1.57, 2.77
> 16–23	94	1,815	116	3,674	1.50	1.13, 1.99	1.69	1.27, 2.25
> 23–30	99	2,730	137	5,787	1.00	Referent	1.00	Referent
> 30–35	77	1,792	111	4,466	0.88	0.65, 1.19	0.89	0.66, 1.21
> 35	68	2,151	119	6,172	0.53	0.39, 0.73	0.56	0.41, 0.76
Median (IQR)	26 (16)		27 (16)		0.54^f^	0.47, 0.62^f^	0.51^f^	0.44, 0.59^f^
Currently using riluzole (baseline^g^)								
No	207	5,107	298	12,448	1.00	Referent	1.00	Referent
Yes	229	5,200	302	11,166	1.08	0.90, 1.31	1.14	0.94, 1.39
Missing	10		16					

Abbreviations: ALS, amyotrophic lateral sclerosis; ALSFRS-R, ALS Functional Rating Scale-Revised; CI, confidence interval; GENEVA, Genes and Environmental Exposures in Veterans with Amyotrophic Lateral Sclerosis study; HR, hazard ratio; IQR, interquartile range; PM, person-months.

^a^ Fourteen ALS cases were excluded from this analysis because (1) they were missing data on diagnosis date (n = 7) or (2) they died before GENEVA enrollment (i.e., enrollment was completed by proxy after the case died; n = 7).

^b^ Adjusted for age (modeled with indicator variables corresponding to 5-year groups).

^c^ Person-months calculated for time on study (i.e., the difference between the GENEVA enrollment date and the death date or July 25, 2013).

^d^ HRs and 95% CIs correspond to time since diagnosis accounting for late entry into the risk set at the GENEVA enrollment date.

^e^ Includes “all over” (deaths: n < 5; total: n < 5), “cramps/fasciculations” (deaths: n = 18; total: n = 26), and “loss of appetite” (deaths: n < 5; total: n < 5). Any numbers presented as “n < 5” were suppressed to preserve the confidentiality of study participants.

^f^ Scaled the HR and 95% CI to an IQR-unit increase in time from symptom onset to diagnosis, time from diagnosis to enrollment in the Registry, time from enrollment in the Registry to enrollment in GENEVA, or baseline ALSFRS-R score among all cases.

^g^ The ALSFRS-R score or use status of riluzole that was measured closest to the time of GENEVA enrollment (i.e., interview).

^h^ Category boundaries were set at quintiles of the ALSFRS-R score among all cases.

In weighted analyses, HRs were elevated among cases who served ≤ 1 year in the military or before July 1950 (Table 3). Cases who were *deployed* to WWII or before July 1950 had higher HRs than those who were not deployed (Table 4). There was a significant inverse trend between end of most recent deployment and mortality (HR = 0.65; 95% CI: 0.48, 0.89), although category-specific associations were not monotonic.

**Table 3:** Military service and amyotrophic lateral sclerosis survival in GENEVA^a^, United States of America, 2005–2013.

Variable	Deaths		Total		IP-weighted^b^	
	No.	PM^c^	No.	PM^c^	HR^d^	95% CI^d^
Military branch of longest service						
Air Force (including Army Air Force)	97	2,124	129	4,784	1.09	0.83, 1.44
Army	182	4,403	249	9,775	1.00	Referent
Marines (including Merchant Marines)	36	638	55	2,175	0.85	0.50, 1.43
Navy	98	2,570	136	5,616	0.87	0.65, 1.16
Other^e^	32	816	45	1,815	0.76	0.51, 1.13
Missing	1		2			
Number of military branches of service						
1 (Median = 1)	411	9,774	565	22,300	1.00	Referent
> 1 (2)	35	785	51	1,967	1.01	0.75, 1.38
Officer or Warrant Officer						
No	373	8,685	514	20,110	1.00	Referent
Yes	72	1,858	100	4,060	0.96	0.72, 1.30
Missing	1		2			
Years of military service						
≤ 1 (0.50)	13	241	15	400	1.80	1.11, 2.90
> 1–5 (2.92)	305	7,022	414	15,896	1.00	Referent
> 5–10 (7.92)	42	1,202	59	2,497	0.88	0.59, 1.31
> 10–15 (11.99)	14	436	26	1,398	0.80	0.48, 1.33
> 15–20 (18.92)	19	502	21	684	0.85	0.64, 1.11
> 20 (24.31)	52	1,147	79	3,290	1.02	0.71, 1.47
Missing	1		2			
Trend (IQR = 6.00)^f^^,^ ^g^					1.00	0.91, 1.10
End of most recent period of military service (month/year)^h^						
≤ 12/1946 (03/1946)	26	593	32	1,073	1.58	0.96, 2.61
01/1947-06/1950 (02/1948)	9	185	11	341	2.23	1.06, 4.70
07/1950-01/1955 (06/1953)	48	1,332	56	2,003	1.04	0.71, 1.52
02/1955-02/1961 (12/1957)	78	1,648	97	3,266	1.18	0.83, 1.70
03/1961-07/1964 (06/1963)	34	849	43	1,603	1.04	0.66, 1.64
08/1964-04/1975 (07/1969)	134	3,160	192	7,732	1.00	Referent
05/1975-08/1980 (05/1978)	26	606	42	1,903	0.92	0.54, 1.56
09/1980-07/1990 (03/1986)	36	818	53	2,156	0.84	0.59, 1.21
08/1990-08/2001 (06/1994)	44	1,177	71	3,299	0.99	0.65, 1.49
> 08/2001 (10/2003)	10	182	17	789	0.94	0.48, 1.83
Missing	1		2			
Trend (20 years, 11.99 months)^f^^,^ ^g^					0.98	0.81, 1.18

Abbreviation: ALS, amyotrophic lateral sclerosis; ALSFRS-R, ALS Functional Rating Scale-Revised; CI, confidence interval; GENEVA, Genes and Environmental Exposures in Veterans with Amyotrophic Lateral Sclerosis study; HR, hazard ratio; IP, inverse probability; IQR, interquartile range; PM, person-months; VA, Department of Veterans Affairs.

^a^ Fourteen ALS cases were excluded from this analysis because (1) they were missing data on diagnosis date (n = 7) or (2) they died before GENEVA enrollment (i.e., enrollment was completed by proxy after the case died; n = 7).

^b^ Weighted for confounding (conditional on age [modeled with indicator variables corresponding to 5-year groups]), not missing baseline ALSFRS-R score (conditional on most recent ALS diagnosis category, symptom onset site, diagnostic delay [months; modeled with the natural logarithm of a linear term], and time from diagnosis to enrollment in the Registry [months; modeled with a restricted, quadratic spline with knots at 7.72, 13.24, 23.06, and 44.19 months based on percentiles of the distribution in the Registry cases not missing baseline ALSFRS-R score]), and participating in GENEVA (conditional on race/ethnicity, being a current patient of a VA Medical Center, most recent ALS diagnosis category, symptom onset site, diagnostic delay [months; modeled with linear, quadratic, and cubic terms], time from diagnosis to enrollment in the Registry [months; modeled with a linear term], and baseline ALSFRS-R score [modeled with a restricted, quadratic spline with knots at 12, 34, and 44 based on percentiles of the distribution in GENEVA cases]). 95% CIs were calculated with robust variance estimates.

^c^ Person-months calculated for time on study (i.e., the difference between the GENEVA enrollment date and the death date or July 25, 2013).

^d^ HRs and 95% CIs correspond to time since diagnosis accounting for late entry into the risk set at the GENEVA enrollment date.

^e^ Includes Coast Guard (deaths: n = 6; total: n = 8), Activated National Guard (deaths: n = 5; total: n = 6), Activated Reserves (deaths: n = 11; total: n = 17), Inactivated National Guard (deaths: n < 5; total: n < 5), Inactivated Reserves (deaths: n = 8; total: n = 9), Department of Defense (deaths: n = 0; total: n = 0), National Oceanic and Atmospheric Administration (deaths: n = 0; total: n < 5), and Public Health Service (deaths: n = 0; total: n = 0). Any numbers presented as “n < 5” were suppressed to preserve the confidentiality of study participants.

^f^ Used within-category medians that were calculated using all cases.

^g^ Scaled the HR to an IQR-unit increase in the exposure variable. IQRs were calculated using all cases.

^h^ Category boundaries aligned with the occurrence of the major wars (e.g., the Vietnam War occurred between August 1964 and May 1975) and followed Allen et al. [14], Beard et al. [17], and Schmidt et al. [15].

**Table 4:** Military deployments or danger pay and amyotrophic lateral sclerosis survival in GENEVA^a^, United States of America, 2005–2013.

Variable	Deaths		Total		IP-weighted^b^	
	No.	PM^c^	No.	PM^c^	HR^d^	95% CI^d^
***Deployments***						
Ever deployed to any war/operation^e^						
No	250	5,790	348	13,683	1.00	Referent
Yes	179	4,472	247	9,986	0.98	0.78, 1.23
Missing	17		21			
War/operation of longest deployment^e^						
Not deployed	250	5,790	348	13,683	1.00	Referent
World War II	24	671	29	1,076	1.97	1.34, 2.87
Korean War	29	686	32	931	0.96	0.41, 2.25
Vietnam War	98	2,318	138	5,474	1.02	0.75, 1.40
Gulf War	< 5^f^	164	11	762	0.38	0.12, 1.18
Other^g^	15	421	24	1,164	0.81	0.43, 1.54
Missing	26		34			
Ever deployed to any other country						
No	222	5,222	309	12,310	1.00	Referent
Yes	205	5,014	283	11,254	1.01	0.81, 1.26
Missing	19		24			
Total time (years) of all periods of deployment to any war/operation^e^						
Not deployed (Median = 0.00)	250	5,790	348	13,683	1.00	Referent
≤ 1 (0.67)	88	2,206	120	4,834	1.10	0.86, 1.41
> 1–2 (1.25)	45	1,228	67	2,918	0.77	0.56, 1.06
> 2–4 (2.37)	29	643	36	1,238	1.45	1.01, 2.10
> 4 (6.00)	8	183	11	417	0.64	0.19, 2.23
Missing	26		34			
Trend^h^					0.87	0.71, 1.07
End of most recent period of deployment to any war/operation (month/year)^e^^,^ ^i^						
Not deployed	250	5,790	348	13,683	1.00	Referent
≤ 06/1950 (01/1946)	23	656	28	1,061	1.74	1.16, 2.60
07/1950-01/1955 (01/1953)	24	539	26	702	1.35	0.90, 2.03
02/1955-02/1961 (02/1956)	7	165	8	247	1.94	0.48, 7.75
03/1961-04/1975 (02/1969)	98	2,362	136	5,348	1.14	0.82, 1.58
05/1975-07/1990 (08/1980)	6	147	10	491	1.00	0.53, 1.87
> 07/1990 (07/1992)	12	391	25	1,462	0.58	0.30, 1.10
Missing	26		35			
Trend (IQR = 14 years, 11.98 months)^h^^,^ ^j^					0.65	0.48, 0.89
***Danger pay*, *hardship duty or combat zone tax exclusion benefits for deployment***						
Ever received imminent danger pay, hardship duty or combat zone tax exclusion benefits for deployment						
No	309	7,571	414	16,134	1.00	Referent
Yes	103	2,267	157	6,498	0.87	0.66, 1.15
Missing	34		45			
Total time (years) of all periods of deployment to any countries or sea region(s) ever received imminent danger pay, hardship duty or combat zone tax exclusion benefits for deployment						
Never received imminent danger pay, hardship duty or combat zone tax exclusion benefits for deployment (0.00)	309	7,571	414	16,134	1.00	Referent
≤ 1 (0.83)	47	1,092	73	3,104	0.80	0.52, 1.21
> 1–2 (1.13)	21	447	30	1,172	0.88	0.58, 1.33
> 2 (2.50)	8	191	11	462	1.00	0.48, 2.07
Missing	61		88			
Trend^h^					0.98	0.79, 1.22

Abbreviation: ALS, amyotrophic lateral sclerosis; ALSFRS-R, ALS Functional Rating Scale-Revised; CI, confidence interval; GENEVA, Genes and Environmental Exposures in Veterans with Amyotrophic Lateral Sclerosis study; Gulf, 1990–1991 Persian Gulf; HR, hazard ratio; IP, inverse probability; IQR, interquartile range; PM, person-months; VA, Department of Veterans Affairs.

^a^ Fourteen ALS cases were excluded from this analysis because (1) they were missing data on diagnosis date (n = 7) or (2) they died before GENEVA enrollment (i.e., enrollment was completed by proxy after the case died; n = 7).

^b^ Weighted for confounding (conditional on age [modeled with indicator variables corresponding to 5-year groups]), not missing baseline ALSFRS-R score (conditional on most recent ALS diagnosis category, symptom onset site, diagnostic delay [months; modeled with the natural logarithm of a linear term], and time from diagnosis to enrollment in the Registry [months; modeled with a restricted, quadratic spline with knots at 7.72, 13.24, 23.06, and 44.19 months based on percentiles of the distribution in the Registry cases not missing baseline ALSFRS-R score]), and participating in GENEVA (conditional on race/ethnicity, being a current patient of a VA Medical Center, most recent ALS diagnosis category, symptom onset site, diagnostic delay [months; modeled with linear, quadratic, and cubic terms], time from diagnosis to enrollment in the Registry [months; modeled with a linear term], and baseline ALSFRS-R score [modeled with a restricted, quadratic spline with knots at 12, 34, and 44 based on percentiles of the distribution in GENEVA cases]). 95% CIs were calculated with robust variance estimates.

^c^ Person-months calculated for time on study (i.e., the difference between the GENEVA enrollment date and the death date or July 25, 2013).

^d^ HRs and 95% CIs correspond to time since diagnosis accounting for late entry into the risk set at the GENEVA enrollment date.

^e^ The GENEVA study questionnaire asked "Were you deployed to…" the following wars where each war was asked about with a separate question: World War II (defined as the period from December 7, 1941, to December 31, 1946), the Korean War (defined as the period from June 27, 1950, to January 31, 1955), the Vietnam War (defined as the period from August 3, 1964, to May 7, 1975), and the Persian Gulf War (defined as the period from August 2, 1990, to December 31, 1991) [16]. The questionnaire also asked "Ever deployed…" to the following countries where each country was asked about with a separate question: Grenada, Lebanon, Panama, Somalia, Bosnia, Kosovo, Rwanda, Afghanistan, and Iraq/Persian Gulf region (Gulf War II) [16].

^f^ Suppressed to preserve the confidentiality of study participants.

^g^ Includes Grenada (deaths: n = 0; total: n = 0), Lebanon (deaths: n < 5^f^; total: n = 5), Panama (deaths: n < 5^f^; total: n = 8), Somalia (deaths: n < 5^f^; total: n < 5^f^), Bosnia (deaths: n < 5^f^; total: n < 5^f^), Kosovo (deaths: n = 0; total: n = 0), Rwanda (deaths: n = 0; total: n = 0), Afghanistan (deaths: n < 5^f^; total: n < 5^f^), and Iraq/Persian Gulf region (Gulf War II) (deaths: n = 5; total: n = 6).

^h^ Used within-category medians that were calculated using all cases.

^i^ Category boundaries aligned with the occurrence of the major wars (e.g., the Vietnam War occurred between August 1964 and May 1975) and followed Allen et al. [14], Beard et al. [17], and Schmidt et al. [15].

^j^ Scaled the HR to an IQR-unit increase in the exposure variable. IQRs were calculated using all cases except those in the reference category. Reference category excluded for linear trend test.

Table 5 presents weighted results for ever experiencing specific exposures during service or deployment to the four wars; weighted results for analyses of exposure-response trends and feeling ill after exposure are in Tables B-C in S1 File. For exposures experienced any time during service, the HR for ever receiving anthrax vaccination was elevated but there was no exposure-response trend nor was there an association with feeling ill after exposure. Survival was not associated with ever taking pyridostigmine bromide pills, but HRs were elevated for taking them more recently, on more total days, and for taking more total pills per day and overall; however, all of these exposure-response metrics had fewer than five exposed deaths in some categories. For exposures experienced during deployment to the four wars, HRs were elevated for mixing and application of burning agents and there was a positive exposure-response trend. In contrast, HRs were decreased for field exposure to riot control substances or burning agents and the latter had an inverse exposure-response trend. HRs were elevated for high-intensity radar wave exposure, being within one mile of any explosion, and feeling ill after any explosion within one mile, but exposure-response trends were unapparent. HRs were lower for exposure to: paint, solvents, or petrochemical substances; local food not provided by the Armed Forces; hearing chemical alarms sounding; and Agent Orange, and the first three had inverse exposure-response trends. Cases who felt ill after local food exposure also had lower HRs than those who did not experience this exposure.

**Table 5:** Military exposures and amyotrophic lateral sclerosis survival in GENEVA^a^, United States of America, 2005–2013.

Exposure	Deaths		Total^b^		IP-weighted^c^^,^ ^d^	
	No.	PM^e^	No.	PM^e^	HR^f^	95% CI^f^
Total	446	10,558	616	24,267		
Ever received the anthrax vaccine prior to reference date	25	534	35	1,372	1.62	1.10, 2.40
Ever received the smallpox vaccine	329	7,944	454	18,127	0.92	0.67, 1.26
Prior to reference date, ever involved in testing, transporting or spraying herbicides for military purposes	10	350	17	851	1.12	0.63, 1.99
Prior to reference date, ever been treated with nasopharyngeal (NP) radium during military service	< 5^g^	96	< 5^g^	179	0.97	0.59, 1.59
Ever taken pyridostigmine bromide, or little white pills in foil packs, sometimes called NAPPs, which are used to protect against nerve agents	10	296	18	940	1.21	0.42, 3.44
Prior to reference date, ever visited or resided in the island of Guam, the islands of New Guinea, or the Kii Peninsula of Japan (including any time spent there in the military)	66	1,619	90	3,550	1.00	0.76, 1.30
***While you were in WWII*, *the Korean War*, *the Vietnam War*, *or the Gulf War***^h^**: *did you have direct contact with/were you exposed to***	168	4,112	228	8,976		
Ionizing radiation from nuclear weapon testing or occupation of Hiroshima/Nagasaki	< 5^g^	18	< 5^g^	77	1.01	0.22, 4.75
Use of personal pesticides, like creams, sprays or flea collars	50	1,206	67	2,536	0.91	0.62, 1.33
Use of pesticides on your clothing or bedding	36	675	49	1,673	1.05	0.63, 1.77
Exhaust from heaters or generators (e.g., kerosene heaters, tent heaters)	52	1,213	74	3,035	0.81	0.53, 1.21
Exposure to diesel and/or other petrochemical fumes	102	2,499	143	5,750	1.06	0.66, 1.72
Burning trash or burning feces/manure	51	1,243	74	3,096	0.80	0.54, 1.19
Exposure to paint, solvents, or petrochemical substances	45	1,132	72	3,264	0.73	0.50, 1.06
High-intensity radar waves (e.g., as radar operator, radio operator, aviation electrician's mate)	35	815	46	1,692	1.32	0.91, 1.92
Food contaminated with smoke, oil, or other chemicals	11	267	15	580	0.71	0.36, 1.40
Local food other than food provided by the Armed Forces	77	2,056	112	4,873	0.68	0.49, 0.93
Bathing in or drinking of water contaminated with smoke, oil, dead animals or any chemicals	12	188	20	801	0.85	0.33, 2.21
Heat cramps, heat exhaustion, heat stroke or other heat Illness	34	811	50	2,103	0.88	0.54, 1.44
Heard chemical alarms sounding	7	169	15	810	0.59	0.25, 1.40
Explosion in the air or on the ground within one mile of you (e.g., artillery, rockets, mortars)	112	2,601	149	5,597	1.27	0.87, 1.84
Have you suffered a combat-related injury that required medical attention during your deployment?	38	1,015	49	1,871	0.88	0.54, 1.44
***While you were in WWII*, *the Korean War*, *or the Vietnam War***^h^**: *did you have direct contact with/were you exposed to***	164	3,992	216	8,164		
Mixing and application of herbicides	< 5^g^	87	< 5^g^	87	1.57	0.99, 2.51
Exposure to herbicides in the field	10	304	11	396	1.02	0.62, 1.66
Mixing and application of riot control substances	< 5^g^	9	< 5^g^	9	4.30	2.33, 7.94
Exposure to riot control substances in the field	9	267	12	490	0.63	0.32, 1.21
Mixing and application of burning agents	13	193	14	284	2.57	1.28, 5.12
Exposure to burning agents in the field	22	511	32	1,271	0.56	0.35, 0.91
***While you were in the Korean War*, *the Vietnam War*, *or the Gulf War***^h^**: *did you have direct contact with/were you exposed to***	141	3,384	194	7,659		
Microwave radiation	10	241	14	581	1.03	0.62, 1.69
***While you were in the Vietnam War***^h^**: *did you have direct contact with/were you exposed to***	104	2,492	145	5,739		
Mixing and application of Agent Orange	6	127	8	283	0.62	0.32, 1.20
Exposure to Agent Orange in the field	39	795	63	2,668	0.66	0.42, 1.05
***While you were in the Gulf War***^h^**: *did you have direct contact with/were you exposed to***	8	289	16	980		
Use of depleted uranium (DU) for munitions or armor	< 5^g^	43	5	389	0.61	0.15, 2.41
CARC (Chemical Agent Resistant Compound) paint	< 5^g^	69	< 5^g^	240	1.78	0.36, 8.94
Scud missile explosion in the air or on the ground within one mile of you	< 5^g^	78	< 5^g^	267	3.84	0.82, 18.00
Smoke from oil well fires	< 5^g^	68	9	665	0.25	0.06, 1.02
Exposure to nerve gas (e.g., during munitions destruction)	0	0	< 5^g^	281	^i^	^i^
High levels of dust/sand	5	202	13	894	0.15	0.01, 1.56
Ground level fumigation	0	0	< 5^g^	365	^i^	^i^
In any conflicts deployed to, any other exposure or experience not asked about which you consider harmful or extremely stressful	52	1,420	68	2,692	0.79	0.52, 1.21

Abbreviation: ALS, amyotrophic lateral sclerosis; ALSFRS-R, ALS Functional Rating Scale-Revised; CARC, Chemical Agent Resistant Compound; CI, confidence interval; DU, depleted uranium; GENEVA, Genes and Environmental Exposures in Veterans with Amyotrophic Lateral Sclerosis study; Gulf, 1990–1991 Persian Gulf; HR, hazard ratio; IP, inverse probability; NP, nasopharyngeal; PM, person-months; VA, Department of Veterans Affairs; WWII, World War II.

^a^ Fourteen ALS cases were excluded from this analysis because (1) they were missing data on diagnosis date (n = 7) or (2) they died before GENEVA enrollment (i.e., enrollment was completed by proxy after the case died; n = 7).

^b^ Information for specific exposures was missing for 0–49% of cases.

^c^ Cases who did not experience direct contact with each specific exposure were the reference.

^d^ Weighted for confounding (conditional on age [modeled with indicator variables corresponding to 5-year groups or, for exposures queried only in reference to deployment to the Gulf War, modeled with a linear term that was centered at 60—the median among all cases] and war/operation of longest deployment), not missing baseline ALSFRS-R score (conditional on most recent ALS diagnosis category, symptom onset site, diagnostic delay [months; modeled with the natural logarithm of a linear term], and time from diagnosis to enrollment in the Registry [months; modeled with a restricted, quadratic spline with knots at 7.72, 13.24, 23.06, and 44.19 months based on percentiles of the distribution in the Registry cases not missing baseline ALSFRS-R score]), and participating in GENEVA (conditional on race/ethnicity, being a current patient of a VA Medical Center, most recent ALS diagnosis category, symptom onset site, diagnostic delay [months; modeled with linear, quadratic, and cubic terms], time from diagnosis to enrollment in the Registry [months; modeled with a linear term], and baseline ALSFRS-R score [modeled with a restricted, quadratic spline with knots at 12, 34, and 44 based on percentiles of the distribution in GENEVA cases]). 95% CIs were calculated with robust variance estimates.

^e^ Person-months calculated for time on study (i.e., the difference between the GENEVA enrollment date and the death date or July 25, 2013).

^f^ HRs and 95% CIs correspond to time since diagnosis accounting for late entry into the risk set at the GENEVA enrollment date.

^g^ Suppressed to preserve the confidentiality of study participants.

^h^ The GENEVA study questionnaire asked "Were you deployed to…" the following wars where each war was asked about with a separate question: World War II (defined as the period from December 7, 1941, to December 31, 1946), the Korean War (defined as the period from June 27, 1950, to January 31, 1955), the Vietnam War (defined as the period from August 3, 1964, to May 7, 1975), and the Persian Gulf War (defined as the period from August 2, 1990, to December 31, 1991) [16].

^i^ Unable to estimate HR and 95% CI.

Sub-analyses restricted to veterans who were deployed to the Vietnam War generally showed similar results (Tables D-G in S1 File) as did restricting to reduce sample heterogeneity (Table H in S1 File), control confounding by sex, or mitigate potential bias from exposure misclassification. Using age as the timescale, with late entry at GENEVA enrollment date, produced similar results. Recoding implausible values to missing for exposures that had distinct time periods of use removed the association between anthrax vaccination and shorter survival (HR = 0.99; 95% CI: 0.43, 2.26), but gave qualitatively similar results for the other three exposures. The majority of exposures were either not or only weakly correlated (absolute value of coefficients ≤ 0.2), and results were similar after mutually-adjusting correlated exposures. Finally, adjusting for potential confounding via standard regression methods, but without weighting for potential missing-covariate-data or selection bias, gave results that were generally similar (“Adjusted” columns, Tables B-G and I-K in S1 File).

## Discussion

We found shorter survival among cases who served ≤ 1 year in the military, served or were deployed before July 1950 or in WWII, and mixed and applied burning agents. In contrast, survival was longer among cases who were exposed to: paint, solvents, or petrochemical substances; local food not provided by the Armed Forces; or burning agents and Agent Orange in the field. We obtained similar results from several secondary analyses, such as restricting to cases who: enrolled within two years of diagnosis; had definite or probable ALS; were men; or did not need a proxy interview.

Some evidence suggests ALS diagnoses may be increased among military veterans, particularly those deployed to the Gulf War [7–10]. However, few studies have evaluated associations between military-related or other environmental or occupational factors and ALS progression or survival, and they all have important limitations [4–6, 11, 12]. The study based on the ALS Clinical Assessment, Research and Education database [11] was published in abstract form only, so it is difficult to evaluate. The Massachusetts study [6], which also reported no association between one-year disease progression and exposure to several toxicants evaluated as a group, did not adjust for age at diagnosis, which is one of the most consistently reported prognostic factors for ALS survival [2–5]. This limitation is shared by a study in Italy which found agricultural work was not associated with cumulative or one-year survival [4]. The studies by Kasarskis et al. [12] and Pastula et al. [5], which examined war deployments, were susceptible to immortal person-time bias. The discrepant results of these studies may be because the first—which found a positive association—was restricted to Gulf War-era veterans [12], whereas the other—which found no association—included non-Gulf War-era veterans [5]. Like Pastula et al. [5], our study included a subset of Registry cases who served as far back as WWII. Unlike Pastula et al. [5], we found shorter survival among cases who were deployed to WWII, but not to other wars/operations, compared to non-deployed cases.

Our finding of an association between anthrax vaccination and shorter survival may be partly due to reporting errors; some veterans who reported receiving the vaccination served in the military in the 1950s [17], but the vaccination was not available until 1998 [46]. Recoding implausible values to missing removed the association. Our finding of shorter survival among cases who mixed and applied burning agents contrasts with the longer survival we observed among cases who experienced field exposure to burning agents. Mixing and applying, however, may have resulted in greater exposure than field exposure and was also likely less susceptible to recall bias. Our finding of positive exposure-response trends between pyridostigmine bromide pills and survival was limited by small numbers of deaths in exposure categories and should be interpreted cautiously. The elevated HR observed for high-intensity radar waves is consistent with reports that non-ionizing radiation may be associated with ALS etiology [47]. One explanation for associations we observed between longer survival and exposure to: paint, solvents, or petrochemical substances; local food; or Agent Orange in the field is individuals who were susceptible to these exposures may have died before Registry enrollment. However, we found no relationship between these exposures and diagnostic delay, symptom onset site, or baseline ALSFRS-R score, which were clinical factors consistently reported to be associated with shorter survival by previous studies [2–6].

Residual confounding by age may have affected our results because cases who served before July 1950 or were deployed to WWII were more than 70 years-old in 2005–2010 when GENEVA was conducted [15] and were thus more likely to die during follow-up. Using age as the timescale, with late entry at GENEVA enrollment date, however, gave similar results. It is also possible other exposures experienced during military service or civilian life confounded associations we observed between survival and specific exposures experienced during deployment to the four wars. We did not adjust for non-military exposures, but results were similar after mutually-adjusting correlated military exposures, which mitigates this concern.

The positive association we observed between ALS survival and time from enrollment in the Registry to enrollment in GENEVA is perplexing. This result was meant to be descriptive and, therefore, was adjusted for age at diagnosis via standard regression methods, but was not weighted for potential missing-covariate-data or selection biases. It is possible that this result is due to selection bias. In other words, it is possible that the patients with the fastest progressing ALS died after they enrolled in the Registry, but before they enrolled in GENEVA (Table A in S1 File). This would create the appearance of a positive association between ALS survival and time from enrollment in the Registry to enrollment in GENEVA among ALS cases who enrolled in GENEVA.

The four assumptions for using weights for analysis are consistency, exchangeability (i.e., no unmeasured confounding, missing data, or selection bias), no misspecification of models used to estimate the weights, and positivity [31]. Consistency is not empirically verifiable, but is often assumed to hold [31]. As described above, we explored exchangeability by conducting secondary analyses in which we restricted to men only and mutually-adjusted correlated military exposures. We attempted to minimize model misspecification by using the Akaike Information Criterion [48] to select the form (e.g., linear, categorical, spline, etc.) for each continuous variable included in our models for the weights [49] (Section C in S1 File). To explore positivity, we created cross-tabulations of the military-related factors and age and war/operation of deployment (for military exposures).

We also inspected appropriateness of the weights we used in our analyses. Most means of the overall stabilized weights were near one and there were few extreme weights. Exceptions generally occurred among time-related variables (e.g., end of most recent service or deployment). Cross-tabulations of the affected variables and age revealed empty cells—especially in extreme combinations of the variables and age. These instances of empty cells are likely structural rather than random [31], because, for example, WWII veterans would not have been ≤ 39 years-old when GENEVA was conducted. We remedied the problem by collapsing categories of both the affected variables and age and obtained HRs and 95% CIs that were similar to those we obtained when we used the expanded versions.

Our results do not generalize to veteran cases who died before enrollment in the Registry began in 2003 [5] or who did not join the Registry for other reasons [17] because we were only able to weight GENEVA cases to represent Registry cases. In addition, the median (IQR) time from enrollment in the Registry to enrollment in GENEVA was nine (11) months, which is a short interval relative to elapsed time since diagnosis for cases who were long-term survivors. Thus, our results could have been affected by residual selection bias.

There are limitations to studying cases from the Registry. For example, the Registry contains incident cases and prevalent cases that include some long-term survivors [15, 50]. In addition, as among non-deployed veterans in the VA’s previous Gulf War ALS study [8, 10], the Registry likely under-ascertained cases [17] notwithstanding a national recruiting effort [14]. Despite these limitations, we observed associations between clinical and demographic characteristics and ALS survival that agreed with previous literature—e.g., HRs for death decreased with increases in diagnostic delay [2, 5, 6] and baseline ALSFRS-R score [2, 3] and were higher among older cases [2–5]—increasing confidence in our results.

We collected information on military-related factors via standardized telephone interviews [15, 16] because of the lack of suitable exposure biomarkers and sufficiently detailed military databases for most exposures we evaluated in our study [17]. Several studies have evaluated the validity and reliability of many self-reported military-related factors, usually in comparison to data from the U.S. Department of Defense’s Defense Manpower Data Center, and reported concordances to range from “moderate” (e.g., κ = 0.41–0.60) to “almost perfect” (e.g., κ > 0.80) [10, 46, 51–58]. Validity studies evaluated deployment status and frequency, number, and start dates of deployment(s) [10, 56]; occupational titles [57]; and anthrax [46, 54] or smallpox vaccination [53]. Military-related factors evaluated in reliability studies included combat-related experiences, exposures, or traumatic events [51, 52, 55, 58] and exposure indices constructed by averaging or summing participants’ answers for specific items [51, 52, 55, 58]. Unfortunately, none of these studies assessed the validity or reliability of self-reported military-related factors extending back to WWII (the longest time between exposure and assessment of exposure in these studies was about 10 years [10]) or whether the quality of self-report fluctuated by time period of service or war/operation of deployment.

Although the validity and reliability of self-report has not been determined for many of the military-related factors we evaluated in our study, we would typically expect military service factors, such as branch of service or rank, to be more validly and reliably reported than military deployment factors, such as war/operation of deployment or dates of deployment, which would likely be more validly and reliably reported than specific military exposures [17]. In fact, this is the pattern typically described by other occupational epidemiology studies (i.e., job title is typically more validly and reliably reported than dates of employment or job tasks, which are typically more validly and reliably reported than specific occupational exposures) [59].

Strategies to improve recall for GENEVA included asking participants about exposures that can be sensed (e.g., heard chemical alarms sounding), using general terms (e.g., flea collar rather than specific pesticides), querying a list of specific exposures, and providing benchmarks against which respondents could compare their exposure levels (e.g., feeling ill after exposure) [15]. Despite using these strategies, we found some reporting errors for a few exposures with distinct time periods of use. When we set implausible values to missing, however, results were qualitatively similar for exposures other than anthrax vaccination. Importantly, results were similar after restricting to non-proxy interviews.

Strengths of our study include medical record-confirmed diagnoses and relatively complete ascertainment of deaths from VA sources. We also had a large sample size, although the number of deceased cases who experienced some exposures, particularly Gulf War-specific ones, was small. We had extensive information on military service, deployments, and 39 related exposures. Given the large number of exposures for which we evaluated associations with ALS survival and the hypothesis-generating nature of our study, it is possible that some of the associations we observed may not be true. However, we obtained similar results from sub-analyses that created a more homogeneous sample or attempted to reduce potential biases. Finally, we used inverse probability weights to explore and control potential biases from confounding, missing covariate data, and selection among a case group that included a disproportionate number of long-term survivors at enrollment.

## Conclusions

In conclusion, this is one of the first and largest studies, and the most complete, to date of ALS survival and non-clinical factors—specifically those related to military service. It includes both detailed exposure assessment and sophisticated analytic approaches to minimize bias. Although we observed associations with clinical and demographic characteristics that agreed with previous literature, most military-related factors were not associated with ALS survival. Associations we observed between shorter survival and certain exposures, such as burning agents, although novel and needing confirmation, are potentially important because of the large number of U.S. military veterans. Furthermore, they could help clinicians target interventions that may have the potential to lengthen survival among patients at greatest risk of shorter survival.

## Supporting information

S1 File (DOCX)
