# Supplementary material for: Military service, deployments, and exposures in relation to amyotrophic lateral sclerosis survival
Source: PLoS One. 2017 Oct 10;12(10):e0185751. doi: 10.1371/journal.pone.0185751 (PMC5634564; doi:10.1371/journal.pone.0185751)
Supplement: S1 File — (DOCX) [file pone.0185751.s001.docx]

**S1 Supporting Information**

Military Service, Deployments, and Exposures in Relation to Amyotrophic Lateral Sclerosis Survival

John D. Beard^1,2,#a^, Lawrence S. Engel,^1^ David B. Richardson,^1^ Marilie D. Gammon,^1^ Coleen Baird,^3^ David M. Umbach,^4^ Kelli D. Allen,^5,6^ Catherine L. Stanwyck,^5,7^ Jean Keller,^8^ Dale P. Sandler,^2^ Silke Schmidt,^7^ Freya Kamel^2*^

^1^ Department of Epidemiology, Gillings School of Global Public Health, University of North Carolina at Chapel Hill, Chapel Hill, North Carolina, United States of America

^2^ Epidemiology Branch, National Institute of Environmental Health Sciences, Research Triangle Park, North Carolina, United States of America

^3^ Environmental Medicine Program, US Army Public Health Command, Aberdeen Proving Ground, Maryland, United States of America

^4^ Biostatistics and Computational Biology Branch, National Institute of Environmental Health Sciences, Research Triangle Park, North Carolina, United States of America

^5^ Durham VA Medical Center, Durham, North Carolina, United States of America

^6^ Department of Medicine and Thurston Arthritis Research Center, University of North Carolina at Chapel Hill, Chapel Hill, North Carolina, United States of America

^7^ Department of Medicine, Duke University Medical Center, Durham, North Carolina, United States of America

^8^ Westat, Inc., Durham, North Carolina, United States of America

^#a^ Current Address: Department of Health Science, College of Life Sciences, Brigham Young University, Provo, Utah, United States of America

* Corresponding author

E-mail: kamel@niehs.nih.gov (FK)

**Table of Contents**

Section A. Exposure Assessment: Construction of Military Service,

Deployment, and Danger Pay Variables 5

Fig A. Directed acyclic graphs for: (a) military service and deployment

variables and ALS survival in GENEVA and (b) military exposure variables

and ALS survival in GENEVA, United States of America, 2005-2013. 7

Section B. Explanation of Fig A 7

Section C. Statistical Analyses: Inverse Probability Weights 8

Table A. Demographic, clinical, and military characteristics of ALS cases

in GENEVA and the Registry, United States of America, 2003-2010. 14

Table B. Exposure-response for military exposures and amyotrophic lateral

sclerosis survival in GENEVA, United States of America, 2005-2013. 20

Table C. Feel ill from military exposures and amyotrophic lateral sclerosis

survival in GENEVA, United States of America, 2005-2013. 30

Table D. Military deployments to the Vietnam War and amyotrophic lateral

sclerosis survival in GENEVA, United States of America, 2005-2013. 37

Table E. Military exposures during deployment to the Vietnam War and

amyotrophic lateral sclerosis survival in GENEVA, United States of America,

2005-2013. 39

Table F. Exposure-response for military exposures during deployment to the

Vietnam War and amyotrophic lateral sclerosis survival in GENEVA, United

States of America, 2005-2013. 43

Table G. Feel ill from military exposures during deployment to the Vietnam

War and amyotrophic lateral sclerosis survival in GENEVA, United States of

America, 2005-2013. 49

Table H. Selected military exposures and amyotrophic lateral sclerosis survival

in GENEVA, United States of America, 2005-2013, restricted to cases who

enrolled in GENEVA within two years of diagnosis and without weighting for

potential missing-covariate-data or selection bias. 54

Table I. Military service and amyotrophic lateral sclerosis survival in

GENEVA, United States of America, 2005-2013, without weighting for

potential missing-covariate-data or selection bias. 55

Table J. Military deployments or danger pay and amyotrophic lateral

sclerosis survival in GENEVA, United States of America, 2005-2013, without

weighting for potential missing-covariate-data or selection bias. 56

Table K. Military exposures and amyotrophic lateral sclerosis survival in

GENEVA, United States of America, 2005-2013, without weighting for

potential missing-covariate-data or selection bias. 58

References 60

Section A. Exposure Assessment: Construction of Military Service, Deployment, and Danger Pay Variables

To construct several of the military service, deployment, and danger pay variables we used for analysis, we incorporated information from several questions asked during standardized telephone interviews [[1-3](#_ENREF_1)]. To construct military service variables, we used information on branch and beginning and ending dates of each period of service to calculate the total time of each period of service [[2](#_ENREF_2)]. For veterans who reported more than one period of service in the same branch, we summed up the time served during each period to calculate the total time served in each branch [[2](#_ENREF_2)]. We then determined the branch of longest service by comparing the total time served in each branch [[2](#_ENREF_2)]. We also calculated number of branches of service [[2](#_ENREF_2)]. We calculated total service time (years) and determined the end (month/year) of most recent service using the information on beginning and ending dates of each period of service [[2](#_ENREF_2)].

For military deployment variables, we used information on deployment, theaters of operation, and beginning and ending dates of deployment to each theater of operation for World War II and the Korean, Vietnam, and 1990–1991 Persian Gulf Wars (hereafter “four wars”) to calculate the total time deployed to each theater reported [[2](#_ENREF_2)]. For veterans who reported more than one theater for a war, we summed up the time deployed to each theater to calculate the total time deployed to a war [[2](#_ENREF_2)]. We also had information on operations in Grenada, Lebanon, Panama, Somalia, Bosnia, Kosovo, Rwanda, Afghanistan, and Iraq/Persian Gulf region (Gulf War II)—including deployment, year of deployment, and total months deployed to each operation [[2](#_ENREF_2)]. We determined the war/operation of longest deployment by comparing the total time deployed to each war/operation [[2](#_ENREF_2)]. We also determined ever deployment to any war/operation from the information on deployment to the four wars and nine operations [[2](#_ENREF_2)]. We calculated total time of all deployments (years) using the information on total time deployed to each war/operation [[2](#_ENREF_2)]. Finally, we determined the end (month/year) of most recent deployment using the information on timing of deployment to each war/operation [[2](#_ENREF_2)].

To construct danger pay variables, we used information on beginning and ending dates of deployment to up to five foreign countries or sea regions (selected from a list of 17 foreign countries and five sea regions plus fill-in options) for which imminent danger pay, hardship duty, or combat zone tax exclusion benefits for deployment were received [[2](#_ENREF_2)]. We calculated total time (years) deployed to those countries/sea regions for which these benefits were received using the above information [[2](#_ENREF_2)].

Fig A. Directed acyclic graphs for: (a) military service and deployment variables and ALS survival in GENEVA [[3](#_ENREF_3)] and (b) military exposure variables and ALS survival in GENEVA [[3](#_ENREF_3)], United States of America, 2005-2013. Abbreviations: ALS, amyotrophic lateral sclerosis; GENEVA, Genes and Environmental Exposures in Veterans with Amyotrophic Lateral Sclerosis study.


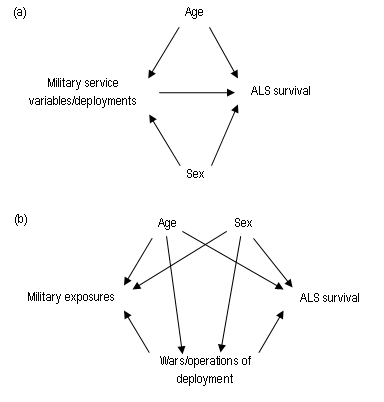


Section B. Explanation of Fig A

We identified the following prognostic factors for amyotrophic lateral sclerosis (ALS) survival from previous literature to consider as potential confounders: age at diagnosis, symptom onset site, clinical features (e.g., baseline ALS Functional Rating Scale-Revised [ALSFRS-R] score [[4](#_ENREF_4)]), time between symptom onset and diagnosis (diagnostic delay), diagnosis category, sex, psychosocial factors (e.g., mood), therapeutic interventions (e.g., riluzole treatment), agricultural occupation, and lead exposure. The directed acyclic graph (DAG) [[5](#_ENREF_5), [6](#_ENREF_6)] presented in panel (a) of Fig A applies to all analyses for military service and deployment factors. The arrows from age and sex to military service and deployment factors and ALS survival represent relationships between each covariate and military service and deployment factors and ALS survival, respectively. We did not include the following covariates in the DAG because they have not been associated with military service, deployments, and/or exposures previously: agricultural occupation, clinical features, diagnosis category, diagnostic delay, symptom onset site, and therapeutic interventions. We also thought that lead exposure and psychosocial factors were potentially *affected* by and/or occurred temporally *after* military service, deployments, and/or exposures, so we did not include them in the DAG.

The DAG presented in panel (b) of Fig A applies to all analyses for military exposures. The only difference between the DAGs presented in panels (a) and (b) of Fig A is the addition of wars/operations of deployment. Wars/operations of deployment is related to both military exposures and ALS survival.

Section C. Statistical Analyses: Inverse Probability Weights

As stated in the main text, we calculated three types of stabilized inverse probability weights (hereafter “weights”) [[7](#_ENREF_7), [8](#_ENREF_8)] to adjust for potential bias from (1) confounding by age and war/operation of deployment (for military exposures), (2) missing data on ALSFRS-R score for 8% of Registry cases, and (3) selection arising from a case group that included a disproportionate number of long-term survivors at enrollment in the Genes and Environmental Exposures in Veterans with Amyotrophic Lateral Sclerosis study (GENEVA) [[3](#_ENREF_3)] (Table A). Although we calculated confounding weights for each exposure separately, we used the same missing-covariate-data and selection weights for every exposure (selection weights were actually composites of a series of selection weights). We assessed appropriateness of the weights using established criteria [[7](#_ENREF_7)]. We calculated overall stabilized weights by multiplying the three types of weights together [[7](#_ENREF_7), [9](#_ENREF_9)]. We then applied the overall stabilized weight to Cox proportional hazards regression models that contained the exposure of interest as the only explanatory variable (sampling weights are applied in the same way when analyzing data from complex survey sampling designs) [[7](#_ENREF_7), [9](#_ENREF_9)].

For each exposure separately, we calculated stabilized confounding weights [[7](#_ENREF_7), [9](#_ENREF_9)] by fitting linear, logistic, or polytomous logistic regression models, depending on the nature of the exposure variable, to data for the 616 cases in GENEVA [[3](#_ENREF_3)] who were included in the current analysis. For the numerators of these weights, we calculated the predicted probabilities of exposure from intercept only models; and, for the denominators of the weights, we calculated the predicted probabilities conditional on age and war/operation of longest deployment (for military exposures). For the denominator models, we modeled age with indicator variables corresponding to five-year groups and war/operation of longest deployment with the categories shown in Table 4. For exposures queried only in reference to deployment to the 1990-1991 Persian Gulf War (hereafter “Gulf War”), we modeled age with a linear term that was centered at 60—the median among all cases.

We calculated two distinct types of stabilized selection weights [[7](#_ENREF_7), [9](#_ENREF_9)] by fitting logistic regression models to data for the 1,635 cases in the U.S. National Registry of Veterans with ALS (hereafter “Registry”) [[10](#_ENREF_10)] who: had diagnoses of clinically definite, probable, possible, or suspected ALS, were living at Registry enrollment (some enrolled posthumously by proxy), had diagnosis date available, and were not missing data on any covariates used to calculate the selection weights. One type of weights adjusted for potential selection bias from the death of ALS cases before they could enroll in GENEVA and the other type of weights adjusted for potential selection bias from all other reasons cases did not enroll in GENEVA (e.g., active refusal, unable to be contacted, etc.).

We calculated three weights for selection due to death, one for each of the three intervals during the GENEVA enrollment process in which cases died (Fig 1) (e.g., the time from when cases consented for the DNA Bank to when they were contacted regarding enrollment in GENEVA). We used dichotomous dependent variables (alive or dead at the end of the interval) for each interval weight. We used intercept only models to calculate the numerators of these weights as predicted probabilities of staying alive until the end of the interval of interest; we calculated the denominators of these weights as predicted probabilities conditional on race/ethnicity, being a current patient of a U.S. Department of Veterans Affairs Medical Center, most recent diagnosis category, symptom onset site, diagnostic delay, time from diagnosis to enrollment in the Registry, and baseline (i.e., at enrollment in the Registry) ALSFRS-R score [[4](#_ENREF_4)].

We calculated five weights for selection not related to death, one for each of the five intervals during the GENEVA enrollment process in which cases were lost (Fig 1). We used dichotomous dependent variables (e.g., consented or not for the DNA Bank, etc.) for each interval weight. We used intercept only models to calculate the numerators of these weights as predicted probabilities of participating in the enrollment step that occurred at the end of the interval (e.g., consented for the DNA Bank); we calculated the denominators of these weights as predicted probabilities conditional on the same covariates used for the death weights. For the three intervals in which cases died, we calculated the weights among cases who were still alive at the end of the interval.

Only the 616 cases who enrolled in GENEVA had available data on the exposures of interest. Therefore, we did not include the exposures of interest in numerator or denominator models for any of the selection weights. For the denominator models, we included race/ethnicity, being a current patient of a U.S. Department of Veterans Affairs Medical Center, most recent diagnosis category, and symptom onset site after categorizing them as shown in Tables 1 and 2. We modeled diagnostic delay with linear, quadratic, and cubic terms; time from diagnosis to enrollment in the Registry with a linear term; and baseline ALSFRS-R score with a restricted, quadratic spline with knots at 12, 34, and 44 based on percentiles of the distribution in GENEVA cases (we created the spline using SAS code from Howe et al. [[11](#_ENREF_11)]). These forms gave the lowest values of the Akaike Information Criterion [[11](#_ENREF_11), [12](#_ENREF_12)] among the many linear, quadratic, cubic, natural logarithm, categorical, and spline terms [[11](#_ENREF_11)] we considered. Finally, we obtained the overall stabilized selection weights by multiplying all eight selection weights together.

As mentioned previously, 8% of Registry cases were missing data on baseline ALSFRS-R score. This is important because we included baseline ALSFRS-R score as a covariate in the denominator models for all eight stabilized selection weights. Therefore, we needed to calculate stabilized weights for missing data on baseline ALSFRS-R score [[8](#_ENREF_8)]. We calculated these weights by fitting logistic regression models to data for the 1,791 Registry cases who: had diagnoses of clinically definite, probable, possible, or suspected ALS, were living at Registry enrollment, had diagnosis date available, and were not missing data on any covariates used to calculate the weights for missing data on baseline ALSFRS-R score. We used dichotomous dependent variables (missing data on baseline ALSFRS-R score or not). We used intercept only models to calculate the numerators of these weights as predicted probabilities of not missing data on baseline ALSFRS-R score; we calculated the denominators of these weights as predicted probabilities conditional on most recent diagnosis category, symptom onset site, diagnostic delay, and time from diagnosis to enrollment in the Registry.

As mentioned previously, only the 616 cases who enrolled in GENEVA had available data on the exposures of interest. Therefore, we did not include the exposures of interest in numerator or denominator models for the weights for missing data on ALSFRS-R score. For the denominator models, we included most recent diagnosis category and symptom onset site after categorizing them as shown in Table 2. We modeled diagnostic delay with the natural logarithm of a linear term and time from diagnosis to enrollment in the Registry with a restricted, quadratic spline with knots at 7.72, 13.24, 23.06, and 44.19 months based on percentiles of the distribution in the Registry cases not missing data on baseline ALSFRS-R score (we created the spline using SAS code from Howe et al. [[11](#_ENREF_11)]). These forms gave the lowest values of the Akaike Information Criterion [[11](#_ENREF_11), [12](#_ENREF_12)] among the many linear, quadratic, cubic, natural logarithm, categorical, and spline terms [[11](#_ENREF_11)] we considered.

We assessed the appropriateness of the weights we used in our analyses via four criteria: (1) mean weight near one; (2) few extreme weights (e.g., < 0.05 or > 20); (3) positivity (i.e., “exposed and unexposed individuals at every level of the confounders” [[7](#_ENREF_7)] or at every level of variables related to the outcome of interest and selection or missing data on baseline ALSFRS-R score); and (4) bias–variance tradeoff [[7](#_ENREF_7)]. Although the results of our assessment for the first three criteria are provided in the main text, our informal assessment of the bias-variance tradeoff [[13](#_ENREF_13), [14](#_ENREF_14)] is described here. We first progressively truncated the overall stabilized weights by symmetrically resetting weights less or greater than a certain percentile (e.g., 1st and 99th, 5th and 95th, 10th and 90th, etc.) to the value of that percentile [[7](#_ENREF_7)]. Regarding the hazard ratios derived from the untruncated weights as the “true” values, we then informally assessed bias-variance tradeoff by examining how attributes of both the weights (mean, minimum, maximum) and the corresponding hazard ratios (bias, variance) changed with increasing truncation. Not truncating the overall stabilized weights appeared to be the best balance of bias and variance in the current analysis.

**Table A. Demographic, clinical, and military characteristics of ALS cases in GENEVA and the Registry,** United States of America, 2003-2010**.** All information shown in this table came from medical records or the Registry screening or semi-annual telephone follow-up interviews [[1](#_ENREF_1), [3](#_ENREF_3)].

|  | GENEVA Cases | | Registry Cases | | Crude | | Adjusted^a^ | |
| --- | --- | --- | --- | --- | --- | --- | --- | --- |
| Characteristic | No. | % | No. | % | RR | 95% CI | RR | 95% CI |
| Total | 630 | 100 | 1,856 | 100 |  |  |  |  |
| Year of birth |  |  |  |  |  |  |  |  |
| ≤ 1920 | 5 | 1 | 35 | 2 | 0.40 | 0.17, 0.90 | NA | NA |
| 1921-1930 | 93 | 15 | 305 | 16 | 0.85 | 0.69, 1.03 | NA | NA |
| 1931-1940 | 194 | 31 | 573 | 31 | 0.94 | 0.80, 1.10 | NA | NA |
| 1941-1950 | 227 | 36 | 630 | 34 | 1.00 | Referent | 1.00 | Referent |
| 1951-1960 | 74 | 12 | 214 | 12 | 0.96 | 0.78, 1.19 | NA | NA |
| 1961-1970 | 32 | 5 | 87 | 5 | 1.02 | 0.76, 1.37 | NA | NA |
| > 1970 | 5 | 1 | 11 | 1 | 1.26 | 0.65, 2.43 | NA | NA |
| Missing | 0 |  | 1 |  |  |  |  |  |
| Median ± IQR | 1942 ± 14 yr. | | 1941 ± 16 yr. | |  |  |  |  |
| Sex |  |  |  |  |  |  |  |  |
| Male | 617 | 98 | 1,820 | 98 | 1.00 | Referent | 1.00 | Referent |
| Female | 13 | 2 | 35 | 2 | 1.10 | 0.71, 1.69 | 1.12 | 0.73, 1.73 |
| Missing | 0 |  | 1 |  |  |  |  |  |
| Race/Ethnicity |  |  |  |  |  |  |  |  |
| Non-Hispanic White | 581 | 92 | 1,674 | 90 | 1.00 | Referent | 1.00 | Referent |
| Other | 48 | 8 | 179 | 10 | 0.77 | 0.60, 0.99 | 0.75 | 0.58, 0.96 |
| Missing | 1 |  | 3 |  |  |  |  |  |
| Cigarette smoking status^b^ |  |  |  |  |  |  |  |  |
| Never | 217 | 35 | 552 | 33 | 1.00 | Referent | 1.00 | Referent |
| Past | 339 | 54 | 942 | 56 | 0.92 | 0.80, 1.05 | 0.89 | 0.78, 1.02 |
| Current | 70 | 11 | 195 | 12 | 0.91 | 0.74, 1.13 | 0.88 | 0.71, 1.09 |
| Missing | 4 |  | 167 |  |  |  |  |  |
| Being a current patient at a VA Medical Center |  |  |  |  |  |  |  |  |
| No | 295 | 47 | 786 | 42 | 1.00 | Referent | 1.00 | Referent |
| Yes | 335 | 53 | 1,068 | 58 | 0.84 | 0.74, 0.95 | 0.85 | 0.75, 0.96 |
| Missing | 0 |  | 2 |  |  |  |  |  |
|  |  |  |  |  |  |  |  |  |
|  |  |  |  |  |  |  |  |  |
|  |  |  |  |  |  |  |  |  |
|  |  |  |  |  |  |  |  |  |
| Most recent ALS diagnosis category^c, d^ |  |  |  |  |  |  |  |  |
| Clinically definite | 99 | 16 | 333 | 18 | 0.89 | 0.73, 1.08 | 0.87 | 0.71, 1.05 |
| Clinically probable | 244 | 39 | 728 | 39 | 1.00 | Referent | 1.00 | Referent |
| Clinically probable and lab supported | 113 | 18 | 277 | 15 | 1.22 | 1.02, 1.45 | 1.22 | 1.02, 1.45 |
| Clinically possible | 54 | 9 | 162 | 9 | 0.99 | 0.78, 1.27 | 1.02 | 0.80, 1.30 |
| Suspected (progressive bulbar palsy or progressive muscular atrophy) | 118 | 19 | 336 | 18 | 1.05 | 0.88, 1.25 | 1.08 | 0.91, 1.29 |
| Other | < 5^e^ | < 1 | 20 | 1 | 0.30 | 0.08, 1.12 | 0.29 | 0.08, 1.09 |
| Missing | 0 |  | 0 |  |  |  |  |  |
| Family history of ALS |  |  |  |  |  |  |  |  |
| No | 490 | 96 | 1,435 | 95 | 1.00 | Referent | 1.00 | Referent |
| Yes | 22 | 4 | 78 | 5 | 0.83 | 0.58, 1.19 | 0.81 | 0.56, 1.16 |
| Missing | 118 |  | 343 |  |  |  |  |  |
| Symptom onset site |  |  |  |  |  |  |  |  |
| Bulbar | 89 | 14 | 407 | 22 | 0.59 | 0.49, 0.72 | 0.59 | 0.49, 0.72 |
| Extremities | 509 | 81 | 1374 | 74 | 1.00 | Referent | 1.00 | Referent |
| Other^c^ | 31 | 5 | 71 | 4 | 1.18 | 0.90, 1.55 | 1.16 | 0.88, 1.52 |
| Missing | 1 |  | 4 |  |  |  |  |  |
| Time from symptom onset to diagnosis (months) |  |  |  |  |  |  |  |  |
| ≤ 6 | 118 | 19 | 410 | 23 | 0.95 | 0.78, 1.16 | 0.95 | 0.78, 1.16 |
| > 6-12 | 168 | 27 | 556 | 31 | 1.00 | Referent | 1.00 | Referent |
| > 12-18 | 105 | 17 | 301 | 17 | 1.15 | 0.95, 1.41 | 1.20 | 0.99, 1.47 |
| > 18-24 | 47 | 8 | 157 | 9 | 0.99 | 0.76, 1.30 | 1.05 | 0.80, 1.37 |
| > 24 | 173 | 28 | 378 | 21 | 1.51 | 1.28, 1.79 | 1.59 | 1.35, 1.88 |
| Missing | 19 |  | 54 |  |  |  |  |  |
| Median ± IQR | 13 ± 18 | | 11 ± 14 | |  |  |  |  |
| Time from diagnosis to enrollment in the Registry (months) |  |  |  |  |  |  |  |  |
| ≤ 12 | 220 | 35 | 664 | 36 | 1.00 | Referent | 1.00 | Referent |
| > 12-24 | 144 | 23 | 476 | 26 | 0.91 | 0.77, 1.09 | 0.92 | 0.77, 1.09 |
| > 24-36 | 75 | 12 | 241 | 13 | 0.94 | 0.76, 1.17 | 0.98 | 0.79, 1.22 |
| > 36-48 | 44 | 7 | 109 | 6 | 1.22 | 0.95, 1.57 | 1.21 | 0.94, 1.56 |
| > 48 | 140 | 22 | 343 | 19 | 1.23 | 1.04, 1.46 | 1.24 | 1.05, 1.46 |
| Missing | 7 |  | 23 |  |  |  |  |  |
| Median ± IQR | 18 ± 32 | | 18 ± 27 | |  |  |  |  |
|  |  |  |  |  |  |  |  |  |
|  |  |  |  |  |  |  |  |  |
|  |  |  |  |  |  |  |  |  |
| Baseline^d^ ALSFRS-R score (possible range: 0-48)^f^ |  |  |  |  |  |  |  |  |
| ≤ 16 | 58 | 9 | 309 | 18 | 0.66 | 0.49, 0.87 | 0.64 | 0.48, 0.85 |
| > 16-23 | 67 | 11 | 284 | 17 | 0.82 | 0.63, 1.07 | 0.82 | 0.63, 1.06 |
| > 23-30 | 104 | 17 | 363 | 21 | 1.00 | Referent | 1.00 | Referent |
| > 30-35 | 135 | 21 | 292 | 17 | 1.61 | 1.32, 1.98 | 1.59 | 1.30, 1.95 |
| > 35 | 265 | 42 | 454 | 27 | 2.04 | 1.70, 2.44 | 1.99 | 1.66, 2.38 |
| Missing | 1 |  | 154 |  |  |  |  |  |
| Median ± IQR | 34 ± 12 | | 29 ± 16 | |  |  |  |  |
| Currently using riluzole (baseline^d^) |  |  |  |  |  |  |  |  |
| No | 277 | 47 | 763 | 48 | 1.00 | Referent | 1.00 | Referent |
| Yes | 315 | 53 | 843 | 52 | 1.03 | 0.91, 1.17 | 1.02 | 0.89, 1.15 |
| Missing | 38 |  | 250 |  |  |  |  |  |
| Currently using BiPAP to assist with breathing (baseline^d^) |  |  |  |  |  |  |  |  |
| No | 531 | 86 | 1,336 | 79 | 1.00 | Referent | 1.00 | Referent |
| Yes | 89 | 14 | 351 | 21 | 0.64 | 0.53, 0.77 | 0.63 | 0.52, 0.76 |
| Missing | 10 |  | 169 |  |  |  |  |  |
| Currently using CPAP to assist with breathing (baseline^d^) |  |  |  |  |  |  |  |  |
| No | 603 | 97 | 1,620 | 96 | 1.00 | Referent | 1.00 | Referent |
| Yes | 17 | 3 | 59 | 4 | 0.77 | 0.52, 1.16 | 0.76 | 0.51, 1.14 |
| Missing | 10 |  | 177 |  |  |  |  |  |
| Currently using a ventilator (≥ two weeks, 15 hours/day) to assist with breathing (baseline^d^) |  |  |  |  |  |  |  |  |
| No | 595 | 95 | 1,568 | 93 | 1.00 | Referent | 1.00 | Referent |
| Yes | 30 | 5 | 123 | 7 | 0.64 | 0.47, 0.88 | 0.63 | 0.46, 0.87 |
| Missing | 5 |  | 165 |  |  |  |  |  |
| Currently using tracheostomy to assist with breathing (baseline^d^) |  |  |  |  |  |  |  |  |
| No | 555 | 95 | 1,389 | 92 | 1.00 | Referent | 1.00 | Referent |
| Yes | 32 | 5 | 120 | 8 | 0.67 | 0.49, 0.90 | 0.67 | 0.49, 0.91 |
| Missing | 43 |  | 347 |  |  |  |  |  |
| Currently using a feeding tube (baseline^d^) |  |  |  |  |  |  |  |  |
| No | 469 | 88 | 1,162 | 78 | 1.00 | Referent | 1.00 | Referent |
| Yes | 64 | 12 | 321 | 22 | 0.49 | 0.39, 0.62 | 0.49 | 0.39, 0.61 |
| Missing | 97 |  | 373 |  |  |  |  |  |
|  |  |  |  |  |  |  |  |  |
|  |  |  |  |  |  |  |  |  |
|  |  |  |  |  |  |  |  |  |
|  |  |  |  |  |  |  |  |  |
| Military branch of longest service |  |  |  |  |  |  |  |  |
| Air Force (including Army Air Force) | 126 | 20 | 350 | 19 | 1.06 | 0.90, 1.26 | 1.05 | 0.89, 1.25 |
| Army | 260 | 42 | 768 | 43 | 1.00 | Referent | 1.00 | Referent |
| Marines (including Merchant Marines) | 54 | 9 | 155 | 9 | 1.03 | 0.81, 1.30 | 1.04 | 0.82, 1.31 |
| Navy | 133 | 21 | 404 | 22 | 0.97 | 0.82, 1.15 | 0.98 | 0.83, 1.17 |
| Other^g^ | 47 | 8 | 125 | 7 | 1.11 | 0.87, 1.42 | 1.09 | 0.86, 1.40 |
| Missing | 10 |  | 54 |  |  |  |  |  |
| Number of military branches of service |  |  |  |  |  |  |  |  |
| 1 | 564 | 90 | 1,672 | 90 | 1.00 | Referent | 1.00 | Referent |
| 2 | 63 | 10 | 163 | 9 | 1.15 | 0.93, 1.41 | 1.12 | 0.91, 1.37 |
| > 2 | < 5^e^ | < 1 | 18 | 1 | 0.49 | 0.18, 1.39 | 0.52 | 0.18, 1.45 |
| Missing | 0 |  | 3 |  |  |  |  |  |
| Type duty (Active/Reserve) of longest service |  |  |  |  |  |  |  |  |
| Active | 541 | 88 | 1,586 | 88 | 1.00 | Referent | 1.00 | Referent |
| Active Reserves | 33 | 5 | 84 | 5 | 1.15 | 0.88, 1.52 | 1.11 | 0.84, 1.46 |
| Ready/Inactive Reserves or Reserves (unknown) | 44 | 7 | 124 | 7 | 1.04 | 0.81, 1.33 | 1.02 | 0.80, 1.31 |
| Missing | 12 |  | 62 |  |  |  |  |  |
| Number of duty types during service |  |  |  |  |  |  |  |  |
| 1 | 501 | 80 | 1,502 | 81 | 1.00 | Referent | 1.00 | Referent |
| 2 | 117 | 19 | 325 | 18 | 1.08 | 0.92, 1.27 | 1.04 | 0.89, 1.23 |
| 3 | 9 | 1 | 17 | 1 | 1.59 | 1.01, 2.50 | 1.55 | 0.99, 2.43 |
| Missing | 3 |  | 12 |  |  |  |  |  |
| Number of periods of military service |  |  |  |  |  |  |  |  |
| 1 | 484 | 77 | 1,441 | 78 | 1.00 | Referent | 1.00 | Referent |
| 2 | 124 | 20 | 344 | 19 | 1.07 | 0.92, 1.26 | 1.04 | 0.89, 1.22 |
| 3 | 19 | 3 | 53 | 3 | 1.07 | 0.74, 1.54 | 1.10 | 0.76, 1.58 |
| > 3 | < 5^e^ | < 1 | 16 | 1 | 0.56 | 0.20, 1.55 | 0.54 | 0.19, 1.50 |
| Missing | 0 |  | 2 |  |  |  |  |  |
| Years of military service |  |  |  |  |  |  |  |  |
| ≤ 1 | 18 | 3 | 58 | 3 | 0.92 | 0.62, 1.36 | 0.95 | 0.64, 1.41 |
| > 1-5 | 368 | 59 | 1,088 | 60 | 1.00 | Referent | 1.00 | Referent |
| > 5-10 | 97 | 16 | 308 | 17 | 0.93 | 0.77, 1.12 | 0.89 | 0.74, 1.07 |
| > 10-15 | 22 | 4 | 65 | 4 | 1.00 | 0.71, 1.42 | 0.96 | 0.67, 1.36 |
| > 15 | 115 | 19 | 283 | 16 | 1.20 | 1.02, 1.41 | 1.18 | 1.00, 1.39 |
| Missing | 10 |  | 54 |  |  |  |  |  |
| Median ± IQR | 4.00 ± 6.67 | | 4.00 ± 5.33 | |  |  |  |  |
| End of most recent period of military service (year)^h^ |  |  |  |  |  |  |  |  |
| ≤ 1949 | 46 | 7 | 173 | 10 | 0.78 | 0.60, 1.03 | 1.19 | 0.75, 1.89 |
| 1950-1954 | 52 | 8 | 159 | 9 | 0.96 | 0.75, 1.24 | 1.08 | 0.78, 1.50 |
| 1955-1960 | 79 | 13 | 248 | 14 | 0.94 | 0.76, 1.16 | 0.95 | 0.72, 1.23 |
| 1961-1964 | 64 | 10 | 166 | 9 | 1.14 | 0.91, 1.42 | 1.08 | 0.84, 1.39 |
| 1965-1974 | 193 | 31 | 569 | 32 | 1.00 | Referent | 1.00 | Referent |
| 1975-1980 | 47 | 8 | 137 | 8 | 1.01 | 0.78, 1.31 | 1.05 | 0.81, 1.36 |
| 1981-1990 | 59 | 10 | 173 | 10 | 1.01 | 0.79, 1.27 | 1.06 | 0.83, 1.35 |
| 1991-2001 | 62 | 10 | 136 | 8 | 1.34 | 1.08, 1.67 | 1.44 | 1.12, 1.84 |
| > 2001 | 16 | 3 | 38 | 2 | 1.24 | 0.84, 1.83 | 1.37 | 0.89, 2.11 |
| Missing | 12 |  | 57 |  |  |  |  |  |
| Median ± IQR | 1968 ± 21 yr. | | 1968 ± 19 yr. | |  |  |  |  |
| While in the military, served outside continental U.S. |  |  |  |  |  |  |  |  |
| No | 134 | 21 | 378 | 20 | 1.00 | Referent | 1.00 | Referent |
| Yes | 496 | 79 | 1,473 | 80 | 0.95 | 0.81, 1.11 | 0.95 | 0.82, 1.11 |
| Missing | 0 |  | 5 |  |  |  |  |  |
| Number of years served outside continental U.S. |  |  |  |  |  |  |  |  |
| 0 | 135 | 23 | 384 | 22 | 1.00 | Referent | 1.00 | Referent |
| ≤ 1 | 129 | 22 | 407 | 23 | 0.90 | 0.74, 1.10 | 0.89 | 0.73, 1.09 |
| > 1-2 | 181 | 30 | 518 | 30 | 0.99 | 0.83, 1.19 | 1.00 | 0.83, 1.19 |
| > 2-3 | 44 | 7 | 162 | 9 | 0.77 | 0.58, 1.03 | 0.78 | 0.58, 1.03 |
| > 3-5 | 49 | 8 | 126 | 7 | 1.11 | 0.85, 1.43 | 1.10 | 0.85, 1.43 |
| > 5 | 61 | 10 | 157 | 9 | 1.11 | 0.87, 1.40 | 1.10 | 0.86, 1.39 |
| Missing | 31 |  | 102 |  |  |  |  |  |
| Served in Afghanistan |  |  |  |  |  |  |  |  |
| No | 490 | 100 | 1,454 | 100 | 1.00 | Referent | 1.00 | Referent |
| Yes | < 5^e^ | < 1 | 6 | < 1 | 0.99 | 0.32, 3.07 | 1.05 | 0.34, 3.21 |
| Missing | 138 |  | 396 |  |  |  |  |  |
| Served in Europe |  |  |  |  |  |  |  |  |
| No | 281 | 57 | 850 | 58 | 1.00 | Referent | 1.00 | Referent |
| Yes | 213 | 43 | 615 | 42 | 1.05 | 0.91, 1.21 | 1.03 | 0.89, 1.19 |
| Missing | 136 |  | 391 |  |  |  |  |  |
| Served in Korea |  |  |  |  |  |  |  |  |
| No | 393 | 80 | 1,185 | 81 | 1.00 | Referent | 1.00 | Referent |
| Yes | 100 | 20 | 279 | 19 | 1.08 | 0.91, 1.29 | 1.08 | 0.90, 1.30 |
| Missing | 137 |  | 392 |  |  |  |  |  |
| Served in North Africa |  |  |  |  |  |  |  |  |
| No | 464 | 95 | 1,378 | 94 | 1.00 | Referent | 1.00 | Referent |
| Yes | 27 | 5 | 82 | 6 | 0.98 | 0.71, 1.34 | 1.00 | 0.73, 1.37 |
| Missing | 139 |  | 396 |  |  |  |  |  |
| Served in Pacific Islands |  |  |  |  |  |  |  |  |
| No | 334 | 68 | 1,039 | 71 | 1.00 | Referent | 1.00 | Referent |
| Yes | 158 | 32 | 424 | 29 | 1.16 | 1.00, 1.35 | 1.21 | 1.04, 1.40 |
| Missing | 138 |  | 393 |  |  |  |  |  |
| Served in Persian Gulf |  |  |  |  |  |  |  |  |
| No | 388 | 90 | 1,179 | 91 | 1.00 | Referent | 1.00 | Referent |
| Yes | 42 | 10 | 118 | 10 | 1.08 | 0.84, 1.40 | 0.98 | 0.73, 1.31 |
| Missing | 200 |  | 559 |  |  |  |  |  |
| Served in Vietnam |  |  |  |  |  |  |  |  |
| No | 358 | 72 | 1,059 | 72 | 1.00 | Referent | 1.00 | Referent |
| Yes | 137 | 28 | 410 | 28 | 0.99 | 0.84, 1.16 | 0.93 | 0.78, 1.12 |
| Missing | 135 |  | 387 |  |  |  |  |  |

Abbreviations: ALS, amyotrophic lateral sclerosis; ALSFRS-R, ALS Functional Rating Scale-Revised; BiPAP, Bi-level Positive Airway Pressure; CI, confidence interval; CPAP, Continuous Positive Airway Pressure; GENEVA, Genes and Environmental Exposures in Veterans with Amyotrophic Lateral Sclerosis; IQR, interquartile range; NA, not applicable; Registry, National Registry of Veterans with Amyotrophic Lateral Sclerosis; RR, risk ratio; U.S., United States; VA, Department of Veterans Affairs.

^a^ Adjusted for year of birth (modeled with a restricted, quadratic spline with three unequally spaced knots at 1925, 1942, and 1962 based on the 5th, 50th, and 95th percentiles of the distribution in all GENEVA cases).

^b^ Cigarette smoking was defined as having ever smoked at least 100 cigarettes (or the equivalent amount of tobacco) in cases’ lifetimes.

^c^ Includes “all over” (GENEVA: n < 5^e^, Registry: n = 11), “cramps/fasciculations” (GENEVA: n = 26; Registry: n = 57), “dementia” (GENEVA: n = 0; Registry: n < 5^e^), and “loss of appetite” (GENEVA: n < 5^e^; Registry: n < 5^e^).

^d^ The most recent diagnosis category, ALSFRS-R score, or use status of riluzole, BiPAP, CPAP, ventilator, tracheostomy, or feeding tube that was measured closest to the time of Registry enrollment.

^e^ Suppressed to preserve the confidentiality of study participants.

^f^ Category boundaries were set at quintiles of the ALSFRS-R score among all cases.

^g^ Includes Coast Guard (GENEVA: n = 7; Registry: n = 21), Air Force Reserves (GENEVA: n = 5; Registry: n = 8), Army Reserves (GENEVA: n = 9; Registry: n = 33), Marines Reserves (GENEVA: n < 5^e^; Registry: n < 5^e^), Navy Reserves (GENEVA: n = 8; Registry: n = 17), Air National Guard (GENEVA: n < 5^e^; Registry: n = 8), Army National Guard (GENEVA: n = 0; Registry: n = 0), Air Force Guard (GENEVA: n < 5^e^; Registry: n < 5^e^), Army Guard (GENEVA: n = 11; Registry: n = 31), and Other (GENEVA: n < 5^e^; Registry: n < 5^e^).

^h^ Category boundaries aligned with the occurrence of the major wars (e.g., the Vietnam War occurred between August 1964 and May 1975) and followed Allen et al. [[10](#_ENREF_10)], Beard et al. [[2](#_ENREF_2)], and Schmidt et al. [[3](#_ENREF_3)].

Table B. Exposure-response for military exposures and amyotrophic lateral sclerosis survival in GENEVA^a^, United States of America, 2005-2013.

|  | Deaths | | Total | | Adjusted^b^ | | Adjusted^c^ | | IP-weighted^d^ | |
| --- | --- | --- | --- | --- | --- | --- | --- | --- | --- | --- |
| Exposure | No. | PM^e^ | No. | PM^e^ | HR^f^ | 95% CI^f^ | HR^f^ | 95% CI^f^ | HR^f^ | 95% CI^f^ |
| Number of anthrax vaccine shots received prior to reference date |  |  |  |  |  |  |  |  |  |  |
| 0 (Median = 0) | 353 | 8,486 | 483 | 18,957 | 1.00 | Referent | 1.00 | Referent | 1.00 | Referent |
| 1-2 (1) | 8 | 206 | 10 | 382 | 1.48 | 0.71, 3.05 | 1.59 | 0.75, 3.37 | 1.25 | 0.67, 2.35 |
| > 2 (4) | 8 | 170 | 14 | 670 | 0.99 | 0.47, 2.06 | 1.11 | 0.50, 2.47 | 1.21 | 0.47, 3.13 |
| Missing | 51 |  | 75 |  |  |  |  |  |  |  |
| Trend^g^ |  |  |  |  | 1.01 | 0.85, 1.21 | 1.04 | 0.86, 1.26 | 1.05 | 0.83, 1.33 |
| Prior to reference date, number of years involved in testing, transporting or spraying herbicides for military purposes |  |  |  |  |  |  |  |  |  |  |
| 0 (0) | 325 | 7,357 | 440 | 16,184 | 1.00 | Referent | 1.00 | Referent | 1.00 | Referent |
| 1-2 (1) | 6 | 162 | 12 | 594 | 0.59 | 0.26, 1.34 | 0.60 | 0.26, 1.36 | 0.79 | 0.37, 1.71 |
| > 2 (10) | < 5^h^ | 188 | 5 | 257 | 1.56 | 0.55, 4.41 | 1.56 | 0.55, 4.44 | 1.50 | 0.90, 2.50 |
| Missing | 85 |  | 125 |  |  |  |  |  |  |  |
| Trend^g^ |  |  |  |  | 1.03 | 0.93, 1.15 | 1.04 | 0.93, 1.15 | 1.04 | 0.98, 1.09 |
| Year in which pyridostigmine bromide pills taken |  |  |  |  |  |  |  |  |  |  |
| Never taken pyridostigmine bromide pills | 379 | 9,072 | 523 | 20,649 | 1.00 | Referent | 1.00 | Referent | 1.00 | Referent |
| ≤ 1971 (1969.5) | 5 | 120 | 9 | 449 | 0.81 | 0.30, 2.22 | 0.83 | 0.30, 2.27 | 0.74 | 0.16, 3.37 |
| > 1971 (1991.0) | < 5^h^ | 123 | < 5^h^ | 123 | 1.23 | 0.49, 3.09 | 1.62 | 0.58, 4.54 | 2.54 | 1.17, 5.50 |
| Missing | 33 |  | 47 |  |  |  |  |  |  |  |
| Trend (IQR = 21)^g, i^ |  |  |  |  | ^j^ | ^j^ | ^j^ | ^j^ | 2.41 | 0.60, 9.73 |
| Total days taken pyridostigmine bromide pills |  |  |  |  |  |  |  |  |  |  |
| 0 (0) | 379 | 9,072 | 523 | 20,649 | 1.00 | Referent | 1.00 | Referent | 1.00 | Referent |
| 1-11 (4) | < 5^h^ | 125 | 5 | 198 | 0.74 | 0.30, 1.82 | 0.80 | 0.32, 1.97 | 1.16 | 0.37, 3.57 |
| > 11 (60) | < 5^h^ | 36 | 5 | 275 | 3.28 | 0.88, 12.20 | 4.77 | 1.21, 18.88 | 3.28 | 1.61, 6.68 |
| Missing | 35 |  | 49 |  |  |  |  |  |  |  |
| Trend^g^ |  |  |  |  | 1.02 | 1.00, 1.04 | 1.03 | 1.00, 1.05 | 1.02 | 1.01, 1.04 |
| Number of pills taken in an average day on days pyridostigmine bromide pills taken |  |  |  |  |  |  |  |  |  |  |
| 0 (0) | 379 | 9,072 | 523 | 20,649 | 1.00 | Referent | 1.00 | Referent | 1.00 | Referent |
| 1 (1) | < 5^h^ | 38 | 6 | 287 | 2.22 | 0.76, 6.46 | 2.56 | 0.88, 7.44 | 2.80 | 0.93, 8.45 |
| > 1 (2) | < 5^h^ | 123 | < 5^h^ | 123 | 0.60 | 0.15, 2.45 | 0.70 | 0.17, 2.99 | 1.69 | 0.58, 4.99 |
| Missing | 35 |  | 50 |  |  |  |  |  |  |  |
| Trend^g^ |  |  |  |  | 0.96 | 0.56, 1.64 | 1.05 | 0.60, 1.84 | 1.39 | 0.86, 2.24 |
|  |  |  |  |  |  |  |  |  |  |  |
| Total number of pyridostigmine bromide pills taken |  |  |  |  |  |  |  |  |  |  |
| 0 (0) | 379 | 9,072 | 523 | 20,649 | 1.00 | Referent | 1.00 | Referent | 1.00 | Referent |
| 1-22 (14) | < 5^h^ | 155 | 6 | 308 | 0.78 | 0.25, 2.47 | 0.89 | 0.27, 2.91 | 1.84 | 0.69, 4.92 |
| > 22 (60) | 5 | 139 | 9 | 472 | 3.35 | 0.89, 12.57 | 4.85 | 1.22, 19.34 | 3.68 | 1.70, 7.98 |
| Missing | 32 |  | 44 |  |  |  |  |  |  |  |
| Trend (33)^g, k^ |  |  |  |  | 1.75 | 0.84, 3.63 | 2.20 | 1.02, 4.73 | 2.23 | 1.32, 3.79 |
| Prior to reference date, number of times visited or resided in the island of Guam, the islands of New Guinea, or the Kii Peninsula of Japan |  |  |  |  |  |  |  |  |  |  |
| 0 (0) | 347 | 8,190 | 480 | 18,911 | 1.00 | Referent | 1.00 | Referent | 1.00 | Referent |
| 1 (1) | 35 | 848 | 45 | 1,634 | 1.20 | 0.84, 1.70 | 1.20 | 0.84, 1.71 | 1.21 | 0.85, 1.72 |
| 2 (2) | 9 | 272 | 14 | 679 | 0.80 | 0.41, 1.55 | 0.81 | 0.41, 1.60 | 0.77 | 0.42, 1.39 |
| 3 (3) | 8 | 124 | 13 | 531 | 0.80 | 0.39, 1.62 | 0.86 | 0.42, 1.75 | 1.03 | 0.55, 1.91 |
| 4 (4) | 5 | 81 | 6 | 165 | 1.09 | 0.44, 2.66 | 1.19 | 0.48, 3.00 | 0.97 | 0.42, 2.25 |
| > 4 (6) | 6 | 218 | 9 | 465 | 0.65 | 0.29, 1.46 | 0.66 | 0.29, 1.52 | 0.60 | 0.30, 1.22 |
| Missing | 10 |  | 15 |  |  |  |  |  |  |  |
| Trend^g^ |  |  |  |  | 0.95 | 0.86, 1.05 | 0.96 | 0.87, 1.06 | 0.96 | 0.87, 1.06 |
| Prior to reference date, total amount of time (months) spent in the island of Guam, the islands of New Guinea, or the Kii Peninsula of Japan, excluding time periods between age at first and last visits when you didn’t visit or reside in the island of Guam, the islands of New Guinea, or the Kii Peninsula of Japan |  |  |  |  |  |  |  |  |  |  |
| 0 (0.00) | 347 | 8,190 | 480 | 18,911 | 1.00 | Referent | 1.00 | Referent | 1.00 | Referent |
| > 0-3 (0.10) | 42 | 1,006 | 53 | 1,857 | 1.19 | 0.86, 1.64 | 1.22 | 0.87, 1.72 | 1.08 | 0.78, 1.49 |
| > 3-6 (5.14) | 10 | 252 | 16 | 729 | 0.69 | 0.37, 1.31 | 0.74 | 0.39, 1.41 | 0.88 | 0.39, 2.01 |
| > 6 (20.00) | 13 | 346 | 20 | 949 | 0.81 | 0.46, 1.41 | 0.82 | 0.46, 1.44 | 1.00 | 0.60, 1.65 |
| Missing | 8 |  | 13 |  |  |  |  |  |  |  |
| Trend (5.90)^g, k^ |  |  |  |  | 0.92 | 0.78, 1.08 | 0.92 | 0.78, 1.09 | 0.99 | 0.85, 1.15 |
|  |  |  |  |  |  |  |  |  |  |  |
|  |  |  |  |  |  |  |  |  |  |  |
|  |  |  |  |  |  |  |  |  |  |  |
|  |  |  |  |  |  |  |  |  |  |  |
|  |  |  |  |  |  |  |  |  |  |  |
|  |  |  |  |  |  |  |  |  |  |  |
| ***While you were in WWII, the Korean War, the Vietnam War, and/or the Gulf War^l^:*** |  |  |  |  |  |  |  |  |  |  |
| Total number of preventive vaccinations  received by injection (shots) or by mouth  while inside or outside the U.S. |  |  |  |  |  |  |  |  |  |  |
| 0 (0) | 43 | 1,119 | 56 | 2,208 | 1.00 | Referent | 1.00 | Referent | 1.00 | Referent |
| 1-10 (5) | 46 | 1,170 | 67 | 2,844 | 0.91 | 0.59, 1.43 | 0.95 | 0.60, 1.48 | 0.96 | 0.60, 1.54 |
| 11-20 (14) | 20 | 573 | 25 | 984 | 1.13 | 0.65, 1.97 | 1.22 | 0.69, 2.16 | 0.97 | 0.61, 1.56 |
| > 20 (66) | 15 | 295 | 23 | 919 | 0.87 | 0.47, 1.61 | 0.87 | 0.47, 1.61 | 0.81 | 0.46, 1.42 |
| Missing | 44 |  | 57 |  |  |  |  |  |  |  |
| Trend (13)^g, k^ |  |  |  |  | 0.98 | 0.87, 1.10 | 0.97 | 0.87, 1.09 | 0.96 | 0.86, 1.07 |
| ***While you were in WWII, the Korean War, the Vietnam War, and/or the Gulf War^l^: number of days exposed to*** |  |  |  |  |  |  |  |  |  |  |
| Ionizing radiation from nuclear weapon  testing or occupation of Hiroshima/Nagasaki |  |  |  |  |  |  |  |  |  |  |
| Not exposed | 153 | 3,911 | 209 | 8,450 | 1.00 | Referent | 1.00 | Referent | 1.00 | Referent |
| ≤ 5 | < 5^h^ | 17 | < 5^h^ | 76 | 1.18 | 0.28, 4.91 | 1.42 | 0.33, 6.01 | 1.07 | 0.22, 5.06 |
| 6-30 | < 5^h^ | 1 | < 5^h^ | 1 | 58.10 | 3.32, 1016.77 | 45.88 | 2.37, 888.40 | 45.03 | 4.96, 408.67 |
| > 30 | 0 | 0 | 0 | 0 | ^j^ | ^j^ | ^j^ | ^j^ | ^j^ | ^j^ |
| Missing | 12 |  | 15 |  |  |  |  |  |  |  |
| Trend^m^ |  |  |  |  | 1.23 | 1.06, 1.42 | 1.22 | 1.05, 1.42 | 1.19 | 1.04, 1.37 |
| Use of personal pesticides, like creams,  sprays or flea collars |  |  |  |  |  |  |  |  |  |  |
| Not exposed | 111 | 2,781 | 152 | 6,155 | 1.00 | Referent | 1.00 | Referent | 1.00 | Referent |
| ≤ 5 | 7 | 119 | 9 | 292 | 1.61 | 0.73, 3.54 | 1.72 | 0.78, 3.82 | 1.19 | 0.59, 2.39 |
| 6-30 | 7 | 134 | 8 | 207 | 1.59 | 0.69, 3.65 | 1.52 | 0.66, 3.51 | 1.56 | 0.99, 2.46 |
| > 30 | 34 | 860 | 48 | 1,944 | 1.13 | 0.75, 1.72 | 1.16 | 0.76, 1.77 | 0.80 | 0.47, 1.35 |
| Missing | 9 |  | 11 |  |  |  |  |  |  |  |
| Trend^n^ |  |  |  |  | 1.05 | 0.88, 1.25 | 1.06 | 0.88, 1.26 | 0.91 | 0.73, 1.13 |
| Use of pesticides on your clothing or bedding |  |  |  |  |  |  |  |  |  |  |
| Not exposed | 114 | 3,090 | 159 | 6,770 | 1.00 | Referent | 1.00 | Referent | 1.00 | Referent |
| ≤ 30 | 11 | 204 | 14 | 443 | 1.58 | 0.81, 3.09 | 1.52 | 0.78, 2.98 | 1.48 | 0.85, 2.57 |
| > 30 | 25 | 471 | 34 | 1,138 | 1.46 | 0.90, 2.35 | 1.41 | 0.87, 2.29 | 1.04 | 0.55, 1.97 |
| Missing | 18 |  | 21 |  |  |  |  |  |  |  |
| Trend^n^ |  |  |  |  | 1.19 | 0.98, 1.46 | 1.18 | 0.96, 1.44 | 1.03 | 0.79, 1.35 |
|  |  |  |  |  |  |  |  |  |  |  |
| Exhaust from heaters or generators (e.g.,  kerosene heaters, tent heaters) |  |  |  |  |  |  |  |  |  |  |
| Not exposed | 102 | 2,484 | 140 | 5,525 | 1.00 | Referent | 1.00 | Referent | 1.00 | Referent |
| ≤ 30 | 13 | 296 | 18 | 717 | 1.00 | 0.54, 1.85 | 0.97 | 0.52, 1.81 | 0.72 | 0.31, 1.67 |
| > 30 | 35 | 782 | 49 | 1,908 | 0.97 | 0.65, 1.45 | 1.00 | 0.66, 1.51 | 0.82 | 0.55, 1.23 |
| Missing | 18 |  | 21 |  |  |  |  |  |  |  |
| Trend^n^ |  |  |  |  | 0.99 | 0.84, 1.18 | 1.01 | 0.84, 1.20 | 0.92 | 0.77, 1.10 |
| Exposure to diesel and/or other  petrochemical fumes |  |  |  |  |  |  |  |  |  |  |
| Not exposed | 57 | 1,372 | 75 | 2,890 | 1.00 | Referent | 1.00 | Referent | 1.00 | Referent |
| ≤ 5 | 5 | 111 | 8 | 344 | 0.82 | 0.32, 2.07 | 0.88 | 0.34, 2.27 | 0.70 | 0.27, 1.80 |
| 6-30 | 9 | 146 | 14 | 535 | 0.95 | 0.45, 1.97 | 0.93 | 0.45, 1.95 | 0.88 | 0.40, 1.93 |
| > 30 | 84 | 2,120 | 117 | 4,749 | 1.01 | 0.70, 1.46 | 1.05 | 0.73, 1.52 | 1.09 | 0.69, 1.74 |
| Missing | 13 |  | 14 |  |  |  |  |  |  |  |
| Trend^n^ |  |  |  |  | 1.01 | 0.87, 1.18 | 1.03 | 0.88, 1.20 | 1.05 | 0.87, 1.27 |
| Burning trash or burning feces/manure |  |  |  |  |  |  |  |  |  |  |
| Not exposed | 108 | 2,691 | 144 | 5,635 | 1.00 | Referent | 1.00 | Referent | 1.00 | Referent |
| ≤ 5 | 11 | 224 | 11 | 224 | 2.15 | 1.10, 4.21 | 2.01 | 1.02, 3.96 | 1.63 | 0.92, 2.90 |
| 6-30 | 8 | 171 | 13 | 548 | 0.75 | 0.36, 1.56 | 0.70 | 0.33, 1.46 | 0.62 | 0.31, 1.25 |
| > 30 | 30 | 787 | 47 | 2,168 | 1.10 | 0.69, 1.76 | 1.10 | 0.69, 1.76 | 0.95 | 0.54, 1.64 |
| Missing | 11 |  | 13 |  |  |  |  |  |  |  |
| Trend^n^ |  |  |  |  | 1.00 | 0.82, 1.21 | 1.00 | 0.82, 1.21 | 0.94 | 0.74, 1.20 |
| Exposure to paint, solvents, or petrochemical  substances |  |  |  |  |  |  |  |  |  |  |
| Not exposed | 110 | 2,730 | 143 | 5,461 | 1.00 | Referent | 1.00 | Referent | 1.00 | Referent |
| ≤ 30 | 16 | 297 | 25 | 1,008 | 0.80 | 0.46, 1.38 | 0.81 | 0.47, 1.41 | 1.01 | 0.64, 1.61 |
| > 30 | 29 | 835 | 47 | 2,256 | 0.71 | 0.47, 1.09 | 0.74 | 0.48, 1.15 | 0.54 | 0.34, 0.88 |
| Missing | 13 |  | 13 |  |  |  |  |  |  |  |
| Trend^n^ |  |  |  |  | 0.89 | 0.75, 1.07 | 0.91 | 0.76, 1.09 | 0.80 | 0.66, 0.98 |
| High-intensity radar waves (e.g., as radar  operator, radio operator, aviation electrician's  mate) |  |  |  |  |  |  |  |  |  |  |
| Not exposed | 117 | 3,026 | 158 | 6,377 | 1.00 | Referent | 1.00 | Referent | 1.00 | Referent |
| ≤ 30 | 6 | 154 | 7 | 237 | 1.00 | 0.43, 2.34 | 1.19 | 0.49, 2.85 | 1.14 | 0.69, 1.89 |
| > 30 | 28 | 655 | 38 | 1,449 | 1.18 | 0.76, 1.84 | 1.26 | 0.81, 1.96 | 1.29 | 0.80, 2.09 |
| Missing | 17 |  | 25 |  |  |  |  |  |  |  |
| Trend^n^ |  |  |  |  | 1.08 | 0.90, 1.31 | 1.11 | 0.92, 1.34 | 1.12 | 0.91, 1.37 |
| Food contaminated with smoke, oil, or other  chemicals |  |  |  |  |  |  |  |  |  |  |
| Not exposed or ≤ 5 | 132 | 3,339 | 183 | 7,462 | 1.00 | Referent | 1.00 | Referent | 1.00 | Referent |
| > 5 | 9 | 219 | 13 | 532 | 0.92 | 0.46, 1.87 | 0.84 | 0.41, 1.71 | 0.68 | 0.32, 1.43 |
| Missing | 27 |  | 32 |  |  |  |  |  |  |  |
| Trend^n^ |  |  |  |  | 1.03 | 0.73, 1.45 | 0.98 | 0.69, 1.40 | 0.93 | 0.71, 1.23 |
| Local food other than food provided by the  Armed Forces |  |  |  |  |  |  |  |  |  |  |
| Not exposed | 85 | 1,881 | 108 | 3,741 | 1.00 | Referent | 1.00 | Referent | 1.00 | Referent |
| ≤ 5 | 22 | 653 | 33 | 1,614 | 0.86 | 0.53, 1.39 | 0.84 | 0.52, 1.37 | 0.78 | 0.50, 1.19 |
| 6-30 | 20 | 463 | 31 | 1,329 | 0.77 | 0.46, 1.28 | 0.76 | 0.45, 1.26 | 0.46 | 0.27, 0.80 |
| > 30 | 32 | 859 | 45 | 1,849 | 0.86 | 0.55, 1.34 | 0.88 | 0.56, 1.40 | 0.59 | 0.35, 0.98 |
| Missing | 9 |  | 11 |  |  |  |  |  |  |  |
| Trend^n^ |  |  |  |  | 0.94 | 0.78, 1.14 | 0.95 | 0.78, 1.16 | 0.78 | 0.62, 0.98 |
| Bathing in or drinking of water contaminated  with smoke, oil, dead animals or any  chemicals |  |  |  |  |  |  |  |  |  |  |
| Not exposed | 131 | 3,186 | 174 | 6,641 | 1.00 | Referent | 1.00 | Referent | 1.00 | Referent |
| ≤ 30 | 7 | 131 | 10 | 357 | 0.97 | 0.45, 2.13 | 0.96 | 0.44, 2.09 | 0.96 | 0.35, 2.65 |
| > 30 | 5 | 57 | 10 | 445 | 0.79 | 0.31, 2.01 | 0.78 | 0.31, 1.97 | 0.72 | 0.23, 2.25 |
| Missing | 25 |  | 34 |  |  |  |  |  |  |  |
| Trend^n^ |  |  |  |  | 0.92 | 0.63, 1.35 | 0.91 | 0.62, 1.34 | 0.91 | 0.57, 1.45 |
| Heat cramps, heat exhaustion, heat stroke or  other heat illness |  |  |  |  |  |  |  |  |  |  |
| Not exposed | 129 | 3,162 | 172 | 6,645 | 1.00 | Referent | 1.00 | Referent | 1.00 | Referent |
| ≤ 5 | 14 | 275 | 21 | 875 | 1.30 | 0.73, 2.32 | 1.36 | 0.76, 2.44 | 1.24 | 0.73, 2.08 |
| 6-30 | 9 | 283 | 17 | 916 | 0.58 | 0.29, 1.17 | 0.58 | 0.29, 1.17 | 0.45 | 0.20, 1.01 |
| > 30 | 11 | 253 | 12 | 312 | 2.31 | 1.20, 4.42 | 2.33 | 1.20, 4.53 | 2.17 | 1.25, 3.76 |
| Missing | 5 |  | 6 |  |  |  |  |  |  |  |
| Trend^n^ |  |  |  |  | 1.24 | 0.93, 1.66 | 1.23 | 0.92, 1.66 | 1.18 | 0.88, 1.58 |
| Heard chemical alarms sounding |  |  |  |  |  |  |  |  |  |  |
| Not exposed or ≤ 5 | 156 | 3,762 | 212 | 8,307 | 1.00 | Referent | 1.00 | Referent | 1.00 | Referent |
| > 5 | 5 | 128 | 9 | 447 | 0.68 | 0.27, 1.68 | 0.91 | 0.33, 2.46 | 0.88 | 0.40, 1.95 |
| Missing | 7 |  | 7 |  |  |  |  |  |  |  |
| Trend^n^ |  |  |  |  | 0.56 | 0.26, 1.20 | 0.61 | 0.28, 1.36 | 0.62 | 0.35, 1.09 |
|  |  |  |  |  |  |  |  |  |  |  |
| Explosion in the air or on the ground within  one mile of you (e.g., artillery, rockets,  mortars) |  |  |  |  |  |  |  |  |  |  |
| Not exposed | 49 | 1,349 | 71 | 3,154 | 1.00 | Referent | 1.00 | Referent | 1.00 | Referent |
| ≤ 5 | 27 | 678 | 33 | 1,196 | 1.74 | 1.06, 2.87 | 1.68 | 1.01, 2.78 | 1.70 | 1.11, 2.60 |
| 6-30 | 25 | 403 | 37 | 1,377 | 1.29 | 0.78, 2.14 | 1.18 | 0.70, 1.99 | 1.34 | 0.81, 2.21 |
| > 30 | 56 | 1,433 | 73 | 2,761 | 1.22 | 0.81, 1.83 | 1.19 | 0.79, 1.79 | 1.12 | 0.72, 1.74 |
| Missing | 11 |  | 14 |  |  |  |  |  |  |  |
| Trend^n^ |  |  |  |  | 1.01 | 0.87, 1.19 | 1.01 | 0.86, 1.18 | 0.96 | 0.81, 1.14 |
| Have you suffered a combat-related injury  that required medical attention during your  deployment? |  |  |  |  |  |  |  |  |  |  |
| Not exposed | 126 | 2,958 | 175 | 6,965 | 1.00 | Referent | 1.00 | Referent | 1.00 | Referent |
| ≤ 5 | 17 | 490 | 21 | 823 | 1.05 | 0.62, 1.81 | 1.04 | 0.60, 1.79 | 0.97 | 0.61, 1.56 |
| 6-30 | 7 | 225 | 10 | 442 | 0.86 | 0.39, 1.87 | 0.81 | 0.37, 1.78 | 0.68 | 0.28, 1.67 |
| > 30 | 13 | 294 | 16 | 507 | 1.28 | 0.71, 2.30 | 1.20 | 0.66, 2.19 | 1.47 | 0.76, 2.84 |
| Missing | 5 |  | 6 |  |  |  |  |  |  |  |
| Trend^n^ |  |  |  |  | 1.09 | 0.85, 1.40 | 1.06 | 0.82, 1.37 | 1.14 | 0.86, 1.51 |
| ***While you were in WWII, the Korean War, and/or the Vietnam War^l^: number of days exposed to*** |  |  |  |  |  |  |  |  |  |  |
| Mixing and application of herbicides |  |  |  |  |  |  |  |  |  |  |
| Not exposed | 150 | 3,661 | 201 | 7,744 | 1.00 | Referent | 1.00 | Referent | 1.00 | Referent |
| ≤ 5 | < 5^h^ | 6 | < 5^h^ | 6 | 3.33 | 0.42, 26.46 | 2.08 | 0.22, 19.57 | 2.33 | 0.87, 6.26 |
| 6-30 | < 5^h^ | 16 | < 5^h^ | 16 | 2.08 | 0.28, 15.54 | 2.09 | 0.28, 15.66 | 1.61 | 1.03, 2.51 |
| > 30 | < 5^h^ | 65 | < 5^h^ | 65 | 2.31 | 0.53, 10.03 | 1.98 | 0.44, 8.85 | 4.28 | 1.92, 9.50 |
| Missing | 10 |  | 11 |  |  |  |  |  |  |  |
| Trend^n^ |  |  |  |  | 1.47 | 0.81, 2.66 | 1.38 | 0.75, 2.53 | 1.83 | 1.34, 2.50 |
| Exposure to herbicides in the field |  |  |  |  |  |  |  |  |  |  |
| Not exposed or ≤ 5 | 123 | 2,991 | 162 | 6,134 | 1.00 | Referent | 1.00 | Referent | 1.00 | Referent |
| > 5 | 5 | 106 | 6 | 198 | 1.36 | 0.54, 3.41 | 1.20 | 0.47, 3.06 | 1.23 | 0.54, 2.81 |
| Missing | 36 |  | 48 |  |  |  |  |  |  |  |
| Trend^n^ |  |  |  |  | 1.32 | 0.84, 2.07 | 1.26 | 0.79, 2.01 | 1.24 | 0.87, 1.75 |
|  |  |  |  |  |  |  |  |  |  |  |
|  |  |  |  |  |  |  |  |  |  |  |
|  |  |  |  |  |  |  |  |  |  |  |
|  |  |  |  |  |  |  |  |  |  |  |
| Mixing and application of riot control  substances |  |  |  |  |  |  |  |  |  |  |
| Not exposed | 159 | 3,911 | 211 | 8,084 | 1.00 | Referent | 1.00 | Referent | 1.00 | Referent |
| ≤ 5 | < 5^h^ | 6 | < 5^h^ | 6 | 4.99 | 0.62, 40.15 | 4.28 | 0.53, 34.47 | 3.77 | 1.78, 7.99 |
| 6-30 | < 5^h^ | 3 | < 5^h^ | 3 | 4.13 | 0.47, 36.33 | 5.62 | 0.62, 50.86 | 5.45 | 1.73, 17.20 |
| > 30 | 0 | 0 | 0 | 0 | ^j^ | ^j^ | ^j^ | ^j^ | ^j^ | ^j^ |
| Missing | 3 |  | 3 |  |  |  |  |  |  |  |
| Trend^m^ |  |  |  |  | 1.09 | 0.97, 1.22 | 1.11 | 0.99, 1.25 | 1.10 | 1.03, 1.17 |
| Exposure to riot control substances in the  field |  |  |  |  |  |  |  |  |  |  |
| Not exposed or ≤ 5 | 155 | 3,728 | 204 | 7,675 | 1.00 | Referent | 1.00 | Referent | 1.00 | Referent |
| > 5 | 6 | 193 | 8 | 352 | 0.93 | 0.40, 2.15 | 0.98 | 0.42, 2.27 | 0.84 | 0.38, 1.86 |
| Missing | 3 |  | 4 |  |  |  |  |  |  |  |
| Trend^n^ |  |  |  |  | 1.01 | 0.58, 1.76 | 1.03 | 0.59, 1.80 | 0.88 | 0.48, 1.62 |
| Mixing and application of burning agents |  |  |  |  |  |  |  |  |  |  |
| Not exposed | 145 | 3,601 | 195 | 7,594 | 1.00 | Referent | 1.00 | Referent | 1.00 | Referent |
| ≤ 30 | 8 | 94 | 8 | 94 | 2.40 | 1.11, 5.21 | 2.61 | 1.20, 5.64 | 1.90 | 1.28, 2.84 |
| > 30 | 5 | 99 | 6 | 190 | 1.95 | 0.72, 5.34 | 2.13 | 0.79, 5.77 | 2.54 | 1.12, 5.79 |
| Missing | 6 |  | 7 |  |  |  |  |  |  |  |
| Trend^n^ |  |  |  |  | 1.45 | 0.99, 2.12 | 1.51 | 1.03, 2.20 | 1.52 | 1.10, 2.12 |
| Exposure to burning agents in the field |  |  |  |  |  |  |  |  |  |  |
| Not exposed | 134 | 3,286 | 175 | 6,610 | 1.00 | Referent | 1.00 | Referent | 1.00 | Referent |
| ≤ 5 | 8 | 167 | 14 | 596 | 0.59 | 0.28, 1.21 | 0.61 | 0.29, 1.26 | 0.40 | 0.17, 0.97 |
| > 5 | 11 | 319 | 15 | 649 | 0.94 | 0.49, 1.81 | 0.98 | 0.51, 1.89 | 0.87 | 0.50, 1.53 |
| Missing | 11 |  | 12 |  |  |  |  |  |  |  |
| Trend^n^ |  |  |  |  | 0.84 | 0.56, 1.24 | 0.86 | 0.58, 1.28 | 0.82 | 0.64, 1.04 |
| ***While you were in the Korean War, the Vietnam War, and/or the Gulf War^l^: number of days exposed to*** |  |  |  |  |  |  |  |  |  |  |
| Microwave radiation |  |  |  |  |  |  |  |  |  |  |
| Not exposed or ≤ 5 | 109 | 2,810 | 153 | 6,369 | 1.00 | Referent | 1.00 | Referent | 1.00 | Referent |
| > 5 | 9 | 223 | 12 | 472 | 1.17 | 0.57, 2.40 | 1.17 | 0.57, 2.41 | 1.08 | 0.65, 1.80 |
| Missing | 23 |  | 29 |  |  |  |  |  |  |  |
| Trend^n^ |  |  |  |  | 1.04 | 0.75, 1.46 | 1.04 | 0.74, 1.46 | 1.02 | 0.81, 1.29 |
|  |  |  |  |  |  |  |  |  |  |  |
|  |  |  |  |  |  |  |  |  |  |  |
|  |  |  |  |  |  |  |  |  |  |  |
| ***While you were in the Vietnam War^l^: number of days exposed to*** |  |  |  |  |  |  |  |  |  |  |
| Mixing and application of Agent Orange |  |  |  |  |  |  |  |  |  |  |
| Not exposed | 91 | 2,145 | 128 | 5,050 | 1.00 | Referent | 1.00 | Referent | 1.00 | Referent |
| ≤ 5 | < 5^h^ | 3 | < 5^h^ | 63 | 0.61 | 0.08, 4.50 | NA | NA | 0.24 | 0.02, 3.64 |
| 6-30 | < 5^h^ | 46 | < 5^h^ | 142 | 1.04 | 0.23, 4.70 | NA | NA | 1.03 | 0.23, 4.62 |
| > 30 | < 5^h^ | 59 | < 5^h^ | 59 | 1.31 | 0.31, 5.53 | NA | NA | 0.66 | 0.33, 1.36 |
| Missing | 8 |  | 10 |  |  |  |  |  |  |  |
| Trend^n^ |  |  |  |  | 1.10 | 0.61, 2.00 | NA | NA | 0.84 | 0.61, 1.16 |
| Exposure to Agent Orange in the field |  |  |  |  |  |  |  |  |  |  |
| Not exposed | 40 | 841 | 51 | 1,729 | 1.00 | Referent | 1.00 | Referent | 1.00 | Referent |
| ≤ 5 | 7 | 200 | 12 | 602 | 0.58 | 0.25, 1.32 | NA | NA | 0.68 | 0.33, 1.39 |
| 6-30 | 7 | 128 | 10 | 342 | 0.92 | 0.38, 2.22 | NA | NA | 0.70 | 0.27, 1.84 |
| > 30 | 22 | 408 | 35 | 1,442 | 0.82 | 0.47, 1.43 | NA | NA | 0.69 | 0.39, 1.23 |
| Missing | 28 |  | 37 |  |  |  |  |  |  |  |
| Trend^n^ |  |  |  |  | 0.96 | 0.76, 1.21 | NA | NA | 0.87 | 0.68, 1.11 |
| ***While you were in the Gulf War^l^: number of days exposed to*** |  |  |  |  |  |  |  |  |  |  |
| Use of depleted uranium (DU) for munitions  or armor |  |  |  |  |  |  |  |  |  |  |
| Not exposed or ≤ 30 | 5 | 213 | 10 | 671 | 1.00 | Referent | 1.00 | Referent | 1.00 | Referent |
| > 30 | < 5^h^ | 43 | < 5^h^ | 213 | 1.03 | 0.09, 11.80 | NA | NA | 0.80 | 0.17, 3.88 |
| Missing | 2 |  | 3 |  |  |  |  |  |  |  |
| Trend^n^ |  |  |  |  | 0.88 | 0.29, 2.64 | NA | NA | 0.62 | 0.28, 1.39 |
| CARC (Chemical Agent Resistant  Compound) paint |  |  |  |  |  |  |  |  |  |  |
| Not exposed | 5 | 205 | 10 | 663 | 1.00 | Referent | 1.00 | Referent | 1.00 | Referent |
| ≤ 5 | 0 | 0 | 0 | 0 | ^j^ | ^j^ | NA | NA | ^j^ | ^j^ |
| 6-30 | < 5^h^ | 27 | < 5^h^ | 27 | ^j^ | ^j^ | NA | NA | 1.00 | 0.06, 15.99 |
| > 30 | 0 | 0 | < 5^h^ | 170 | ^j^ | ^j^ | NA | NA | ^j^ | ^j^ |
| Missing | 2 |  | 3 |  |  |  |  |  |  |  |
| Trend^n^ |  |  |  |  | 0.50 | 0.07, 3.44 | NA | NA | 0.65 | 0.23, 1.83 |
|  |  |  |  |  |  |  |  |  |  |  |
|  |  |  |  |  |  |  |  |  |  |  |
|  |  |  |  |  |  |  |  |  |  |  |
|  |  |  |  |  |  |  |  |  |  |  |
|  |  |  |  |  |  |  |  |  |  |  |
| Scud missile explosion in the air or on the  ground within one mile of you |  |  |  |  |  |  |  |  |  |  |
| Not exposed | < 5^h^ | 153 | 9 | 593 | 1.00 | Referent | 1.00 | Referent | 1.00 | Referent |
| ≤ 5 | < 5^h^ | 78 | < 5^h^ | 172 | 36.01 | 0.66, 1973.21 | NA | NA | 0.04 | 0.00, 0.62 |
| 6-30 | 0 | 0 | < 5^h^ | 94 | ^j^ | ^j^ | NA | NA | ^j^ | ^j^ |
| > 30 | 0 | 0 | 0 | 0 | ^j^ | ^j^ | NA | NA | ^j^ | ^j^ |
| Missing | 2 |  | 3 |  |  |  |  |  |  |  |
| Trend^n^ |  |  |  |  | 1.28 | 0.01, 248.17 | NA | NA | 11.40 | 0.32, 409.94 |
| Smoke from oil well fires |  |  |  |  |  |  |  |  |  |  |
| Not exposed | 5 | 206 | 5 | 206 | 1.00 | Referent | 1.00 | Referent | 1.00 | Referent |
| ≤ 5 | < 5^h^ | 27 | < 5^h^ | 214 | ^j^ | ^j^ | NA | NA | ^j^ | ^j^ |
| 6-30 | 0 | 0 | < 5^h^ | 240 | ^j^ | ^j^ | NA | NA | ^j^ | ^j^ |
| > 30 | 0 | 0 | < 5^h^ | 169 | ^j^ | ^j^ | NA | NA | ^j^ | ^j^ |
| Missing | 2 |  | 3 |  |  |  |  |  |  |  |
| Trend^n^ |  |  |  |  | ^j^ | ^j^ | NA | NA | ^j^ | ^j^ |
| Exposure to nerve gas (e.g., during munitions  destruction) |  |  |  |  |  |  |  |  |  |  |
| Not exposed | 7 | 246 | 11 | 593 | 1.00 | Referent | 1.00 | Referent | 1.00 | Referent |
| ≤ 5 | 0 | 0 | < 5^h^ | 93 | ^j^ | ^j^ | NA | NA | ^j^ | ^j^ |
| 6-30 | 0 | 0 | < 5^h^ | 94 | ^j^ | ^j^ | NA | NA | 1.00 | 0.06, 15.99 |
| > 30 | 0 | 0 | < 5^h^ | 94 | ^j^ | ^j^ | NA | NA | ^j^ | ^j^ |
| Missing | 1 |  | 2 |  |  |  |  |  |  |  |
| Trend^n^ |  |  |  |  | ^j^ | ^j^ | NA | NA | ^j^ | ^j^ |
| High levels of dust/sand |  |  |  |  |  |  |  |  |  |  |
| Not exposed | < 5^h^ | 86 | < 5^h^ | 86 | 1.00 | Referent | 1.00 | Referent | 1.00 | Referent |
| ≤ 5 | < 5^h^ | 55 | < 5^h^ | 138 | ^j^ | ^j^ | NA | NA | ^j^ | ^j^ |
| 6-30 | 0 | 0 | < 5^h^ | 264 | ^j^ | ^j^ | NA | NA | ^j^ | ^j^ |
| > 30 | < 5^h^ | 111 | 7 | 455 | ^j^ | ^j^ | NA | NA | ^j^ | ^j^ |
| Missing | 1 |  | 1 |  |  |  |  |  |  |  |
| Trend^m^ |  |  |  |  | 1.39 | 0.41, 4.73 | NA | NA | 1.54 | 1.49, 1.59 |
| Ground level fumigation |  |  |  |  |  |  |  |  |  |  |
| Not exposed | 6 | 204 | 9 | 468 | 1.00 | Referent | 1.00 | Referent | 1.00 | Referent |
| ≤ 5 | 0 | 0 | < 5^h^ | 93 | ^j^ | ^j^ | NA | NA | ^j^ | ^j^ |
| 6-30 | 0 | 0 | < 5^h^ | 83 | ^j^ | ^j^ | NA | NA | ^j^ | ^j^ |
| > 30 | 0 | 0 | < 5^h^ | 188 | ^j^ | ^j^ | NA | NA | ^j^ | ^j^ |
| Missing | 2 |  | 3 |  |  |  |  |  |  |  |
| Trend^n^ |  |  |  |  | ^j^ | ^j^ | NA | NA | ^j^ | ^j^ |

Abbreviations: ALS, amyotrophic lateral sclerosis; ALSFRS-R, ALS Functional Rating Scale-Revised; CARC, Chemical Agent Resistant Compound; CI, confidence interval; DU, depleted uranium; GENEVA, Genes and Environmental Exposures in Veterans with Amyotrophic Lateral Sclerosis; Gulf, 1990-1991 Persian Gulf; HR, hazard ratio; IP, inverse probability; IQR, interquartile range; NA, not applicable; PM, person-months; U.S., United States of America; VA, Department of Veterans Affairs; WWII, World War II.

^a^ Fourteen ALS cases were excluded from this analysis because (1) they were missing data on diagnosis date (n = 7) or (2) they died before GENEVA enrollment (i.e., enrollment was completed by proxy after the case died; n = 7).

^b^ Adjusted for age (modeled with indicator variables corresponding to 5-year groups or, for exposures queried only in reference to deployment to the Gulf War, modeled with a linear term that was centered at age 60—the median age among all cases).

^c^ Adjusted for age (modeled with indicator variables corresponding to 5-year groups), war/operation of longest deployment, and the pairwise interaction between war/operation of longest deployment and the natural logarithm of time since diagnosis.

^d^ Weighted for confounding (conditional on age [modeled with indicator variables corresponding to 5-year groups or, for exposures queried only in reference to deployment to the Gulf War, modeled with a linear term that was centered at age 60—the median age among all cases] and war/operation of longest deployment), not missing baseline ALSFRS-R score (conditional on most recent ALS diagnosis category, symptom onset site, diagnostic delay [months; modeled with the natural logarithm of a linear term], and time from diagnosis to enrollment in the Registry [months; modeled with a restricted, quadratic spline with knots at 7.72, 13.24, 23.06, and 44.19 months based on percentiles of the distribution in the Registry cases not missing baseline ALSFRS-R score]), and participating in GENEVA (conditional on race/ethnicity, being a current patient of a VA Medical Center, most recent ALS diagnosis category, symptom onset site, diagnostic delay [months; modeled with linear, quadratic, and cubic terms], time from diagnosis to enrollment in the Registry [months; modeled with a linear term], and baseline ALSFRS-R score [modeled with a restricted, quadratic spline with knots at 12, 34, and 44 based on percentiles of the distribution in GENEVA cases]). 95% CIs were calculated with robust variance estimates.

^e^ Person-months calculated for time on study (i.e., the difference between the GENEVA enrollment date and the death date or July 25, 2013).

^f^ HRs and 95% CIs correspond to time since diagnosis accounting for late entry into the risk set at the GENEVA enrollment date.

^g^ Used within-category medians that were calculated using all cases.

^h^ Suppressed to preserve the confidentiality of study participants.

^i^ Scaled the HR to an IQR-unit increase in the exposure variable. IQRs were calculated using all cases except those in the reference category. Reference category excluded for linear trend test.

^j^ Unable to estimate HR and 95% CI.

^k^ Scaled the HR to an IQR-unit increase in the exposure variable. IQRs were calculated using all cases except those in the reference category. Reference category included for linear trend test.

^l^ The GENEVA study questionnaire asked "Were you deployed to..." the following wars where each war was asked about with a separate question: World War II (defined as the period from December 7, 1941, to December 31, 1946), the Korean War (defined as the period from June 27, 1950, to January 31, 1955), the Vietnam War (defined as the period from August 3, 1964, to May 7, 1975), and the Persian Gulf War (defined as the period from August 2, 1990, to December 31, 1991) [[1](#_ENREF_1)].

^m^ Used category midpoints (0, 3, 18) or 50% above the lower bound of the highest category (46.5). HR corresponds to a one-day increase in the exposure variable. Reference category included for linear trend test.

^n^ Used category midpoints (0, 3, 18) or 50% above the lower bound of the highest category (46.5) and scaled the HR to a 20-day increase in the exposure variable. Reference category included for linear trend test.

Table C. Feel ill from military exposures and amyotrophic lateral sclerosis survival in GENEVA^a^, United States of America, 2005-2013.

|  | Deaths | | | Total | | | Adjusted^b^ | | | Adjusted^c^ | | | IP-weighted^d^ | |  |
| --- | --- | --- | --- | --- | --- | --- | --- | --- | --- | --- | --- | --- | --- | --- | --- |
| Exposure | No. | PM^e^ | No. | | PM^e^ | HR^f^ | | 95% CI^f^ | HR^f^ | | 95% CI^f^ | HR^f^ | | 95% CI^f^ | |
| ***While you were in WWII, the Korean War, the Vietnam War, and/or the Gulf War^g^: did you feel ill from exposure to*** |  |  |  | |  |  | |  |  | |  |  | |  | |
| Ionizing radiation from nuclear weapon testing or occupation of  Hiroshima/Nagasaki |  |  |  | |  |  | |  |  | |  |  | |  | |
| Not exposed | 153 | 3,911 | 209 | | 8,450 | 1.00 | | Referent | 1.00 | | Referent | 1.00 | | Referent | |
| No | < 5^h^ | 18 | < 5^h^ | | 77 | 1.78 | | 0.55, 5.77 | 2.18 | | 0.66, 7.18 | 1.39 | | 0.34, 5.70 | |
| Yes | 0 | 0 | 0 | | 0 | ^j^ | | ^j^ | ^j^ | | ^j^ | ^j^ | | ^j^ | |
| Missing | 12 |  | 15 | |  |  | |  |  | |  |  | |  | |
| Trend^i^ |  |  |  | |  | 1.78 | | 0.55, 5.77 | 2.18 | | 0.66, 7.18 | 1.00 | | 0.15, 6.60 | |
| Use of personal pesticides, like creams, sprays or flea collars |  |  |  | |  |  | |  |  | |  |  | |  | |
| Not exposed | 111 | 2,781 | 152 | | 6,155 | 1.00 | | Referent | 1.00 | | Referent | 1.00 | | Referent | |
| No | 42 | 1,083 | 54 | | 2,018 | 1.33 | | 0.90, 1.96 | 1.34 | | 0.90, 1.98 | 0.91 | | 0.63, 1.32 | |
| Yes | < 5^h^ | 66 | 5 | | 131 | 1.14 | | 0.41, 3.14 | 1.26 | | 0.45, 3.55 | 0.42 | | 0.07, 2.74 | |
| Missing | 11 |  | 17 | |  |  | |  |  | |  |  | |  | |
| Trend^i^ |  |  |  | |  | 1.22 | | 0.89, 1.67 | 1.25 | | 0.91, 1.72 | 1.01 | | 0.68, 1.50 | |
| Use of pesticides on your clothing or bedding |  |  |  | |  |  | |  |  | |  |  | |  | |
| Not exposed | 114 | 3,090 | 159 | | 6,770 | 1.00 | | Referent | 1.00 | | Referent | 1.00 | | Referent | |
| No | 29 | 539 | 39 | | 1,307 | 1.42 | | 0.91, 2.21 | 1.37 | | 0.87, 2.14 | 0.93 | | 0.53, 1.61 | |
| Yes | < 5^h^ | 63 | 5 | | 203 | 1.23 | | 0.38, 4.01 | 1.21 | | 0.37, 3.96 | 0.45 | | 0.06, 3.34 | |
| Missing | 22 |  | 25 | |  |  | |  |  | |  |  | |  | |
| Trend^i^ |  |  |  | |  | 1.29 | | 0.90, 1.85 | 1.26 | | 0.87, 1.82 | 1.06 | | 0.58, 1.93 | |
| Exhaust from heaters or generators (e.g., kerosene heaters, tent  heaters) |  |  |  | |  |  | |  |  | |  |  | |  | |
| Not exposed | 102 | 2,484 | 140 | | 5,525 | 1.00 | | Referent | 1.00 | | Referent | 1.00 | | Referent | |
| No | 44 | 1,090 | 56 | | 2,093 | 1.16 | | 0.80, 1.70 | 1.16 | | 0.79, 1.70 | 0.98 | | 0.71, 1.36 | |
| Yes | 6 | 105 | 14 | | 745 | 0.50 | | 0.22, 1.16 | 0.51 | | 0.22, 1.21 | 0.33 | | 0.10, 1.13 | |
| Missing | 16 |  | 18 | |  |  | |  |  | |  |  | |  | |
| Trend^i^ |  |  |  | |  | 0.90 | | 0.68, 1.18 | 0.90 | | 0.68, 1.20 | 0.77 | | 0.54, 1.11 | |
| Exposure to diesel and/or other petrochemical fumes |  |  |  | |  |  | |  |  | |  |  | |  | |
| Not exposed | 57 | 1,372 | 75 | | 2,890 | 1.00 | | Referent | 1.00 | | Referent | 1.00 | | Referent | |
| No | 78 | 1,925 | 107 | | 4,165 | 0.97 | | 0.67, 1.40 | 1.01 | | 0.69, 1.46 | 1.02 | | 0.63, 1.65 | |
| Yes | 18 | 437 | 25 | | 1,020 | 1.34 | | 0.76, 2.36 | 1.39 | | 0.78, 2.46 | 1.20 | | 0.61, 2.39 | |
| Missing | 15 |  | 21 | |  |  | |  |  | |  |  | |  | |
| Trend^i^ |  |  |  | |  | 1.10 | | 0.83, 1.44 | 1.12 | | 0.85, 1.48 | 1.06 | | 0.78, 1.45 | |
|  |  |  |  | |  |  | |  |  | |  |  | |  | |
|  |  |  |  | |  |  | |  |  | |  |  | |  | |
| Burning trash or burning feces/manure |  |  |  | |  |  | |  |  | |  |  | |  | |
| Not exposed | 108 | 2,691 | 144 | | 5,635 | 1.00 | | Referent | 1.00 | | Referent | 1.00 | | Referent | |
| No | 40 | 1,103 | 58 | | 2,562 | 1.06 | | 0.71, 1.58 | 1.04 | | 0.69, 1.55 | 0.80 | | 0.54, 1.19 | |
| Yes | 9 | 126 | 13 | | 436 | 1.43 | | 0.69, 2.96 | 1.36 | | 0.65, 2.81 | 0.70 | | 0.23, 2.15 | |
| Missing | 11 |  | 13 | |  |  | |  |  | |  |  | |  | |
| Trend^i^ |  |  |  | |  | 1.13 | | 0.83, 1.53 | 1.10 | | 0.81, 1.50 | 0.85 | | 0.61, 1.18 | |
| Exposure to paint, solvents, or petrochemical substances |  |  |  | |  |  | |  |  | |  |  | |  | |
| Not exposed | 110 | 2,730 | 143 | | 5,461 | 1.00 | | Referent | 1.00 | | Referent | 1.00 | | Referent | |
| No | 38 | 1,017 | 60 | | 2,763 | 0.78 | | 0.54, 1.15 | 0.81 | | 0.55, 1.19 | 0.88 | | 0.61, 1.26 | |
| Yes | 5 | 100 | 7 | | 242 | 0.81 | | 0.32, 2.05 | 0.89 | | 0.34, 2.31 | 0.66 | | 0.37, 1.18 | |
| Missing | 15 |  | 18 | |  |  | |  |  | |  |  | |  | |
| Trend^i^ |  |  |  | |  | 0.83 | | 0.60, 1.13 | 0.86 | | 0.62, 1.18 | 0.84 | | 0.64, 1.11 | |
| High-intensity radar waves (e.g., as radar operator, radio operator,  aviation electrician's mate) |  |  |  | |  |  | |  |  | |  |  | |  | |
| Not exposed | 117 | 3,026 | 158 | | 6,377 | 1.00 | | Referent | 1.00 | | Referent | 1.00 | | Referent | |
| No | 34 | 800 | 42 | | 1,423 | 1.29 | | 0.86, 1.93 | 1.40 | | 0.93, 2.12 | 1.43 | | 0.99, 2.07 | |
| Yes | < 5^h^ | 15 | < 5^h^ | | 92 | 1.46 | | 0.19, 11.13 | 1.52 | | 0.20, 11.62 | 2.01 | | 0.47, 8.55 | |
| Missing | 16 |  | 26 | |  |  | |  |  | |  |  | |  | |
| Trend^i^ |  |  |  | |  | 1.28 | | 0.87, 1.88 | 1.38 | | 0.94, 2.04 | 1.42 | | 1.00, 2.02 | |
| Food contaminated with smoke, oil, or other chemicals |  |  |  | |  |  | |  |  | |  |  | |  | |
| Not exposed | 130 | 3,291 | 181 | | 7,414 | 1.00 | | Referent | 1.00 | | Referent | 1.00 | | Referent | |
| No | 7 | 151 | 8 | | 230 | 1.44 | | 0.65, 3.16 | 1.43 | | 0.64, 3.19 | 1.09 | | 0.51, 2.31 | |
| Yes | < 5^h^ | 76 | 6 | | 310 | 0.61 | | 0.19, 1.96 | 0.54 | | 0.17, 1.77 | 0.13 | | 0.02, 1.04 | |
| Missing | 28 |  | 33 | |  |  | |  |  | |  |  | |  | |
| Trend^i^ |  |  |  | |  | 0.92 | | 0.59, 1.45 | 0.88 | | 0.56, 1.39 | 0.66 | | 0.36, 1.21 | |
| Local food other than food provided by the Armed Forces |  |  |  | |  |  | |  |  | |  |  | |  | |
| Not exposed | 85 | 1,881 | 108 | | 3,741 | 1.00 | | Referent | 1.00 | | Referent | 1.00 | | Referent | |
| No | 68 | 1,688 | 98 | | 4,141 | 0.89 | | 0.63, 1.25 | 0.88 | | 0.62, 1.24 | 0.73 | | 0.52, 1.01 | |
| Yes | 8 | 328 | 12 | | 627 | 0.66 | | 0.31, 1.43 | 0.66 | | 0.31, 1.43 | 0.46 | | 0.24, 0.89 | |
| Missing | 7 |  | 10 | |  |  | |  |  | |  |  | |  | |
| Trend^i^ |  |  |  | |  | 0.85 | | 0.65, 1.12 | 0.85 | | 0.65, 1.12 | 0.66 | | 0.50, 0.87 | |
| Bathing in or drinking of water contaminated with smoke, oil, dead  animals or any chemicals |  |  |  | |  |  | |  |  | |  |  | |  | |
| Not exposed | 131 | 3,186 | 174 | | 6,641 | 1.00 | | Referent | 1.00 | | Referent | 1.00 | | Referent | |
| No | 10 | 169 | 13 | | 411 | 1.27 | | 0.65, 2.49 | 1.24 | | 0.63, 2.43 | 1.51 | | 0.61, 3.75 | |
| Yes | < 5^h^ | 15 | < 5^h^ | | 250 | 0.30 | | 0.04, 2.20 | 0.29 | | 0.04, 2.17 | 0.54 | | 0.16, 1.86 | |
| Missing | 26 |  | 37 | |  |  | |  |  | |  |  | |  | |
| Trend^i^ |  |  |  | |  | 0.87 | | 0.52, 1.45 | 0.85 | | 0.51, 1.43 | 1.20 | | 0.74, 1.95 | |
| Heat cramps, heat exhaustion, heat stroke or other heat illness |  |  |  | |  |  | |  |  | |  |  | |  | |
| Not exposed | 129 | 3,162 | 172 | | 6,645 | 1.00 | | Referent | 1.00 | | Referent | 1.00 | | Referent | |
| No | 7 | 198 | 8 | | 292 | 1.57 | | 0.68, 3.61 | 1.62 | | 0.69, 3.78 | 1.24 | | 0.63, 2.47 | |
| Yes | 27 | 613 | 41 | | 1,751 | 1.06 | | 0.69, 1.65 | 1.07 | | 0.69, 1.66 | 0.82 | | 0.48, 1.41 | |
| Missing | 5 |  | 7 | |  |  | |  |  | |  |  | |  | |
| Trend^i^ |  |  |  | |  | 1.05 | | 0.85, 1.30 | 1.05 | | 0.85, 1.30 | 0.96 | | 0.75, 1.23 | |
| Heard chemical alarms sounding |  |  |  | |  |  | |  |  | |  |  | |  | |
| Not exposed | 154 | 3,722 | 206 | | 7,944 | 1.00 | | Referent | 1.00 | | Referent | 1.00 | | Referent | |
| No | 7 | 169 | 15 | | 810 | 0.60 | | 0.28, 1.30 | 0.69 | | 0.30, 1.57 | 0.46 | | 0.20, 1.05 | |
| Yes | 0 | 0 | 0 | | 0 | ^j^ | | ^j^ | ^j^ | | ^j^ | ^j^ | | ^j^ | |
| Missing | 7 |  | 7 | |  |  | |  |  | |  |  | |  | |
| Trend^i^ |  |  |  | |  | 0.60 | | 0.28, 1.30 | 0.69 | | 0.30, 1.57 | 0.75 | | 0.30, 1.88 | |
| Explosion in the air or on the ground within one mile of you (e.g.,  artillery, rockets, mortars) |  |  |  | |  |  | |  |  | |  |  | |  | |
| Not exposed | 49 | 1,349 | 71 | | 3,154 | 1.00 | | Referent | 1.00 | | Referent | 1.00 | | Referent | |
| No | 101 | 2,336 | 133 | | 4,920 | 1.28 | | 0.89, 1.84 | 1.24 | | 0.86, 1.79 | 1.24 | | 0.85, 1.80 | |
| Yes | 9 | 189 | 13 | | 521 | 2.57 | | 1.18, 5.57 | 2.48 | | 1.13, 5.46 | 2.53 | | 1.36, 4.71 | |
| Missing | 9 |  | 11 | |  |  | |  |  | |  |  | |  | |
| Trend^i^ |  |  |  | |  | 1.41 | | 1.03, 1.93 | 1.37 | | 0.99, 1.89 | 1.45 | | 1.03, 2.06 | |
| ***While you were in WWII, the Korean War, and/or the Vietnam War^g^: did you feel ill from exposure to*** |  |  |  | |  |  | |  |  | |  |  | |  | |
| Mixing and application of herbicides |  |  |  | |  |  | |  |  | |  |  | |  | |
| Not exposed | 150 | 3,661 | 201 | | 7,744 | 1.00 | | Referent | 1.00 | | Referent | 1.00 | | Referent | |
| No | < 5^h^ | 87 | < 5^h^ | | 87 | 2.41 | | 0.85, 6.87 | 2.03 | | 0.69, 6.02 | 2.06 | | 0.96, 4.42 | |
| Yes | 0 | 0 | 0 | | 0 | ^j^ | | ^j^ | ^j^ | | ^j^ | ^j^ | | ^j^ | |
| Missing | 10 |  | 11 | |  |  | |  |  | |  |  | |  | |
| Trend^i^ |  |  |  | |  | 2.41 | | 0.85, 6.87 | 2.03 | | 0.69, 6.02 | 1.55 | | 0.97, 2.48 | |
| Exposure to herbicides in the field |  |  |  | |  |  | |  |  | |  |  | |  | |
| Not exposed | 120 | 2,839 | 159 | | 5,982 | 1.00 | | Referent | 1.00 | | Referent | 1.00 | | Referent | |
| No | 10 | 304 | 10 | | 304 | 1.68 | | 0.85, 3.31 | 1.71 | | 0.85, 3.45 | 1.25 | | 0.87, 1.80 | |
| Yes | 0 | 0 | < 5^h^ | | 92 | ^j^ | | ^j^ | ^j^ | | ^j^ | ^j^ | | ^j^ | |
| Missing | 34 |  | 46 | |  |  | |  |  | |  |  | |  | |
| Trend^i^ |  |  |  | |  | 1.08 | | 0.60, 1.91 | 1.02 | | 0.57, 1.82 | 1.14 | | 0.80, 1.63 | |
|  |  |  |  | |  |  | |  |  | |  |  | |  | |
|  |  |  |  | |  |  | |  |  | |  |  | |  | |
|  |  |  |  | |  |  | |  |  | |  |  | |  | |
|  |  |  |  | |  |  | |  |  | |  |  | |  | |
|  |  |  |  | |  |  | |  |  | |  |  | |  | |
| Mixing and application of riot control substances |  |  |  | |  |  | |  |  | |  |  | |  | |
| Not exposed | 159 | 3,911 | 211 | | 8,084 | 1.00 | | Referent | 1.00 | | Referent | 1.00 | | Referent | |
| No | < 5^h^ | 9 | < 5^h^ | | 9 | 4.54 | | 1.01, 20.42 | 4.83 | | 1.08, 21.61 | 5.21 | | 2.88, 9.42 | |
| Yes | 0 | 0 | 0 | | 0 | ^j^ | | ^j^ | ^j^ | | ^j^ | ^j^ | | ^j^ | |
| Missing | 3 |  | 3 | |  |  | |  |  | |  |  | |  | |
| Trend^i^ |  |  |  | |  | 4.54 | | 1.01, 20.42 | 4.83 | | 1.08, 21.61 | 3.66 | | 1.80, 7.43 | |
| Exposure to riot control substances in the field |  |  |  | |  |  | |  |  | |  |  | |  | |
| Not exposed | 152 | 3,654 | 200 | | 7,537 | 1.00 | | Referent | 1.00 | | Referent | 1.00 | | Referent | |
| No | 6 | 165 | 8 | | 325 | 1.09 | | 0.47, 2.53 | 1.13 | | 0.49, 2.64 | 0.68 | | 0.36, 1.26 | |
| Yes | < 5^h^ | 102 | < 5^h^ | | 165 | 0.75 | | 0.22, 2.58 | 0.78 | | 0.23, 2.66 | 0.42 | | 0.14, 1.29 | |
| Missing | 3 |  | 4 | |  |  | |  |  | |  |  | |  | |
| Trend^i^ |  |  |  | |  | 0.93 | | 0.57, 1.52 | 0.96 | | 0.59, 1.56 | 0.73 | | 0.41, 1.31 | |
| Mixing and application of burning agents |  |  |  | |  |  | |  |  | |  |  | |  | |
| Not exposed | 145 | 3,601 | 195 | | 7,594 | 1.00 | | Referent | 1.00 | | Referent | 1.00 | | Referent | |
| No | 10 | 173 | 11 | | 263 | 1.97 | | 0.98, 3.96 | 2.21 | | 1.10, 4.45 | 1.53 | | 1.07, 2.18 | |
| Yes | < 5^h^ | 20 | < 5^h^ | | 20 | 3.65 | | 1.09, 12.27 | 3.35 | | 1.00, 11.26 | 2.53 | | 0.87, 7.37 | |
| Missing | 6 |  | 7 | |  |  | |  |  | |  |  | |  | |
| Trend^i^ |  |  |  | |  | 1.94 | | 1.21, 3.10 | 1.97 | | 1.24, 3.13 | 2.00 | | 1.15, 3.49 | |
| Exposure to burning agents in the field |  |  |  | |  |  | |  |  | |  |  | |  | |
| Not exposed | 134 | 3,286 | 175 | | 6,610 | 1.00 | | Referent | 1.00 | | Referent | 1.00 | | Referent | |
| No | 19 | 451 | 24 | | 842 | 0.95 | | 0.57, 1.58 | 0.99 | | 0.59, 1.64 | 0.73 | | 0.47, 1.13 | |
| Yes | < 5^h^ | 61 | 6 | | 275 | 0.63 | | 0.20, 2.03 | 0.66 | | 0.21, 2.12 | 0.08 | | 0.01, 0.62 | |
| Missing | 8 |  | 11 | |  |  | |  |  | |  |  | |  | |
| Trend^i^ |  |  |  | |  | 0.88 | | 0.60, 1.29 | 0.90 | | 0.61, 1.33 | 0.61 | | 0.39, 0.95 | |
| ***While you were in the Korean War, the Vietnam War, and/or the Gulf War^g^: did you feel ill from exposure to*** |  |  |  | |  |  | |  |  | |  |  | |  | |
| Microwave radiation |  |  |  | |  |  | |  |  | |  |  | |  | |
| Not exposed | 108 | 2,792 | 151 | | 6,261 | 1.00 | | Referent | 1.00 | | Referent | 1.00 | | Referent | |
| No | 9 | 227 | 12 | | 475 | 1.21 | | 0.59, 2.47 | 1.20 | | 0.58, 2.47 | 1.15 | | 0.71, 1.89 | |
| Yes | 0 | 0 | < 5^h^ | | 91 | ^j^ | | ^j^ | ^j^ | | ^j^ | ^j^ | | ^j^ | |
| Missing | 24 |  | 30 | |  |  | |  |  | |  |  | |  | |
| Trend^i^ |  |  |  | |  | 0.97 | | 0.51, 1.84 | 0.95 | | 0.50, 1.82 | 1.02 | | 0.63, 1.67 | |
|  |  |  |  | |  |  | |  |  | |  |  | |  | |
|  |  |  |  | |  |  | |  |  | |  |  | |  | |
|  |  |  |  | |  |  | |  |  | |  |  | |  | |
|  |  |  |  | |  |  | |  |  | |  |  | |  | |
|  |  |  |  | |  |  | |  |  | |  |  | |  | |
| ***While you were in the Vietnam War^g^: did you feel ill from exposure to*** |  |  |  | |  |  | |  |  | |  |  | |  | |
| Mixing and application of Agent Orange |  |  |  | |  |  | |  |  | |  |  | |  | |
| Not exposed | 91 | 2,145 | 128 | | 5,050 | 1.00 | | Referent | 1.00 | | Referent | 1.00 | | Referent | |
| No | 5 | 87 | 7 | | 242 | 1.01 | | 0.40, 2.54 | NA | | NA | 0.61 | | 0.29, 1.29 | |
| Yes | 0 | 0 | 0 | | 0 | ^j^ | | ^j^ | NA | | NA | ^j^ | | ^j^ | |
| Missing | 8 |  | 10 | |  |  | |  |  | |  |  | |  | |
| Trend^i^ |  |  |  | |  | 1.01 | | 0.40, 2.54 | NA | | NA | 0.60 | | 0.32, 1.13 | |
| Exposure to Agent Orange in the field |  |  |  | |  |  | |  |  | |  |  | |  | |
| Not exposed | 40 | 841 | 51 | | 1,729 | 1.00 | | Referent | 1.00 | | Referent | 1.00 | | Referent | |
| No | 31 | 636 | 49 | | 2,045 | 0.77 | | 0.47, 1.27 | NA | | NA | 0.82 | | 0.51, 1.33 | |
| Yes | 5 | 59 | 9 | | 352 | 0.85 | | 0.32, 2.26 | NA | | NA | 0.62 | | 0.17, 2.23 | |
| Missing | 28 |  | 36 | |  |  | |  |  | |  |  | |  | |
| Trend^i^ |  |  |  | |  | 0.84 | | 0.56, 1.25 | NA | | NA | 0.72 | | 0.46, 1.12 | |
| ***While you were in the Gulf War^g^: did you feel ill from exposure to*** |  |  |  | |  |  | |  |  | |  |  | |  | |
| Use of depleted uranium (DU) for munitions or armor |  |  |  | |  |  | |  |  | |  |  | |  | |
| Not exposed | 5 | 213 | 8 | | 495 | 1.00 | | Referent | 1.00 | | Referent | 1.00 | | Referent | |
| No | < 5^h^ | 43 | 5 | | 389 | 0.35 | | 0.04, 3.39 | NA | | NA | 0.00 | | 0.00, 0.88 | |
| Yes | 0 | 0 | 0 | | 0 | ^j^ | | ^j^ | NA | | NA | ^j^ | | ^j^ | |
| Missing | 2 |  | 3 | |  |  | |  |  | |  |  | |  | |
| Trend^i^ |  |  |  | |  | 0.35 | | 0.04, 3.39 | NA | | NA | 0.62 | | 0.15, 2.52 | |
| CARC (Chemical Agent Resistant Compound) paint |  |  |  | |  |  | |  |  | |  |  | |  | |
| Not exposed | 5 | 205 | 10 | | 663 | 1.00 | | Referent | 1.00 | | Referent | 1.00 | | Referent | |
| No | < 5^h^ | 27 | < 5^h^ | | 197 | 0.63 | | 0.04, 9.88 | NA | | NA | ^j^ | | ^j^ | |
| Yes | 0 | 0 | 0 | | 0 | ^j^ | | ^j^ | NA | | NA | ^j^ | | ^j^ | |
| Missing | 2 |  | 3 | |  |  | |  |  | |  |  | |  | |
| Trend^i^ |  |  |  | |  | 0.63 | | 0.04, 9.88 | NA | | NA | ^j^ | | ^j^ | |
| Scud missile explosion in the air or on the ground within one mile of  you |  |  |  | |  |  | |  |  | |  |  | |  | |
| Not exposed | < 5^h^ | 153 | 9 | | 593 | 1.00 | | Referent | 1.00 | | Referent | 1.00 | | Referent | |
| No | < 5^h^ | 78 | < 5^h^ | | 172 | 36.01 | | 0.66, 1973.21 | NA | | NA | 6.69 | | 1.09, 41.13 | |
| Yes | 0 | 0 | < 5^h^ | | 94 | ^j^ | | ^j^ | NA | | NA | ^j^ | | ^j^ | |
| Missing | 2 |  | 3 | |  |  | |  |  | |  |  | |  | |
| Trend^i^ |  |  |  | |  | 2.78 | | 0.40, 19.22 | NA | | NA | 2.58 | | 0.75, 8.93 | |
|  |  |  |  | |  |  | |  |  | |  |  | |  | |
|  |  |  |  | |  |  | |  |  | |  |  | |  | |
|  |  |  |  | |  |  | |  |  | |  |  | |  | |
|  |  |  |  | |  |  | |  |  | |  |  | |  | |
| Smoke from oil well fires |  |  |  | |  |  | |  |  | |  |  | |  | |
| Not exposed | 5 | 206 | 5 | | 206 | 1.00 | | Referent | 1.00 | | Referent | 1.00 | | Referent | |
| No | 0 | 0 | < 5^h^ | | 232 | ^j^ | | ^j^ | NA | | NA | ^j^ | | ^j^ | |
| Yes | < 5^h^ | 27 | 5 | | 391 | ^j^ | | ^j^ | NA | | NA | ^j^ | | ^j^ | |
| Missing | 2 |  | 3 | |  |  | |  |  | |  |  | |  | |
| Trend^i^ |  |  |  | |  | 0.17 | | 0.02, 1.39 | NA | | NA | ^j^ | | ^j^ | |
| Exposure to nerve gas (e.g., during munitions destruction) |  |  |  | |  |  | |  |  | |  |  | |  | |
| Not exposed | 7 | 246 | 11 | | 593 | 1.00 | | Referent | 1.00 | | Referent | 1.00 | | Referent | |
| No | 0 | 0 | < 5^h^ | | 94 | ^j^ | | ^j^ | NA | | NA | ^j^ | | ^j^ | |
| Yes | 0 | 0 | < 5^h^ | | 187 | ^j^ | | ^j^ | NA | | NA | ^j^ | | ^j^ | |
| Missing | 1 |  | 2 | |  |  | |  |  | |  |  | |  | |
| Trend^i^ |  |  |  | |  | ^j^ | | ^j^ | NA | | NA | ^j^ | | ^j^ | |
| High levels of dust/sand |  |  |  | |  |  | |  |  | |  |  | |  | |
| Not exposed | < 5^h^ | 86 | < 5^h^ | | 86 | 1.00 | | Referent | 1.00 | | Referent | 1.00 | | Referent | |
| No | < 5^h^ | 165 | 12 | | 857 | ^j^ | | ^j^ | NA | | NA | ^j^ | | ^j^ | |
| Yes | 0 | 0 | 0 | | 0 | ^j^ | | ^j^ | NA | | NA | ^j^ | | ^j^ | |
| Missing | 1 |  | 1 | |  |  | |  |  | |  |  | |  | |
| Trend^i^ |  |  |  | |  | ^j^ | | ^j^ | NA | | NA | ^j^ | | ^j^ | |
| Ground level fumigation |  |  |  | |  |  | |  |  | |  |  | |  | |
| Not exposed | 6 | 204 | 9 | | 468 | 1.00 | | Referent | 1.00 | | Referent | 1.00 | | Referent | |
| No | 0 | 0 | < 5^h^ | | 282 | ^j^ | | ^j^ | NA | | NA | ^j^ | | ^j^ | |
| Yes | 0 | 0 | < 5^h^ | | 83 | ^j^ | | ^j^ | NA | | NA | ^j^ | | ^j^ | |
| Missing | 2 |  | 3 | |  |  | |  |  | |  |  | |  | |
| Trend^i^ |  |  |  | |  | ^j^ | | ^j^ | NA | | NA | ^j^ | | ^j^ | |

Abbreviations: ALS, amyotrophic lateral sclerosis; ALSFRS-R, ALS Functional Rating Scale-Revised; CARC, Chemical Agent Resistant Compound; CI, confidence interval; DU, depleted uranium; GENEVA, Genes and Environmental Exposures in Veterans with Amyotrophic Lateral Sclerosis; Gulf, 1990-1991 Persian Gulf; HR, hazard ratio; IP, inverse probability; NA, not applicable; PM, person-months; VA, Department of Veterans Affairs; WWII, World War II.

^a^ Fourteen ALS cases were excluded from this analysis because (1) they were missing data on diagnosis date (n = 7) or (2) they died before GENEVA enrollment (i.e., enrollment was completed by proxy after the case died; n = 7).

^b^ Adjusted for age (modeled with indicator variables corresponding to 5-year groups or, for exposures queried only in reference to deployment to the Gulf War, modeled with a linear term that was centered at age 60—the median age among all cases).

^c^ Adjusted for age (modeled with indicator variables corresponding to 5-year groups), war/operation of longest deployment, and the pairwise interaction between war/operation of longest deployment and the natural logarithm of time since diagnosis.

^d^ Weighted for confounding (conditional on age [modeled with indicator variables corresponding to 5-year groups or, for exposures queried only in reference to deployment to the Gulf War, modeled with a linear term that was centered at age 60—the median age among all cases] and war/operation of longest deployment), not missing baseline ALSFRS-R score (conditional on most recent ALS diagnosis category, symptom onset site, diagnostic delay [months; modeled with the natural logarithm of a linear term], and time from diagnosis to enrollment in the Registry [months; modeled with a restricted, quadratic spline with knots at 7.72, 13.24, 23.06, and 44.19 months based on percentiles of the distribution in the Registry cases not missing baseline ALSFRS-R score]), and participating in GENEVA (conditional on race/ethnicity, being a current patient of a VA Medical Center, most recent ALS diagnosis category, symptom onset site, diagnostic delay [months; modeled with linear, quadratic, and cubic terms], time from diagnosis to enrollment in the Registry [months; modeled with a linear term], and baseline ALSFRS-R score [modeled with a restricted, quadratic spline with knots at 12, 34, and 44 based on percentiles of the distribution in GENEVA cases]). 95% CIs were calculated with robust variance estimates.

^e^ Person-months calculated for time on study (i.e., the difference between the GENEVA enrollment date and the death date or July 25, 2013).

^f^ HRs and 95% CIs correspond to time since diagnosis accounting for late entry into the risk set at the GENEVA enrollment date.

^g^ The GENEVA study questionnaire asked "Were you deployed to..." the following wars where each war was asked about with a separate question: World War II (defined as the period from December 7, 1941, to December 31, 1946), the Korean War (defined as the period from June 27, 1950, to January 31, 1955), the Vietnam War (defined as the period from August 3, 1964, to May 7, 1975), and the Persian Gulf War (defined as the period from August 2, 1990, to December 31, 1991) [[1](#_ENREF_1)].

^h^ Suppressed to preserve the confidentiality of study participants.

^i^ Used ordinal scores (0, 1, 2). Reference category included for linear trend test.

^j^ Unable to estimate HR and 95% CI.

Table D. Military deployments to the Vietnam War^a^ and amyotrophic lateral sclerosis survival in GENEVA^b^, United States of America, 2005-2013.

|  | Deaths | | Total | | Adjusted^c^ | | IP-weighted^d^ | |
| --- | --- | --- | --- | --- | --- | --- | --- | --- |
| Variable | No. | PM^e^ | No. | PM^e^ | HR^f^ | 95% CI^f^ | HR^f^ | 95% CI^f^ |
| Total number of theaters of operation for the Vietnam War |  |  |  |  |  |  |  |  |
| Not deployed to any war/operation^g^ | 250 | 5,790 | 348 | 13,683 | 1.00 | Referent | 1.00 | Referent |
| Not deployed to the Vietnam War | 75 | 1,980 | 102 | 4,247 | 0.95 | 0.71, 1.28 | 0.68 | 0.43, 1.07 |
| 1 (Median = 1) | 76 | 1,792 | 101 | 3,754 | 1.13 | 0.86, 1.49 | 1.18 | 0.86, 1.62 |
| 2 (2) | 16 | 374 | 31 | 1,579 | 0.58 | 0.35, 0.98 | 0.71 | 0.36, 1.39 |
| > 2 (3) | 11 | 276 | 12 | 355 | 1.58 | 0.85, 2.92 | 1.31 | 0.89, 1.91 |
| Missing | 18 |  | 22 |  |  |  |  |  |
| Trend^h^ |  |  |  |  | 0.92 | 0.66, 1.28 | 1.03 | 0.75, 1.42 |
| Total length (years) of deployment to all theaters of operation for the Vietnam War |  |  |  |  |  |  |  |  |
| Not deployed to any war/operation^g^ | 250 | 5,790 | 348 | 13,683 | 1.00 | Referent | 1.00 | Referent |
| Not deployed to the Vietnam War | 75 | 1,980 | 102 | 4,247 | 0.96 | 0.71, 1.28 | 0.68 | 0.43, 1.07 |
| ≤ 1 (0.91) | 56 | 1,268 | 75 | 2,809 | 1.14 | 0.84, 1.56 | 1.27 | 0.95, 1.70 |
| > 1-2 (1.11) | 29 | 800 | 44 | 1,932 | 0.82 | 0.55, 1.21 | 0.69 | 0.46, 1.03 |
| > 2-3 (2.12) | 11 | 236 | 14 | 501 | 1.50 | 0.81, 2.79 | 1.33 | 0.90, 1.96 |
| > 3 (5.90) | 5 | 114 | 8 | 331 | 0.65 | 0.27, 1.59 | 0.48 | 0.10, 2.25 |
| Missing | 20 |  | 25 |  |  |  |  |  |
| Trend^h^ |  |  |  |  | 0.94 | 0.79, 1.12 | 1.01 | 0.84, 1.22 |
| End of most recent period of deployment to the Vietnam War (year) |  |  |  |  |  |  |  |  |
| Not deployed to any war/operation^g^ | 250 | 5,790 | 348 | 13,683 | 1.00 | Referent | 1.00 | Referent |
| Not deployed to the Vietnam War | 75 | 1,980 | 102 | 4,247 | 0.95 | 0.71, 1.28 | 0.68 | 0.43, 1.06 |
| ≤ 1965 (1964.0) | 8 | 214 | 9 | 307 | 1.07 | 0.52, 2.18 | 1.74 | 0.77, 3.95 |
| 1966-1967 (1967.0) | 21 | 453 | 26 | 811 | 1.23 | 0.76, 1.99 | 1.18 | 0.77, 1.81 |
| 1968-1969 (1969.0) | 30 | 665 | 49 | 2,147 | 0.77 | 0.52, 1.14 | 0.56 | 0.26, 1.23 |
| 1970-1971 (1970.0) | 31 | 778 | 41 | 1,587 | 1.14 | 0.77, 1.68 | 1.47 | 1.09, 1.99 |
| 1972-1973 (1972.0) | 6 | 177 | 10 | 498 | 0.96 | 0.42, 2.18 | 0.87 | 0.39, 1.94 |
| > 1973 (1974.5) | 5 | 132 | 6 | 224 | 1.62 | 0.65, 4.03 | 1.37 | 0.55, 3.37 |
| Missing | 20 |  | 25 |  |  |  |  |  |
| Trend^h^ |  |  |  |  | 1.00 | 0.89, 1.12 | 1.13 | 0.97, 1.32 |

Abbreviation: ALS, amyotrophic lateral sclerosis; ALSFRS-R, ALS Functional Rating Scale-Revised; CI, confidence interval; GENEVA, Genes and Environmental Exposures in Veterans with Amyotrophic Lateral Sclerosis; HR, hazard ratio; IP, inverse probability; PM, person-months; VA, Department of Veterans Affairs.

^a^ The GENEVA study questionnaire asked "Were you deployed to..." the Vietnam War (defined as the period from August 3, 1964, to May 7, 1975) [[1](#_ENREF_1)].

^b^ Fourteen ALS cases were excluded from this analysis because (1) they were missing data on diagnosis date (n = 7) or (2) they died before GENEVA enrollment (i.e., enrollment was completed by proxy after the case died; n = 7).

^c^ Adjusted for age (modeled with indicator variables corresponding to 5-year groups).

^d^ Weighted for confounding (conditional on age [modeled with indicator variables corresponding to 5-year groups]), not missing baseline ALSFRS-R score (conditional on most recent ALS diagnosis category, symptom onset site, diagnostic delay [months; modeled with the natural logarithm of a linear term], and time from diagnosis to enrollment in the Registry [months; modeled with a restricted, quadratic spline with knots at 7.72, 13.24, 23.06, and 44.19 months based on percentiles of the distribution in the Registry cases not missing baseline ALSFRS-R score]), and participating in GENEVA (conditional on race/ethnicity, being a current patient of a VA Medical Center, most recent ALS diagnosis category, symptom onset site, diagnostic delay [months; modeled with linear, quadratic, and cubic terms], time from diagnosis to enrollment in the Registry [months; modeled with a linear term], and baseline ALSFRS-R score [modeled with a restricted, quadratic spline with knots at 12, 34, and 44 based on percentiles of the distribution in GENEVA cases]). 95% CIs were calculated with robust variance estimates.

^e^ Person-months calculated for time on study (i.e., the difference between the GENEVA enrollment date and the death date or July 25, 2013).

^f^ HRs and 95% CIs correspond to time since diagnosis accounting for late entry into the risk set at the GENEVA enrollment date.

^g^ The GENEVA study questionnaire asked "Were you deployed to..." the following wars where each war was asked about with a separate question: World War II (defined as the period from December 7, 1941, to December 31, 1946), the Korean War (defined as the period from June 27, 1950, to January 31, 1955), the Vietnam War (defined as the period from August 3, 1964, to May 7, 1975), and the Persian Gulf War (defined as the period from August 2, 1990, to December 31, 1991) [[1](#_ENREF_1)]. The questionnaire also asked "Ever deployed..." to the following countries where each country was asked about with a separate question: Grenada, Lebanon, Panama, Somalia, Bosnia, Kosovo, Rwanda, Afghanistan, and Iraq/Persian Gulf region (Gulf War II) [[1](#_ENREF_1)].

^h^ Used within-category medians that were calculated using all cases. Individuals in the categories labeled "Not deployed to any war/operation" and "Not deployed to the Vietnam War" were excluded for the linear trend test.

Table E. Military exposures during deployment to the Vietnam War^a^ and amyotrophic lateral sclerosis survival in GENEVA^b^, United States of America, 2005-2013.

|  | Deaths | | Total | | Adjusted^c^ | | IP-weighted^d^ | |
| --- | --- | --- | --- | --- | --- | --- | --- | --- |
| Did you have direct contact with/were you exposed to | No. | PM^e^ | No. | PM^e^ | HR^f^ | 95% CI^f^ | HR^f^ | 95% CI^f^ |
| Mixing and application of Agent Orange^g^ |  |  |  |  |  |  |  |  |
| No | 91 | 2,145 | 128 | 5,050 | 1.00 | Referent | 1.00 | Referent |
| Yes | 6 | 127 | 8 | 283 | 1.11 | 0.47, 2.61 | 0.62 | 0.32, 1.20 |
| Missing | 7 |  | 9 |  |  |  |  |  |
| Exposure to Agent Orange in the field^g^ |  |  |  |  |  |  |  |  |
| No | 40 | 841 | 51 | 1,729 | 1.00 | Referent | 1.00 | Referent |
| Yes | 39 | 795 | 63 | 2,668 | 0.73 | 0.46, 1.17 | 0.66 | 0.42, 1.05 |
| Missing | 25 |  | 31 |  |  |  |  |  |
| Mixing and application of other herbicides |  |  |  |  |  |  |  |  |
| No | 96 | 2,336 | 136 | 5,494 | 1.00 | Referent | 1.00 | Referent |
| Yes | < 5^h^ | 29 | < 5^h^ | 29 | 3.19 | 0.73, 13.81 | 1.92 | 1.15, 3.19 |
| Missing | 5 |  | 6 |  |  |  |  |  |
| Exposure to other herbicides in the field |  |  |  |  |  |  |  |  |
| No | 67 | 1,588 | 96 | 3,898 | 1.00 | Referent | 1.00 | Referent |
| Yes | 7 | 159 | 7 | 159 | 1.89 | 0.85, 4.23 | 1.40 | 0.91, 2.17 |
| Missing | 30 |  | 42 |  |  |  |  |  |
| Ionizing radiation from nuclear weapon testing |  |  |  |  |  |  |  |  |
| No | 94 | 2,337 | 132 | 5,354 | 1.00 | Referent | 1.00 | Referent |
| Yes | < 5^h^ | 2 | < 5^h^ | 61 | 0.98 | 0.13, 7.24 | 0.99 | 0.10, 10.00 |
| Missing | 9 |  | 11 |  |  |  |  |  |
| Microwave radiation |  |  |  |  |  |  |  |  |
| No | 78 | 2,020 | 113 | 4,786 | 1.00 | Referent | 1.00 | Referent |
| Yes | 9 | 214 | 13 | 554 | 1.02 | 0.50, 2.09 | 1.04 | 0.62, 1.75 |
| Missing | 17 |  | 19 |  |  |  |  |  |
| Use of personal pesticides, like creams, sprays or flea collars |  |  |  |  |  |  |  |  |
| No | 61 | 1,471 | 88 | 3,653 | 1.00 | Referent | 1.00 | Referent |
| Yes | 40 | 984 | 53 | 1,981 | 1.18 | 0.78, 1.78 | 0.81 | 0.54, 1.23 |
| Missing | 3 |  | 4 |  |  |  |  |  |
| Use of pesticides on your clothing or bedding |  |  |  |  |  |  |  |  |
| No | 63 | 1,655 | 93 | 4,061 | 1.00 | Referent | 1.00 | Referent |
| Yes | 30 | 580 | 41 | 1,422 | 1.09 | 0.69, 1.73 | 0.81 | 0.49, 1.35 |
| Missing | 11 |  | 11 |  |  |  |  |  |
| Exhaust from heaters or generators (e.g., kerosene heaters, tent heaters) |  |  |  |  |  |  |  |  |
| No | 66 | 1,648 | 94 | 3,840 | 1.00 | Referent | 1.00 | Referent |
| Yes | 32 | 656 | 45 | 1,711 | 1.15 | 0.74, 1.80 | 0.88 | 0.54, 1.45 |
| Missing | 6 |  | 6 |  |  |  |  |  |
| Exposure to diesel and/or other petrochemical fumes |  |  |  |  |  |  |  |  |
| No | 30 | 795 | 39 | 1,535 | 1.00 | Referent | 1.00 | Referent |
| Yes | 72 | 1,631 | 103 | 4,042 | 0.89 | 0.57, 1.40 | 1.05 | 0.58, 1.93 |
| Missing | 2 |  | 3 |  |  |  |  |  |
| Burning trash or burning feces/manure |  |  |  |  |  |  |  |  |
| No | 52 | 1,206 | 75 | 3,016 | 1.00 | Referent | 1.00 | Referent |
| Yes | 49 | 1,238 | 66 | 2,609 | 1.17 | 0.78, 1.78 | 0.96 | 0.64, 1.44 |
| Missing | 3 |  | 4 |  |  |  |  |  |
| Exposure to paint, solvents, or petrochemical substances |  |  |  |  |  |  |  |  |
| No | 67 | 1,635 | 89 | 3,416 | 1.00 | Referent | 1.00 | Referent |
| Yes | 30 | 739 | 49 | 2,205 | 0.76 | 0.49, 1.18 | 0.77 | 0.49, 1.22 |
| Missing | 7 |  | 7 |  |  |  |  |  |
| High-intensity radar waves (e.g., as radar operator, radio operator, aviation electrician's mate) |  |  |  |  |  |  |  |  |
| No | 68 | 1,671 | 95 | 3,807 | 1.00 | Referent | 1.00 | Referent |
| Yes | 25 | 578 | 33 | 1,213 | 1.14 | 0.70, 1.86 | 1.29 | 0.81, 2.03 |
| Missing | 11 |  | 17 |  |  |  |  |  |
| Food contaminated with smoke, oil, or other chemicals |  |  |  |  |  |  |  |  |
| No | 79 | 1,973 | 113 | 4,637 | 1.00 | Referent | 1.00 | Referent |
| Yes | 9 | 168 | 13 | 480 | 0.92 | 0.44, 1.90 | 0.65 | 0.30, 1.42 |
| Missing | 16 |  | 19 |  |  |  |  |  |
| Local food other than food provided by the Armed Forces |  |  |  |  |  |  |  |  |
| No | 47 | 1,094 | 64 | 2,462 | 1.00 | Referent | 1.00 | Referent |
| Yes | 55 | 1,320 | 78 | 3,107 | 1.00 | 0.67, 1.49 | 0.77 | 0.52, 1.14 |
| Missing | 2 |  | 3 |  |  |  |  |  |
| Bathing in or drinking of water contaminated with smoke, oil, dead animals or any chemicals |  |  |  |  |  |  |  |  |
| No | 80 | 1,965 | 107 | 4,079 | 1.00 | Referent | 1.00 | Referent |
| Yes | 9 | 122 | 17 | 736 | 0.67 | 0.33, 1.37 | 0.58 | 0.22, 1.51 |
| Missing | 15 |  | 21 |  |  |  |  |  |
| Heat cramps, heat exhaustion, heat stroke or other heat illness |  |  |  |  |  |  |  |  |
| No | 72 | 1,696 | 100 | 3,915 | 1.00 | Referent | 1.00 | Referent |
| Yes | 30 | 718 | 42 | 1,657 | 1.15 | 0.74, 1.81 | 0.92 | 0.58, 1.47 |
| Missing | 2 |  | 3 |  |  |  |  |  |
| Heard chemical alarms sounding |  |  |  |  |  |  |  |  |
| No | 99 | 2,389 | 137 | 5,422 | 1.00 | Referent | 1.00 | Referent |
| Yes | < 5^h^ | 11 | 5 | 226 | 0.61 | 0.15, 2.54 | 0.85 | 0.28, 2.54 |
| Missing | 3 |  | 3 |  |  |  |  |  |
|  |  |  |  |  |  |  |  |  |
| Explosion in the air or on the ground within one mile of you (e.g., artillery, rockets, mortars) |  |  |  |  |  |  |  |  |
| No | 23 | 543 | 36 | 1,575 | 1.00 | Referent | 1.00 | Referent |
| Yes | 78 | 1,867 | 106 | 4,083 | 1.20 | 0.74, 1.94 | 1.09 | 0.67, 1.78 |
| Missing | 3 |  | 3 |  |  |  |  |  |
| Mixing and application of riot control substances |  |  |  |  |  |  |  |  |
| No | 102 | 2,466 | 143 | 5,713 | 1.00 | Referent | 1.00 | Referent |
| Yes | < 5^h^ | 6 | < 5^h^ | 6 | 4.93 | 0.58, 41.68 | 3.65 | 1.60, 8.33 |
| Missing | 1 |  | 1 |  |  |  |  |  |
| Exposure to riot control substances in the field |  |  |  |  |  |  |  |  |
| No | 95 | 2,223 | 132 | 5,181 | 1.00 | Referent | 1.00 | Referent |
| Yes | 8 | 249 | 11 | 472 | 0.88 | 0.41, 1.87 | 0.60 | 0.32, 1.11 |
| Missing | 1 |  | 2 |  |  |  |  |  |
| Mixing and application of burning agents |  |  |  |  |  |  |  |  |
| No | 89 | 2,168 | 128 | 5,236 | 1.00 | Referent | 1.00 | Referent |
| Yes | 11 | 177 | 12 | 268 | 2.13 | 1.08, 4.23 | 1.73 | 1.22, 2.45 |
| Missing | 4 |  | 5 |  |  |  |  |  |
| Exposure to burning agents in the field |  |  |  |  |  |  |  |  |
| No | 78 | 1,917 | 109 | 4,396 | 1.00 | Referent | 1.00 | Referent |
| Yes | 21 | 480 | 30 | 1,159 | 0.91 | 0.55, 1.52 | 0.66 | 0.38, 1.14 |
| Missing | 5 |  | 6 |  |  |  |  |  |
| Have you suffered a combat-related injury that required medical attention during your deployment? |  |  |  |  |  |  |  |  |
| No | 76 | 1,657 | 111 | 4,470 | 1.00 | Referent | 1.00 | Referent |
| Yes | 25 | 739 | 31 | 1,174 | 1.08 | 0.66, 1.77 | 0.83 | 0.44, 1.54 |
| Missing | 3 |  | 3 |  |  |  |  |  |

Abbreviation: ALS, amyotrophic lateral sclerosis; ALSFRS-R, ALS Functional Rating Scale-Revised; CI, confidence interval; GENEVA, Genes and Environmental Exposures in Veterans with Amyotrophic Lateral Sclerosis; HR, hazard ratio; IP, inverse probability; PM, person-months; VA, Department of Veterans Affairs.

^a^ The GENEVA study questionnaire asked "Were you deployed to..." the Vietnam War (defined as the period from August 3, 1964, to May 7, 1975) [[1](#_ENREF_1)].

^b^ Fourteen ALS cases were excluded from this analysis because (1) they were missing data on diagnosis date (n = 7) or (2) they died before GENEVA enrollment (i.e., enrollment was completed by proxy after the case died; n = 7).

^c^ Adjusted for age (modeled with indicator variables corresponding to 5-year groups).

^d^ Weighted for confounding (conditional on age [modeled with indicator variables corresponding to 5-year groups]), not missing baseline ALSFRS-R score (conditional on most recent ALS diagnosis category, symptom onset site, diagnostic delay [months; modeled with the natural logarithm of a linear term], and time from diagnosis to enrollment in the Registry [months; modeled with a restricted, quadratic spline with knots at 7.72, 13.24, 23.06, and 44.19 months based on percentiles of the distribution in the Registry cases not missing baseline ALSFRS-R score]), and participating in GENEVA (conditional on race/ethnicity, being a current patient of a VA Medical Center, most recent ALS diagnosis category, symptom onset site, diagnostic delay [months; modeled with linear, quadratic, and cubic terms], time from diagnosis to enrollment in the Registry [months; modeled with a linear term], and baseline ALSFRS-R score [modeled with a restricted, quadratic spline with knots at 12, 34, and 44 based on percentiles of the distribution in GENEVA cases]). 95% CIs were calculated with robust variance estimates.

^e^ Person-months calculated for time on study (i.e., the difference between the GENEVA enrollment date and the death date or July 25, 2013).

^f^ HRs and 95% CIs correspond to time since diagnosis accounting for late entry into the risk set at the GENEVA enrollment date.

^g^ Repeated from Table 5.

^h^ Suppressed to preserve the confidentiality of study participants.

Table F. Exposure-response for military exposures during deployment to the Vietnam War^a^ and amyotrophic lateral sclerosis survival in GENEVA^b^, United States of America, 2005-2013.

|  | Deaths | | Total | | Adjusted^c^ | | IP-weighted^d^ | |
| --- | --- | --- | --- | --- | --- | --- | --- | --- |
| Exposure | No. | PM^e^ | No. | PM^e^ | HR^f^ | 95% CI^f^ | HR^f^ | 95% CI^f^ |
| Total number of preventive vaccinations received by injection (shots) or by mouth while inside or outside the U.S. |  |  |  |  |  |  |  |  |
| 0 (Median = 0) | 23 | 656 | 33 | 1,470 | 1.00 | Referent | 1.00 | Referent |
| 1-10 (6) | 29 | 709 | 40 | 1,541 | 1.38 | 0.78, 2.45 | 1.43 | 0.87, 2.34 |
| 11-20 (14) | 15 | 363 | 17 | 516 | 1.82 | 0.90, 3.71 | 1.68 | 1.02, 2.75 |
| > 20 (68) | 14 | 249 | 22 | 872 | 1.19 | 0.60, 2.36 | 1.27 | 0.66, 2.43 |
| Missing | 23 |  | 33 |  |  |  |  |  |
| Trend (IQR = 15)^g^ |  |  |  |  | 1.00 | 0.88, 1.15 | 1.00 | 0.88, 1.13 |
| ***Number of days exposed to*** |  |  |  |  |  |  |  |  |
| Mixing and application of Agent Orange^h^ |  |  |  |  |  |  |  |  |
| Not exposed | 91 | 2,145 | 128 | 5,050 | 1.00 | Referent | 1.00 | Referent |
| ≤ 5 | < 5^i^ | 3 | < 5^i^ | 63 | 0.61 | 0.08, 4.50 | 0.22 | 0.01, 3.33 |
| 6-30 | < 5^i^ | 46 | < 5^i^ | 142 | 1.04 | 0.23, 4.70 | 0.80 | 0.14, 4.51 |
| > 30 | < 5^i^ | 59 | < 5^i^ | 59 | 1.31 | 0.31, 5.53 | 0.65 | 0.36, 1.16 |
| Missing | 8 |  | 10 |  |  |  |  |  |
| Trend^j^ |  |  |  |  | 1.10 | 0.61, 2.00 | 0.88 | 0.50, 1.55 |
| Exposure to Agent Orange in the field^h^ |  |  |  |  |  |  |  |  |
| Not exposed | 40 | 841 | 51 | 1,729 | 1.00 | Referent | 1.00 | Referent |
| ≤ 5 | 7 | 200 | 12 | 602 | 0.58 | 0.25, 1.32 | 0.60 | 0.30, 1.19 |
| 6-30 | 7 | 128 | 10 | 342 | 0.92 | 0.38, 2.22 | 0.81 | 0.28, 2.30 |
| > 30 | 22 | 408 | 35 | 1,442 | 0.82 | 0.47, 1.43 | 0.75 | 0.42, 1.32 |
| Missing | 28 |  | 37 |  |  |  |  |  |
| Trend^j^ |  |  |  |  | 0.96 | 0.76, 1.21 | 0.89 | 0.69, 1.15 |
| Mixing and application of other herbicides |  |  |  |  |  |  |  |  |
| Not exposed | 96 | 2,336 | 136 | 5,494 | 1.00 | Referent | 1.00 | Referent |
| ≤ 5 | < 5^i^ | 6 | < 5^i^ | 6 | ^k^ | ^k^ | 2.14 | 0.72, 6.34 |
| 6-30 | < 5^i^ | 16 | < 5^i^ | 16 | 2.22 | 0.29, 16.81 | 1.10 | 0.60, 2.02 |
| > 30 | < 5^i^ | 6 | < 5^i^ | 6 | 5.83 | 0.67, 51.05 | 4.11 | 1.67, 10.10 |
| Missing | 5 |  | 6 |  |  |  |  |  |
| Trend^j^ |  |  |  |  | 2.17 | 0.92, 5.14 | 1.90 | 1.15, 3.13 |
|  |  |  |  |  |  |  |  |  |
|  |  |  |  |  |  |  |  |  |
|  |  |  |  |  |  |  |  |  |
|  |  |  |  |  |  |  |  |  |
|  |  |  |  |  |  |  |  |  |
|  |  |  |  |  |  |  |  |  |
| Exposure to other herbicides in the field |  |  |  |  |  |  |  |  |
| Not exposed | 67 | 1,588 | 96 | 3,898 | 1.00 | Referent | 1.00 | Referent |
| ≤ 5 | < 5^i^ | 93 | < 5^i^ | 93 | 1.15 | 0.27, 4.87 | 1.23 | 0.66, 2.28 |
| 6-30 | < 5^i^ | 15 | < 5^i^ | 15 | 2.34 | 0.29, 18.67 | 1.53 | 0.78, 3.00 |
| > 30 | < 5^i^ | 32 | < 5^i^ | 32 | 2.51 | 0.73, 8.62 | 1.69 | 0.82, 3.50 |
| Missing | 31 |  | 43 |  |  |  |  |  |
| Trend^j^ |  |  |  |  | 1.51 | 0.90, 2.53 | 1.41 | 1.01, 1.98 |
| Ionizing radiation from nuclear weapon testing |  |  |  |  |  |  |  |  |
| Not exposed | 94 | 2,337 | 132 | 5,354 | 1.00 | Referent | 1.00 | Referent |
| ≤ 5 | < 5^i^ | 2 | < 5^i^ | 61 | 0.98 | 0.13, 7.24 | 0.99 | 0.10, 9.82 |
| 6-30 | 0 | 0 | 0 | 0 | ^k^ | ^k^ | ^k^ | ^k^ |
| > 30 | 0 | 0 | 0 | 0 | ^k^ | ^k^ | ^k^ | ^k^ |
| Missing | 9 |  | 11 |  |  |  |  |  |
| Trend^l^ |  |  |  |  | 0.99 | 0.51, 1.93 | 1.00 | 0.46, 2.18 |
| Microwave radiation |  |  |  |  |  |  |  |  |
| Not exposed or ≤ 5 | 79 | 2,038 | 115 | 4,895 | 1.00 | Referent | 1.00 | Referent |
| > 5 | 8 | 196 | 11 | 445 | 1.10 | 0.52, 2.32 | 1.17 | 0.64, 2.13 |
| Missing | 17 |  | 19 |  |  |  |  |  |
| Trend^j^ |  |  |  |  | 1.03 | 0.74, 1.45 | 0.96 | 0.75, 1.22 |
| Use of personal pesticides, like creams, sprays or flea collars |  |  |  |  |  |  |  |  |
| Not exposed | 61 | 1,471 | 88 | 3,653 | 1.00 | Referent | 1.00 | Referent |
| ≤ 30 | 9 | 159 | 11 | 312 | 1.49 | 0.70, 3.15 | 1.33 | 0.87, 2.03 |
| > 30 | 30 | 774 | 41 | 1,618 | 1.07 | 0.68, 1.69 | 0.64 | 0.38, 1.05 |
| Missing | 4 |  | 5 |  |  |  |  |  |
| Trend^j^ |  |  |  |  | 1.03 | 0.85, 1.24 | 0.84 | 0.68, 1.04 |
| Use of pesticides on your clothing or bedding |  |  |  |  |  |  |  |  |
| Not exposed | 63 | 1,655 | 93 | 4,061 | 1.00 | Referent | 1.00 | Referent |
| ≤ 30 | 7 | 147 | 9 | 293 | 1.19 | 0.51, 2.76 | 1.16 | 0.69, 1.93 |
| > 30 | 23 | 433 | 31 | 1,037 | 1.13 | 0.68, 1.86 | 0.72 | 0.38, 1.37 |
| Missing | 11 |  | 12 |  |  |  |  |  |
| Trend^j^ |  |  |  |  | 1.07 | 0.87, 1.32 | 0.89 | 0.68, 1.16 |
| Exhaust from heaters or generators (e.g., kerosene heaters, tent heaters) |  |  |  |  |  |  |  |  |
| Not exposed | 66 | 1,648 | 94 | 3,840 | 1.00 | Referent | 1.00 | Referent |
| ≤ 30 | 10 | 211 | 14 | 538 | 0.98 | 0.50, 1.93 | 0.66 | 0.25, 1.74 |
| > 30 | 21 | 414 | 29 | 1,047 | 1.27 | 0.75, 2.15 | 1.04 | 0.68, 1.58 |
| Missing | 7 |  | 8 |  |  |  |  |  |
| Trend^j^ |  |  |  |  | 1.11 | 0.89, 1.38 | 1.00 | 0.82, 1.22 |
|  |  |  |  |  |  |  |  |  |
| Exposure to diesel and/or other petrochemical fumes |  |  |  |  |  |  |  |  |
| Not exposed | 30 | 795 | 39 | 1,535 | 1.00 | Referent | 1.00 | Referent |
| ≤ 30 | 9 | 187 | 16 | 727 | 0.61 | 0.29, 1.32 | 0.62 | 0.27, 1.42 |
| > 30 | 62 | 1,425 | 86 | 3,296 | 0.95 | 0.60, 1.50 | 1.16 | 0.63, 2.12 |
| Missing | 3 |  | 4 |  |  |  |  |  |
| Trend^j^ |  |  |  |  | 1.03 | 0.85, 1.24 | 1.10 | 0.88, 1.39 |
| Burning trash or burning feces/manure |  |  |  |  |  |  |  |  |
| Not exposed | 52 | 1,206 | 75 | 3,016 | 1.00 | Referent | 1.00 | Referent |
| ≤ 5 | 9 | 219 | 9 | 219 | 1.67 | 0.79, 3.53 | 1.13 | 0.70, 1.83 |
| 6-30 | 8 | 171 | 10 | 298 | 1.14 | 0.53, 2.44 | 0.77 | 0.42, 1.42 |
| > 30 | 30 | 787 | 44 | 1,936 | 1.09 | 0.67, 1.77 | 0.99 | 0.61, 1.61 |
| Missing | 5 |  | 7 |  |  |  |  |  |
| Trend^j^ |  |  |  |  | 1.02 | 0.83, 1.24 | 1.00 | 0.81, 1.23 |
| Exposure to paint, solvents, or petrochemical substances |  |  |  |  |  |  |  |  |
| Not exposed | 67 | 1,635 | 89 | 3,416 | 1.00 | Referent | 1.00 | Referent |
| ≤ 30 | 12 | 232 | 18 | 704 | 0.89 | 0.47, 1.69 | 1.20 | 0.73, 1.97 |
| > 30 | 18 | 508 | 31 | 1,502 | 0.69 | 0.40, 1.18 | 0.56 | 0.30, 1.05 |
| Missing | 7 |  | 7 |  |  |  |  |  |
| Trend^j^ |  |  |  |  | 0.89 | 0.71, 1.11 | 0.82 | 0.64, 1.06 |
| High-intensity radar waves (e.g., as radar operator, radio operator, aviation electrician's mate) |  |  |  |  |  |  |  |  |
| Not exposed | 68 | 1,671 | 95 | 3,807 | 1.00 | Referent | 1.00 | Referent |
| ≤ 30 | 5 | 146 | 5 | 146 | 1.35 | 0.53, 3.45 | 1.50 | 0.82, 2.73 |
| > 30 | 19 | 425 | 27 | 1,060 | 1.09 | 0.63, 1.88 | 1.35 | 0.81, 2.25 |
| Missing | 12 |  | 18 |  |  |  |  |  |
| Trend^j^ |  |  |  |  | 1.04 | 0.83, 1.32 | 1.13 | 0.91, 1.41 |
| Food contaminated with smoke, oil, or other chemicals |  |  |  |  |  |  |  |  |
| Not exposed or ≤ 5 | 80 | 1,979 | 114 | 4,642 | 1.00 | Referent | 1.00 | Referent |
| > 5 | 8 | 163 | 12 | 475 | 0.81 | 0.38, 1.74 | 0.63 | 0.31, 1.28 |
| Missing | 16 |  | 19 |  |  |  |  |  |
| Trend^j^ |  |  |  |  | 0.99 | 0.67, 1.45 | 0.86 | 0.65, 1.15 |
| Local food other than food provided by the Armed Forces |  |  |  |  |  |  |  |  |
| Not exposed | 47 | 1,094 | 64 | 2,462 | 1.00 | Referent | 1.00 | Referent |
| ≤ 5 | 13 | 417 | 21 | 1,107 | 0.85 | 0.44, 1.63 | 0.80 | 0.49, 1.32 |
| 6-30 | 16 | 346 | 21 | 693 | 0.90 | 0.49, 1.63 | 0.62 | 0.35, 1.09 |
| > 30 | 25 | 546 | 35 | 1,295 | 1.18 | 0.71, 1.96 | 0.79 | 0.43, 1.46 |
| Missing | 3 |  | 4 |  |  |  |  |  |
| Trend^j^ |  |  |  |  | 1.08 | 0.87, 1.34 | 0.88 | 0.68, 1.14 |
|  |  |  |  |  |  |  |  |  |
| Bathing in or drinking of water contaminated with smoke, oil, dead animals or any chemicals |  |  |  |  |  |  |  |  |
| Not exposed or ≤ 30 | 84 | 2,031 | 114 | 4,369 | 1.00 | Referent | 1.00 | Referent |
| > 30 | 5 | 57 | 10 | 445 | 0.71 | 0.28, 1.82 | 0.66 | 0.23, 1.91 |
| Missing | 15 |  | 21 |  |  |  |  |  |
| Trend^j^ |  |  |  |  | 0.85 | 0.57, 1.27 | 0.84 | 0.51, 1.39 |
| Heat cramps, heat exhaustion, heat stroke or other heat illness |  |  |  |  |  |  |  |  |
| Not exposed | 72 | 1,696 | 100 | 3,915 | 1.00 | Referent | 1.00 | Referent |
| ≤ 5 | 11 | 241 | 15 | 581 | 1.31 | 0.68, 2.54 | 1.07 | 0.64, 1.77 |
| 6-30 | 8 | 224 | 15 | 765 | 0.63 | 0.30, 1.33 | 0.45 | 0.19, 1.05 |
| > 30 | 11 | 253 | 12 | 312 | 2.25 | 1.14, 4.43 | 1.97 | 1.16, 3.37 |
| Missing | 2 |  | 3 |  |  |  |  |  |
| Trend^j^ |  |  |  |  | 1.26 | 0.94, 1.70 | 1.13 | 0.82, 1.57 |
| Heard chemical alarms sounding |  |  |  |  |  |  |  |  |
| Not exposed | 99 | 2,389 | 137 | 5,422 | 1.00 | Referent | 1.00 | Referent |
| ≤ 5 | < 5^i^ | 3 | < 5^i^ | 140 | 0.56 | 0.08, 4.15 | 0.60 | 0.09, 3.98 |
| 6-30 | < 5^i^ | 8 | < 5^i^ | 8 | 2.47 | 0.32, 18.94 | 1.77 | 1.03, 3.06 |
| > 30 | 0 | 0 | < 5^i^ | 79 | ^k^ | ^k^ | ^k^ | ^k^ |
| Missing | 3 |  | 3 |  |  |  |  |  |
| Trend^j^ |  |  |  |  | 0.53 | 0.11, 2.52 | 1.30 | 0.52, 3.23 |
| Explosion in the air or on the ground within one mile of you (e.g., artillery, rockets, mortars) |  |  |  |  |  |  |  |  |
| Not exposed | 23 | 543 | 36 | 1,575 | 1.00 | Referent | 1.00 | Referent |
| ≤ 5 | 20 | 548 | 24 | 890 | 1.61 | 0.85, 3.06 | 1.58 | 0.90, 2.78 |
| 6-30 | 16 | 258 | 26 | 1,059 | 1.02 | 0.52, 2.00 | 1.26 | 0.68, 2.36 |
| > 30 | 40 | 1,014 | 53 | 1,993 | 1.15 | 0.67, 1.96 | 0.97 | 0.55, 1.70 |
| Missing | 5 |  | 6 |  |  |  |  |  |
| Trend^j^ |  |  |  |  | 0.99 | 0.81, 1.21 | 0.88 | 0.72, 1.08 |
| Mixing and application of riot control substances |  |  |  |  |  |  |  |  |
| Not exposed | 102 | 2,466 | 143 | 5,713 | 1.00 | Referent | 1.00 | Referent |
| ≤ 5 | < 5^i^ | 6 | < 5^i^ | 6 | 4.93 | 0.58, 41.68 | 3.68 | 1.59, 8.54 |
| 6-30 | 0 | 0 | 0 | 0 | ^k^ | ^k^ | ^k^ | ^k^ |
| > 30 | 0 | 0 | 0 | 0 | ^k^ | ^k^ | ^k^ | ^k^ |
| Missing | 1 |  | 1 |  |  |  |  |  |
| Trend^l^ |  |  |  |  | 1.70 | 0.84, 3.47 | 1.54 | 1.18, 2.03 |
| Exposure to riot control substances in the field |  |  |  |  |  |  |  |  |
| Not exposed or ≤ 5 | 97 | 2,279 | 135 | 5,301 | 1.00 | Referent | 1.00 | Referent |
| > 5 | 6 | 193 | 8 | 352 | 0.96 | 0.41, 2.25 | 0.88 | 0.41, 1.87 |
| Missing | 1 |  | 2 |  |  |  |  |  |
| Trend^j^ |  |  |  |  | 1.01 | 0.58, 1.77 | 0.57 | 0.33, 0.97 |
| Mixing and application of burning agents |  |  |  |  |  |  |  |  |
| Not exposed or ≤ 5 | 91 | 2,196 | 130 | 5,263 | 1.00 | Referent | 1.00 | Referent |
| > 5 | 9 | 149 | 10 | 240 | 1.96 | 0.93, 4.15 | 1.62 | 1.13, 2.32 |
| Missing | 4 |  | 5 |  |  |  |  |  |
| Trend^j^ |  |  |  |  | 1.35 | 0.88, 2.07 | 1.38 | 1.10, 1.75 |
| Exposure to burning agents in the field |  |  |  |  |  |  |  |  |
| Not exposed | 78 | 1,917 | 109 | 4,396 | 1.00 | Referent | 1.00 | Referent |
| ≤ 5 | 7 | 136 | 12 | 484 | 0.66 | 0.30, 1.45 | 0.37 | 0.14, 1.01 |
| > 5 | 11 | 319 | 15 | 649 | 0.96 | 0.49, 1.88 | 0.80 | 0.45, 1.40 |
| Missing | 8 |  | 9 |  |  |  |  |  |
| Trend^j^ |  |  |  |  | 0.84 | 0.55, 1.27 | 0.82 | 0.67, 1.00 |
| Have you suffered a combat-related injury that required medical attention during your  deployment? |  |  |  |  |  |  |  |  |
| Not exposed | 76 | 1,657 | 111 | 4,470 | 1.00 | Referent | 1.00 | Referent |
| ≤ 5 | 13 | 399 | 15 | 553 | 1.10 | 0.58, 2.09 | 0.99 | 0.62, 1.59 |
| 6-30 | 5 | 187 | 7 | 323 | 0.87 | 0.34, 2.25 | 1.06 | 0.53, 2.12 |
| > 30 | 7 | 153 | 9 | 297 | 1.26 | 0.55, 2.89 | 0.77 | 0.22, 2.70 |
| Missing | 3 |  | 3 |  |  |  |  |  |
| Trend^j^ |  |  |  |  | 1.08 | 0.76, 1.52 | 0.85 | 0.50, 1.44 |

Abbreviations: ALS, amyotrophic lateral sclerosis; ALSFRS-R, ALS Functional Rating Scale-Revised; CI, confidence interval; GENEVA, Genes and Environmental Exposures in Veterans with Amyotrophic Lateral Sclerosis; HR, hazard ratio; IP, inverse probability; IQR, interquartile range; PM, person-months; U.S., United States of America; VA, Department of Veterans Affairs.

^a^ The GENEVA study questionnaire asked "Were you deployed to..." the Vietnam War (defined as the period from August 3, 1964, to May 7, 1975) [[1](#_ENREF_1)].

^b^ Fourteen ALS cases were excluded from this analysis because (1) they were missing data on diagnosis date (n = 7) or (2) they died before GENEVA enrollment (i.e., enrollment was completed by proxy after the case died; n = 7).

^c^ Adjusted for age (modeled with indicator variables corresponding to 5-year groups).

^d^ Weighted for confounding (conditional on age [modeled with indicator variables corresponding to 5-year groups]), not missing baseline ALSFRS-R score (conditional on most recent ALS diagnosis category, symptom onset site, diagnostic delay [months; modeled with the natural logarithm of a linear term], and time from diagnosis to enrollment in the Registry [months; modeled with a restricted, quadratic spline with knots at 7.72, 13.24, 23.06, and 44.19 months based on percentiles of the distribution in the Registry cases not missing baseline ALSFRS-R score]), and participating in GENEVA (conditional on race/ethnicity, being a current patient of a VA Medical Center, most recent ALS diagnosis category, symptom onset site, diagnostic delay [months; modeled with linear, quadratic, and cubic terms], time from diagnosis to enrollment in the Registry [months; modeled with a linear term], and baseline ALSFRS-R score [modeled with a restricted, quadratic spline with knots at 12, 34, and 44 based on percentiles of the distribution in GENEVA cases]). 95% CIs were calculated with robust variance estimates.

^e^ Person-months calculated for time on study (i.e., the difference between the GENEVA enrollment date and the death date or July 25, 2013).

^f^ HRs and 95% CIs correspond to time since diagnosis accounting for late entry into the risk set at the GENEVA enrollment date.

^g^ Used within-category medians that were calculated using all cases. Scaled the HR to an IQR-unit increase in the exposure variable. IQRs were calculated using all cases except those in the reference category. Reference category included for linear trend test.

^h^ Repeated from Table B in S1 Supporting Information.

^i^ Suppressed to preserve the confidentiality of study participants.

^j^ Used category midpoints (0, 3, 18) or 50% above the lower bound of the highest category (46.5) and scaled the HR to a 20-day increase in the exposure variable. Reference category included for linear trend test.

^k^ Unable to estimate HR and 95% CI.

^l^ Used category midpoints (0, 3, 18) or 50% above the lower bound of the highest category (46.5) and scaled the HR to a one-day increase in the exposure variable. Reference category included for linear trend test.

Table G. Feel ill from military exposures during deployment to the Vietnam War^a^ and amyotrophic lateral sclerosis survival in GENEVA^b^, United States of America, 2005-2013.

|  | Deaths | | Total | | Adjusted^c^ | | IP-weighted^d^ | |
| --- | --- | --- | --- | --- | --- | --- | --- | --- |
| Did you feel ill from exposure to | No. | PM^e^ | No. | PM^e^ | HR^f^ | 95% CI^f^ | HR^f^ | 95% CI^f^ |
| Mixing and application of Agent Orange^g^ |  |  |  |  |  |  |  |  |
| Not exposed | 91 | 2,145 | 128 | 5,050 | 1.00 | Referent | 1.00 | Referent |
| No | 5 | 87 | 7 | 242 | 1.01 | 0.40, 2.54 | 0.61 | 0.29, 1.29 |
| Yes | 0 | 0 | 0 | 0 | ^h^ | ^h^ | ^h^ | ^h^ |
| Missing | 8 |  | 10 |  |  |  |  |  |
| Trend^i^ |  |  |  |  | 1.01 | 0.40, 2.54 | 0.60 | 0.32, 1.13 |
| Exposure to Agent Orange in the field^g^ |  |  |  |  |  |  |  |  |
| Not exposed | 40 | 841 | 51 | 1,729 | 1.00 | Referent | 1.00 | Referent |
| No | 31 | 636 | 49 | 2,045 | 0.77 | 0.47, 1.27 | 0.82 | 0.51, 1.33 |
| Yes | 5 | 59 | 9 | 352 | 0.85 | 0.32, 2.26 | 0.62 | 0.17, 2.23 |
| Missing | 28 |  | 36 |  |  |  |  |  |
| Trend^i^ |  |  |  |  | 0.84 | 0.56, 1.25 | 0.72 | 0.46, 1.12 |
| Mixing and application of other herbicides |  |  |  |  |  |  |  |  |
| Not exposed | 96 | 2,336 | 136 | 5,494 | 1.00 | Referent | 1.00 | Referent |
| No | < 5^j^ | 29 | < 5^j^ | 29 | 3.19 | 0.73, 13.81 | 1.00 | 0.55, 1.83 |
| Yes | 0 | 0 | 0 | 0 | ^h^ | ^h^ | ^h^ | ^h^ |
| Missing | 5 |  | 6 |  |  |  |  |  |
| Trend^i^ |  |  |  |  | 3.19 | 0.73, 13.81 | 2.47 | 1.43, 4.24 |
| Exposure to other herbicides in the field |  |  |  |  |  |  |  |  |
| Not exposed | 67 | 1,588 | 96 | 3,898 | 1.00 | Referent | 1.00 | Referent |
| No | 7 | 159 | 7 | 159 | 1.89 | 0.85, 4.23 | 1.45 | 0.99, 2.14 |
| Yes | 0 | 0 | 0 | 0 | ^h^ | ^h^ | ^h^ | ^h^ |
| Missing | 30 |  | 42 |  |  |  |  |  |
| Trend^i^ |  |  |  |  | 1.89 | 0.85, 4.23 | 1.39 | 0.89, 2.17 |
| Ionizing radiation from nuclear weapon testing |  |  |  |  |  |  |  |  |
| Not exposed | 94 | 2,337 | 132 | 5,354 | 1.00 | Referent | 1.00 | Referent |
| No | < 5^j^ | 2 | < 5^j^ | 61 | 0.98 | 0.13, 7.24 | 0.99 | 0.10, 9.82 |
| Yes | 0 | 0 | 0 | 0 | ^h^ | ^h^ | ^h^ | ^h^ |
| Missing | 9 |  | 11 |  |  |  |  |  |
| Trend^i^ |  |  |  |  | 0.98 | 0.13, 7.24 | 0.99 | 0.10, 10.31 |
| Microwave radiation |  |  |  |  |  |  |  |  |
| Not exposed | 78 | 2,020 | 113 | 4,786 | 1.00 | Referent | 1.00 | Referent |
| No | 8 | 201 | 11 | 449 | 1.06 | 0.50, 2.28 | 1.18 | 0.71, 1.96 |
| Yes | 0 | 0 | < 5^j^ | 91 | ^h^ | ^h^ | ^h^ | ^h^ |
| Missing | 18 |  | 20 |  |  |  |  |  |
| Trend^i^ |  |  |  |  | 0.87 | 0.43, 1.74 | 0.98 | 0.60, 1.61 |
| Use of personal pesticides, like creams, sprays or flea collars |  |  |  |  |  |  |  |  |
| Not exposed | 61 | 1,471 | 88 | 3,653 | 1.00 | Referent | 1.00 | Referent |
| No | 35 | 920 | 43 | 1,522 | 1.28 | 0.83, 1.98 | 0.94 | 0.62, 1.42 |
| Yes | < 5^j^ | 22 | < 5^j^ | 87 | 0.83 | 0.20, 3.43 | 0.21 | 0.03, 1.68 |
| Missing | 6 |  | 11 |  |  |  |  |  |
| Trend^i^ |  |  |  |  | 1.15 | 0.80, 1.67 | 0.84 | 0.57, 1.24 |
| Use of pesticides on your clothing or bedding |  |  |  |  |  |  |  |  |
| Not exposed | 63 | 1,655 | 93 | 4,061 | 1.00 | Referent | 1.00 | Referent |
| No | 24 | 445 | 32 | 1,057 | 1.08 | 0.67, 1.77 | 0.86 | 0.50, 1.46 |
| Yes | < 5^j^ | 61 | < 5^j^ | 202 | 0.70 | 0.17, 2.92 | 0.20 | 0.02, 1.80 |
| Missing | 15 |  | 16 |  |  |  |  |  |
| Trend^i^ |  |  |  |  | 0.99 | 0.66, 1.50 | 0.71 | 0.43, 1.16 |
| Exhaust from heaters or generators (e.g., kerosene heaters, tent heaters) |  |  |  |  |  |  |  |  |
| Not exposed | 66 | 1,648 | 94 | 3,840 | 1.00 | Referent | 1.00 | Referent |
| No | 26 | 564 | 33 | 1,163 | 1.40 | 0.86, 2.28 | 1.22 | 0.86, 1.74 |
| Yes | 5 | 77 | 10 | 448 | 0.76 | 0.30, 1.93 | 0.33 | 0.07, 1.55 |
| Missing | 7 |  | 8 |  |  |  |  |  |
| Trend^i^ |  |  |  |  | 1.05 | 0.75, 1.47 | 0.80 | 0.50, 1.29 |
| Exposure to diesel and/or other petrochemical fumes |  |  |  |  |  |  |  |  |
| Not exposed | 30 | 795 | 39 | 1,535 | 1.00 | Referent | 1.00 | Referent |
| No | 57 | 1,279 | 80 | 3,057 | 0.89 | 0.56, 1.41 | 1.09 | 0.59, 2.01 |
| Yes | 14 | 334 | 19 | 729 | 1.11 | 0.57, 2.15 | 1.17 | 0.50, 2.73 |
| Missing | 3 |  | 7 |  |  |  |  |  |
| Trend^i^ |  |  |  |  | 1.01 | 0.73, 1.41 | 1.06 | 0.71, 1.58 |
| Burning trash or burning feces/manure |  |  |  |  |  |  |  |  |
| Not exposed | 52 | 1,206 | 75 | 3,016 | 1.00 | Referent | 1.00 | Referent |
| No | 39 | 1,103 | 51 | 2,078 | 1.18 | 0.76, 1.82 | 1.03 | 0.70, 1.51 |
| Yes | 9 | 126 | 13 | 436 | 1.35 | 0.65, 2.83 | 0.73 | 0.24, 2.23 |
| Missing | 4 |  | 6 |  |  |  |  |  |
| Trend^i^ |  |  |  |  | 1.17 | 0.85, 1.60 | 0.94 | 0.65, 1.36 |
| Exposure to paint, solvents, or petrochemical substances |  |  |  |  |  |  |  |  |
| Not exposed | 67 | 1,635 | 89 | 3,416 | 1.00 | Referent | 1.00 | Referent |
| No | 24 | 629 | 40 | 1,886 | 0.75 | 0.46, 1.21 | 0.93 | 0.60, 1.45 |
| Yes | < 5^j^ | 96 | 5 | 155 | 1.06 | 0.38, 2.97 | 0.62 | 0.36, 1.08 |
| Missing | 9 |  | 11 |  |  |  |  |  |
| Trend^i^ |  |  |  |  | 0.85 | 0.57, 1.26 | 0.86 | 0.63, 1.18 |
|  |  |  |  |  |  |  |  |  |
|  |  |  |  |  |  |  |  |  |
| High-intensity radar waves (e.g., as radar operator, radio operator, aviation electrician's mate) |  |  |  |  |  |  |  |  |
| Not exposed | 68 | 1,671 | 95 | 3,807 | 1.00 | Referent | 1.00 | Referent |
| No | 24 | 562 | 29 | 944 | 1.30 | 0.79, 2.14 | 1.42 | 0.90, 2.22 |
| Yes | < 5^j^ | 15 | < 5^j^ | 92 | 1.50 | 0.19, 11.61 | 2.51 | 0.44, 14.32 |
| Missing | 11 |  | 19 |  |  |  |  |  |
| Trend^i^ |  |  |  |  | 1.29 | 0.81, 2.04 | 1.42 | 0.93, 2.17 |
| Food contaminated with smoke, oil, or other chemicals |  |  |  |  |  |  |  |  |
| Not exposed | 79 | 1,973 | 113 | 4,637 | 1.00 | Referent | 1.00 | Referent |
| No | 6 | 108 | 7 | 187 | 1.31 | 0.55, 3.15 | 0.97 | 0.49, 1.93 |
| Yes | < 5^j^ | 20 | 5 | 253 | 0.50 | 0.12, 2.09 | 0.11 | 0.01, 1.03 |
| Missing | 17 |  | 20 |  |  |  |  |  |
| Trend^i^ |  |  |  |  | 0.85 | 0.50, 1.46 | 0.59 | 0.30, 1.15 |
| Local food other than food provided by the Armed Forces |  |  |  |  |  |  |  |  |
| Not exposed | 47 | 1,094 | 64 | 2,462 | 1.00 | Referent | 1.00 | Referent |
| No | 49 | 1,113 | 67 | 2,537 | 1.06 | 0.70, 1.62 | 0.88 | 0.59, 1.33 |
| Yes | 5 | 167 | 9 | 467 | 0.74 | 0.28, 1.94 | 0.54 | 0.28, 1.04 |
| Missing | 3 |  | 5 |  |  |  |  |  |
| Trend^i^ |  |  |  |  | 0.97 | 0.70, 1.34 | 0.71 | 0.50, 1.01 |
| Bathing in or drinking of water contaminated with smoke, oil, dead animals or any chemicals |  |  |  |  |  |  |  |  |
| Not exposed | 80 | 1,965 | 107 | 4,079 | 1.00 | Referent | 1.00 | Referent |
| No | 8 | 107 | 11 | 348 | 1.12 | 0.52, 2.38 | 1.32 | 0.54, 3.22 |
| Yes | < 5^j^ | 15 | < 5^j^ | 250 | 0.26 | 0.04, 1.95 | 0.52 | 0.17, 1.64 |
| Missing | 15 |  | 23 |  |  |  |  |  |
| Trend^i^ |  |  |  |  | 0.76 | 0.43, 1.33 | 1.02 | 0.66, 1.57 |
| Heat cramps, heat exhaustion, heat stroke or other heat illness |  |  |  |  |  |  |  |  |
| Not exposed | 72 | 1,696 | 100 | 3,915 | 1.00 | Referent | 1.00 | Referent |
| No | 7 | 198 | 7 | 198 | 1.98 | 0.83, 4.71 | 1.53 | 0.89, 2.65 |
| Yes | 23 | 520 | 34 | 1,400 | 1.08 | 0.66, 1.76 | 0.86 | 0.51, 1.46 |
| Missing | 2 |  | 4 |  |  |  |  |  |
| Trend^i^ |  |  |  |  | 1.06 | 0.84, 1.35 | 0.94 | 0.73, 1.22 |
| Heard chemical alarms sounding |  |  |  |  |  |  |  |  |
| Not exposed | 99 | 2,389 | 137 | 5,422 | 1.00 | Referent | 1.00 | Referent |
| No | < 5^j^ | 11 | 5 | 226 | 0.61 | 0.15, 2.54 | 0.54 | 0.14, 2.18 |
| Yes | 0 | 0 | 0 | 0 | ^h^ | ^h^ | ^h^ | ^h^ |
| Missing | 3 |  | 3 |  |  |  |  |  |
| Trend^i^ |  |  |  |  | 0.61 | 0.15, 2.54 | 0.86 | 0.29, 2.58 |
| Explosion in the air or on the ground within one mile of you (e.g., artillery, rockets, mortars) |  |  |  |  |  |  |  |  |
| Not exposed | 23 | 543 | 36 | 1,575 | 1.00 | Referent | 1.00 | Referent |
| No | 71 | 1,703 | 96 | 3,674 | 1.16 | 0.71, 1.89 | 1.07 | 0.66, 1.74 |
| Yes | 6 | 136 | 8 | 301 | 2.65 | 0.98, 7.16 | 2.38 | 1.04, 5.46 |
| Missing | 4 |  | 5 |  |  |  |  |  |
| Trend^i^ |  |  |  |  | 1.35 | 0.87, 2.09 | 1.27 | 0.79, 2.02 |
| Mixing and application of riot control substances |  |  |  |  |  |  |  |  |
| Not exposed | 102 | 2,466 | 143 | 5,713 | 1.00 | Referent | 1.00 | Referent |
| No | < 5^j^ | 6 | < 5^j^ | 6 | 4.93 | 0.58, 41.68 | 3.68 | 1.59, 8.54 |
| Yes | 0 | 0 | 0 | 0 | ^h^ | ^h^ | ^h^ | ^h^ |
| Missing | 1 |  | 1 |  |  |  |  |  |
| Trend^i^ |  |  |  |  | 4.93 | 0.58, 41.68 | 3.67 | 1.62, 8.31 |
| Exposure to riot control substances in the field |  |  |  |  |  |  |  |  |
| Not exposed | 95 | 2,223 | 132 | 5,181 | 1.00 | Referent | 1.00 | Referent |
| No | 5 | 147 | 7 | 307 | 1.00 | 0.40, 2.53 | 0.65 | 0.36, 1.15 |
| Yes | < 5^j^ | 102 | < 5^j^ | 165 | 0.71 | 0.20, 2.52 | 0.45 | 0.16, 1.29 |
| Missing | 1 |  | 2 |  |  |  |  |  |
| Trend^i^ |  |  |  |  | 0.89 | 0.53, 1.49 | 0.70 | 0.40, 1.20 |
| Mixing and application of burning agents |  |  |  |  |  |  |  |  |
| Not exposed | 89 | 2,168 | 128 | 5,236 | 1.00 | Referent | 1.00 | Referent |
| No | 9 | 158 | 10 | 249 | 2.10 | 0.99, 4.47 | 1.83 | 1.22, 2.73 |
| Yes | < 5^j^ | 19 | < 5^j^ | 19 | 2.27 | 0.53, 9.84 | 1.43 | 0.89, 2.29 |
| Missing | 4 |  | 5 |  |  |  |  |  |
| Trend^i^ |  |  |  |  | 1.74 | 1.04, 2.93 | 1.51 | 1.11, 2.04 |
| Exposure to burning agents in the field |  |  |  |  |  |  |  |  |
| Not exposed | 78 | 1,917 | 109 | 4,396 | 1.00 | Referent | 1.00 | Referent |
| No | 18 | 420 | 22 | 730 | 1.09 | 0.63, 1.89 | 0.90 | 0.57, 1.42 |
| Yes | < 5^j^ | 61 | 6 | 275 | 0.66 | 0.20, 2.12 | 0.08 | 0.01, 0.67 |
| Missing | 5 |  | 8 |  |  |  |  |  |
| Trend^i^ |  |  |  |  | 0.94 | 0.63, 1.40 | 0.65 | 0.40, 1.07 |

Abbreviations: ALS, amyotrophic lateral sclerosis; ALSFRS-R, ALS Functional Rating Scale-Revised; CI, confidence interval; GENEVA, Genes and Environmental Exposures in Veterans with Amyotrophic Lateral Sclerosis; HR, hazard ratio; IP, inverse probability; PM, person-months; VA, Department of Veterans Affairs.

^a^ The GENEVA study questionnaire asked "Were you deployed to..." the Vietnam War (defined as the period from August 3, 1964, to May 7, 1975) [[1](#_ENREF_1)].

^b^ Fourteen ALS cases were excluded from this analysis because (1) they were missing data on diagnosis date (n = 7) or (2) they died before GENEVA enrollment (i.e., enrollment was completed by proxy after the case died; n = 7).

^c^ Adjusted for age (modeled with indicator variables corresponding to 5-year groups).

^d^ Weighted for confounding (conditional on age [modeled with indicator variables corresponding to 5-year groups]), not missing baseline ALSFRS-R score (conditional on most recent ALS diagnosis category, symptom onset site, diagnostic delay [months; modeled with the natural logarithm of a linear term], and time from diagnosis to enrollment in the Registry [months; modeled with a restricted, quadratic spline with knots at 7.72, 13.24, 23.06, and 44.19 months based on percentiles of the distribution in the Registry cases not missing baseline ALSFRS-R score]), and participating in GENEVA (conditional on race/ethnicity, being a current patient of a VA Medical Center, most recent ALS diagnosis category, symptom onset site, diagnostic delay [months; modeled with linear, quadratic, and cubic terms], time from diagnosis to enrollment in the Registry [months; modeled with a linear term], and baseline ALSFRS-R score [modeled with a restricted, quadratic spline with knots at 12, 34, and 44 based on percentiles of the distribution in GENEVA cases]). 95% CIs were calculated with robust variance estimates.

^e^ Person-months calculated for time on study (i.e., the difference between the GENEVA enrollment date and the death date or July 25, 2013).

^f^ HRs and 95% CIs correspond to time since diagnosis accounting for late entry into the risk set at the GENEVA enrollment date.

^g^ Repeated from Table C in S1 Supporting Information.

^h^ Unable to estimate HR and 95% CI.

^i^ Used ordinal scores (0, 1, 2). Reference category included for linear trend test.

^j^ Suppressed to preserve the confidentiality of study participants.

Table H. Selected military exposures and amyotrophic lateral sclerosis survival in GENEVA^a^, United States of America, 2005-2013, restricted to cases who enrolled in GENEVA within two years of diagnosis and without weighting for potential missing-covariate-data or selection bias.

|  | Incident  cases only  Adjusted^b, c^ | |
| --- | --- | --- |
| Exposure | HR^d^ | 95% CI^d^ |
| ***While you were in WWII, the Korean War, the Vietnam War, or the Gulf War^e^: did you have direct contact with/were you exposed to*** |  |  |
| Exposure to paint, solvents, or petrochemical substances | 0.68 | 0.40, 1.15 |
| Local food other than food provided by the Armed Forces | 0.72 | 0.46, 1.12 |
| ***While you were in WWII, the Korean War, or the Vietnam War^e^: did you have direct contact with/were you exposed to*** |  |  |
| Mixing and application of burning agents | 2.99 | 1.27, 7.03 |
| Exposure to burning agents in the field | 1.20 | 0.67, 2.16 |
| ***While you were in the Vietnam War^e^: did you have direct contact with/were you exposed to*** |  |  |
| Mixing and application of Agent Orange | 0.60 | 0.14, 2.48 |
| Exposure to Agent Orange in the field | 0.76 | 0.42, 1.36 |

Abbreviation: ALS, amyotrophic lateral sclerosis; CI, confidence interval; GENEVA, Genes and Environmental Exposures in Veterans with Amyotrophic Lateral Sclerosis study; Gulf, 1990-1991 Persian Gulf; HR, hazard ratio; WWII, World War II.

^a^ Fourteen ALS cases were excluded from this analysis because (1) they were missing data on diagnosis date (n = 7) or (2) they died before GENEVA enrollment (i.e., enrollment was completed by proxy after the case died; n = 7).

^b^ Cases who did not experience direct contact with each specific exposure were the reference.

^c^ Adjusted for age (modeled with indicator variables corresponding to 5-year groups), war/operation of longest deployment, and the pairwise interaction between war/operation of longest deployment and the natural logarithm of time since diagnosis.

^d^ HRs and 95% CIs correspond to time since diagnosis accounting for late entry into the risk set at the GENEVA enrollment date.

^e^ The GENEVA study questionnaire asked "Were you deployed to..." the following wars where each war was asked about with a separate question: World War II (defined as the period from December 7, 1941, to December 31, 1946), the Korean War (defined as the period from June 27, 1950, to January 31, 1955), the Vietnam War (defined as the period from August 3, 1964, to May 7, 1975), and the Persian Gulf War (defined as the period from August 2, 1990, to December 31, 1991) [[1](#_ENREF_1)].

Table I. Military service and amyotrophic lateral sclerosis survival in GENEVA^a^, United States of America, 2005-2013, without weighting for potential missing-covariate-data or selection bias.

|  | Adjusted^b^ | |
| --- | --- | --- |
| Variable | HR^c^ | 95% CI^c^ |
| Military branch of longest service |  |  |
| Air Force (including Army Air Force) | 1.11 | 0.86, 1.42 |
| Army | 1.00 | Referent |
| Marines (including Merchant Marines) | 1.00 | 0.69, 1.43 |
| Navy | 0.93 | 0.72, 1.19 |
| Other^d^ | 0.94 | 0.64, 1.37 |
| Number of military branches of service |  |  |
| 1 (Median = 1) | 1.00 | Referent |
| > 1 (2) | 0.97 | 0.68, 1.38 |
| Officer or Warrant Officer |  |  |
| No | 1.00 | Referent |
| Yes | 1.02 | 0.79, 1.32 |
| Years of military service |  |  |
| ≤ 1 (0.50) | 1.74 | 0.98, 3.07 |
| > 1-5 (2.92) | 1.00 | Referent |
| > 5-10 (7.92) | 0.92 | 0.65, 1.28 |
| > 10-15 (11.99) | 0.92 | 0.52, 1.64 |
| > 15-20 (18.92) | 1.56 | 0.97, 2.52 |
| > 20 (24.31) | 0.83 | 0.62, 1.13 |
| Trend (IQR = 6.00)^e, f^ | 0.97 | 0.89, 1.04 |
| End of most recent period of military service (month/year)^g^ |  |  |
| ≤ 12/1946 (03/1946) | 1.41 | 0.77, 2.58 |
| 01/1947-06/1950 (02/1948) | 1.25 | 0.56, 2.78 |
| 07/1950-01/1955 (06/1953) | 1.04 | 0.64, 1.67 |
| 02/1955-02/1961 (12/1957) | 1.03 | 0.71, 1.48 |
| 03/1961-07/1964 (06/1963) | 0.87 | 0.57, 1.33 |
| 08/1964-04/1975 (07/1969) | 1.00 | Referent |
| 05/1975-08/1980 (05/1978) | 1.12 | 0.72, 1.74 |
| 09/1980-07/1990 (03/1986) | 0.98 | 0.66, 1.45 |
| 08/1990-08/2001 (06/1994) | 0.97 | 0.67, 1.40 |
| > 08/2001 (10/2003) | 0.89 | 0.45, 1.76 |
| Trend (20 years, 11.99 months)^e, f^ | 0.94 | 0.76, 1.17 |

Abbreviation: ALS, amyotrophic lateral sclerosis; CI, confidence interval; GENEVA, Genes and Environmental Exposures in Veterans with Amyotrophic Lateral Sclerosis study; HR, hazard ratio; IQR, interquartile range.

^a^ Fourteen ALS cases were excluded from this analysis because (1) they were missing data on diagnosis date (n = 7) or (2) they died before GENEVA enrollment (i.e., enrollment was completed by proxy after the case died; n = 7).

^b^ Adjusted for age (modeled with indicator variables corresponding to 5-year groups).

^c^ HRs and 95% CIs correspond to time since diagnosis accounting for late entry into the risk set at the GENEVA enrollment date.

^d^ Includes Coast Guard, Activated National Guard, Activated Reserves, Inactivated National Guard, Inactivated Reserves, Department of Defense, National Oceanic and Atmospheric Administration, and Public Health Service.

^e^ Used within-category medians that were calculated using all cases.

^f^ Scaled the HR to an IQR-unit increase in the exposure variable. IQRs were calculated using all cases.

^g^ Category boundaries aligned with the occurrence of the major wars (e.g., the Vietnam War occurred between August 1964 and May 1975) and followed Allen et al. [[10](#_ENREF_10)], Beard et al. [[2](#_ENREF_2)], and Schmidt et al. [[3](#_ENREF_3)].

Table J. Military deployments or danger pay and amyotrophic lateral sclerosis survival in GENEVA^a^, United States of America, 2005-2013, without weighting for potential missing-covariate-data or selection bias.

|  | Adjusted^b^ | |
| --- | --- | --- |
| Variable | HR^c^ | 95% CI^c^ |
| ***Deployments*** |  |  |
| Ever deployed to any war/operation^d^ |  |  |
| No | 1.00 | Referent |
| Yes | 0.99 | 0.81, 1.21 |
| War/operation of longest deployment^d^ |  |  |
| Not deployed | 1.00 | Referent |
| World War II | 0.81 | 0.47, 1.41 |
| Korean War | 1.32 | 0.87, 2.00 |
| Vietnam War | 0.98 | 0.76, 1.26 |
| Gulf War | 0.48 | 0.18, 1.30 |
| Other^e^ | 1.13 | 0.65, 1.99 |
| Ever deployed to any other country |  |  |
| No | 1.00 | Referent |
| Yes | 0.96 | 0.79, 1.16 |
| Total time (years) of all periods of deployment to any war/operation^d^ |  |  |
| Not deployed (Median = 0.00) | 1.00 | Referent |
| ≤ 1 (0.67) | 1.05 | 0.82, 1.35 |
| > 1-2 (1.25) | 0.82 | 0.59, 1.14 |
| > 2-4 (2.37) | 1.46 | 0.98, 2.19 |
| > 4 (6.00) | 0.71 | 0.34, 1.45 |
| Trend^f^ | 0.98 | 0.89, 1.08 |
| End of most recent period of deployment to any war/operation (month/year)^d, g^ |  |  |
| Not deployed | 1.00 | Referent |
| ≤ 06/1950 (01/1946) | 0.80 | 0.46, 1.41 |
| 07/1950-01/1955 (01/1953) | 1.25 | 0.79, 1.98 |
| 02/1955-02/1961 (02/1956) | 1.86 | 0.85, 4.08 |
| 03/1961-04/1975 (02/1969) | 1.00 | 0.78, 1.29 |
| 05/1975-07/1990 (08/1980) | 1.02 | 0.45, 2.32 |
| > 07/1990 (07/1992) | 0.72 | 0.39, 1.33 |
| Trend (IQR=14 years, 11.98 months)^f, h^ | 0.86 | 0.60, 1.24 |
| ***Danger pay, hardship duty or combat zone tax exclusion benefits for deployment*** |  |  |
| Ever received imminent danger pay, hardship duty or combat zone tax exclusion benefits for deployment |  |  |
| No | 1.00 | Referent |
| Yes | 0.94 | 0.74, 1.19 |
| Total time (years) of all periods of deployment to any countries or sea region(s) ever received imminent  danger pay, hardship duty or combat zone tax exclusion benefits for deployment |  |  |
| Never received imminent danger pay, hardship duty or combat zone tax exclusion benefits for deployment  (0.00) | 1.00 | Referent |
| ≤ 1 (0.83) | 0.90 | 0.66, 1.24 |
| > 1-2 (1.13) | 1.03 | 0.66, 1.62 |
| > 2 (2.50) | 1.17 | 0.57, 2.39 |
| Trend^f^ | 1.00 | 0.81, 1.25 |

Abbreviation: ALS, amyotrophic lateral sclerosis; CI, confidence interval; GENEVA, Genes and Environmental Exposures in Veterans with Amyotrophic Lateral Sclerosis study; Gulf, 1990-1991 Persian Gulf; HR, hazard ratio; IQR, interquartile range.

^a^ Fourteen ALS cases were excluded from this analysis because (1) they were missing data on diagnosis date (n = 7) or (2) they died before GENEVA enrollment (i.e., enrollment was completed by proxy after the case died; n = 7).

^b^ Adjusted for age (modeled with indicator variables corresponding to 5-year groups).

^c^ HRs and 95% CIs correspond to time since diagnosis accounting for late entry into the risk set at the GENEVA enrollment date.

^d^ The GENEVA study questionnaire asked "Were you deployed to..." the following wars where each war was asked about with a separate question: World War II (defined as the period from December 7, 1941, to December 31, 1946), the Korean War (defined as the period from June 27, 1950, to January 31, 1955), the Vietnam War (defined as the period from August 3, 1964, to May 7, 1975), and the Persian Gulf War (defined as the period from August 2, 1990, to December 31, 1991) [[1](#_ENREF_1)]. The questionnaire also asked "Ever deployed..." to the following countries where each country was asked about with a separate question: Grenada, Lebanon, Panama, Somalia, Bosnia, Kosovo, Rwanda, Afghanistan, and Iraq/Persian Gulf region (Gulf War II) [[1](#_ENREF_1)].

^e^ Includes Grenada, Lebanon, Panama, Somalia, Bosnia, Kosovo, Rwanda, Afghanistan, and Iraq/Persian Gulf region (Gulf War II).

^f^ Used within-category medians that were calculated using all cases.

^g^ Category boundaries aligned with the occurrence of the major wars (e.g., the Vietnam War occurred between August 1964 and May 1975) and followed Allen et al. [[10](#_ENREF_10)], Beard et al. [[2](#_ENREF_2)], and Schmidt et al. [[3](#_ENREF_3)].

^h^ Scaled the HR to an IQR-unit increase in the exposure variable. IQRs were calculated using all cases except those in the reference category. Reference category excluded for linear trend test.

Table K. Military exposures and amyotrophic lateral sclerosis survival in GENEVA^a^, United States of America, 2005-2013, without weighting for potential missing-covariate-data or selection bias.

|  | Adjusted^c, d^ | | Adjusted^d, e^ | |
| --- | --- | --- | --- | --- |
| Exposure^b^ | HR^f^ | 95% CI^f^ | HR^f^ | 95% CI^f^ |
| Ever received the anthrax vaccine prior to reference date | 1.32 | 0.86, 2.03 | 1.47 | 0.92, 2.35 |
| Ever received the smallpox vaccine | 1.01 | 0.74, 1.37 | 1.02 | 0.75, 1.39 |
| Prior to reference date, ever involved in testing, transporting or spraying herbicides for military purposes | 0.78 | 0.41, 1.48 | 0.79 | 0.41, 1.51 |
| Prior to reference date, ever been treated with nasopharyngeal (NP) radium during military service | 1.43 | 0.44, 4.64 | 1.49 | 0.45, 4.91 |
| Ever taken pyridostigmine bromide, or little white pills in foil packs, sometimes called NAPPs, which are used to protect against nerve agents | 0.90 | 0.47, 1.73 | 0.97 | 0.50, 1.88 |
| Prior to reference date, ever visited or resided in the island of Guam, the islands of New Guinea, or the Kii Peninsula of Japan (including any time spent there in the military) | 1.00 | 0.76, 1.30 | 1.02 | 0.78, 1.35 |
| ***While you were in WWII, the Korean War, the Vietnam War, or the Gulf War^g^: did you have direct contact with/were you exposed to*** |  |  |  |  |
| Ionizing radiation from nuclear weapon testing or occupation of Hiroshima/Nagasaki | 1.78 | 0.55, 5.77 | 2.18 | 0.66, 7.18 |
| Use of personal pesticides, like creams, sprays or flea collars | 1.25 | 0.87, 1.78 | 1.29 | 0.89, 1.86 |
| Use of pesticides on your clothing or bedding | 1.43 | 0.94, 2.16 | 1.39 | 0.91, 2.11 |
| Exhaust from heaters or generators (e.g., kerosene heaters, tent heaters) | 0.94 | 0.66, 1.34 | 0.96 | 0.67, 1.37 |
| Exposure to diesel and/or other petrochemical fumes | 0.99 | 0.70, 1.41 | 1.03 | 0.72, 1.47 |
| Burning trash or burning feces/manure | 1.11 | 0.76, 1.61 | 1.08 | 0.74, 1.58 |
| Exposure to paint, solvents, or petrochemical substances | 0.74 | 0.52, 1.06 | 0.77 | 0.53, 1.10 |
| High-intensity radar waves (e.g., as radar operator, radio operator, aviation electrician's  mate) | 1.17 | 0.79, 1.75 | 1.27 | 0.84, 1.90 |
| Food contaminated with smoke, oil, or other chemicals | 1.01 | 0.53, 1.92 | 0.95 | 0.50, 1.81 |
| Local food other than food provided by the Armed Forces | 0.84 | 0.61, 1.17 | 0.84 | 0.60, 1.17 |
| Bathing in or drinking of water contaminated with smoke, oil, dead animals or any  chemicals | 0.89 | 0.48, 1.65 | 0.88 | 0.47, 1.63 |
| Heat cramps, heat exhaustion, heat stroke or other heat Illness | 1.10 | 0.74, 1.65 | 1.11 | 0.74, 1.66 |
| Heard chemical alarms sounding | 0.60 | 0.28, 1.30 | 0.69 | 0.30, 1.57 |
| Explosion in the air or on the ground within one mile of you (e.g., artillery, rockets,  mortars) | 1.30 | 0.92, 1.86 | 1.26 | 0.88, 1.80 |
| Have you suffered a combat-related injury that required medical attention during your  deployment? | 1.03 | 0.71, 1.51 | 0.98 | 0.67, 1.45 |
| ***While you were in WWII, the Korean War, or the Vietnam War^g^: did you have direct contact with/were you exposed to*** |  |  |  |  |
| Mixing and application of herbicides | 2.41 | 0.85, 6.87 | 2.03 | 0.69, 6.02 |
| Exposure to herbicides in the field | 1.34 | 0.68, 2.62 | 1.29 | 0.65, 2.56 |
| Mixing and application of riot control substances | 4.54 | 1.01, 20.42 | 4.83 | 1.08, 21.61 |
| Exposure to riot control substances in the field | 0.96 | 0.48, 1.92 | 0.99 | 0.49, 2.00 |
| Mixing and application of burning agents | 2.22 | 1.19, 4.12 | 2.41 | 1.29, 4.49 |
| Exposure to burning agents in the field | 0.84 | 0.52, 1.35 | 0.87 | 0.54, 1.40 |
| ***While you were in the Korean War, the Vietnam War, or the Gulf War^g^: did you have direct contact with/were you exposed to*** |  |  |  |  |
| Microwave radiation | 1.15 | 0.59, 2.25 | 1.14 | 0.57, 2.24 |
| ***While you were in the Vietnam War^g^: did you have direct contact with/were you exposed to*** |  |  |  |  |
| Mixing and application of Agent Orange | 1.11 | 0.47, 2.61 |  |  |
| Exposure to Agent Orange in the field | 0.73 | 0.46, 1.17 |  |  |
| ***While you were in the Gulf War^g^: did you have direct contact with/were you exposed to*** |  |  |  |  |
| Use of depleted uranium (DU) for munitions or armor | 0.35 | 0.04, 3.39 |  |  |
| CARC (Chemical Agent Resistant Compound) paint | 1.56 | 0.19, 13.01 |  |  |
| Scud missile explosion in the air or on the ground within one mile of you | 12.50 | 0.52, 298.52 |  |  |
| Smoke from oil well fires | 0.12 | 0.01, 1.41 |  |  |
| Exposure to nerve gas (e.g., during munitions destruction) | ^h^ | ^h^ |  |  |
| High levels of dust/sand | 0.17 | 0.01, 2.33 |  |  |
| Ground level fumigation | ^h^ | ^h^ |  |  |
| In any conflicts deployed to, any other exposure or experience not asked about which you consider harmful or extremely stressful | 1.06 | 0.77, 1.46 | 1.05 | 0.75, 1.48 |

Abbreviation: ALS, amyotrophic lateral sclerosis; CARC, Chemical Agent Resistant Compound; CI, confidence interval; DU, depleted uranium; GENEVA, Genes and Environmental Exposures in Veterans with Amyotrophic Lateral Sclerosis study; Gulf, 1990-1991 Persian Gulf; HR, hazard ratio; NP, nasopharyngeal; WWII, World War II.

^a^ Fourteen ALS cases were excluded from this analysis because (1) they were missing data on diagnosis date (n = 7) or (2) they died before GENEVA enrollment (i.e., enrollment was completed by proxy after the case died; n = 7).

^b^ Information for specific exposures was missing for 0-49% of cases.

^c^ Adjusted for age (modeled with indicator variables corresponding to 5-year groups or, for exposures queried only in reference to deployment to the Gulf War, modeled with a linear term that was centered at 60—the median among all cases).

^d^ Cases who did not experience direct contact with each specific exposure were the reference.

^e^ Adjusted for age (modeled with indicator variables corresponding to 5-year groups), war/operation of longest deployment, and the pairwise interaction between war/operation of longest deployment and the natural logarithm of time since diagnosis.

^f^ HRs and 95% CIs correspond to time since diagnosis accounting for late entry into the risk set at the GENEVA enrollment date.

^g^ The GENEVA study questionnaire asked "Were you deployed to..." the following wars where each war was asked about with a separate question: World War II (defined as the period from December 7, 1941, to December 31, 1946), the Korean War (defined as the period from June 27, 1950, to January 31, 1955), the Vietnam War (defined as the period from August 3, 1964, to May 7, 1975), and the Persian Gulf War (defined as the period from August 2, 1990, to December 31, 1991) [[1](#_ENREF_1)].

^h^ Unable to estimate HR and 95% CI.

References

1. Beard JD. Military Service, Deployments, and Exposures in Relation to Amyotrophic Lateral Sclerosis Etiology and Survival. ETD Dissertation, University of North Carolina at Chapel Hill. 2015.

2. Beard JD, Engel LS, Richardson DB, Gammon MD, Baird C, Umbach DM, et al. Military service, deployments, and exposures in relation to amyotrophic lateral sclerosis etiology. Environ Int. 2016;91: 104-115.

3. Schmidt S, Allen KD, Loiacono VT, Norman B, Stanwyck CL, Nord KM, et al. Genes and Environmental Exposures in Veterans with Amyotrophic Lateral Sclerosis: the GENEVA study. Rationale, study design and demographic characteristics. Neuroepidemiology. 2008;30: 191-204.

4. Cedarbaum JM, Stambler N, Malta E, Fuller C, Hilt D, Thurmond B, et al. The ALSFRS-R: a revised ALS functional rating scale that incorporates assessments of respiratory function. J Neurol Sci. 1999;169: 13-21.

5. Glymour MM, Greenland S. Causal diagrams. In: Rothman KJ, Greenland S, Lash TL, editors. Modern Epidemiology. 3rd ed. Philadelphia, PA: Lippincott, Williams, & Wilkins; 2008. pp. 183-209.

6. Greenland S, Pearl J, Robins JM. Causal diagrams for epidemiologic research. Epidemiology. 1999;10: 37-48.

7. Cole SR, Hernan MA. Constructing inverse probability weights for marginal structural models. Am J Epidemiol. 2008;168: 656-664.

8. Hernan MA, Hernandez-Diaz S, Robins JM. A structural approach to selection bias. Epidemiology. 2004;15: 615-625.

9. Robins JM, Hernan MA, Brumback B. Marginal structural models and causal inference in epidemiology. Epidemiology. 2000;11: 550-560.

10. Allen KD, Kasarskis EJ, Bedlack RS, Rozear MP, Morgenlander JC, Sabet A, et al. The National Registry of Veterans with Amyotrophic Lateral Sclerosis. Neuroepidemiology. 2008;30: 180-190.

11. Howe CJ, Cole SR, Westreich DJ, Greenland S, Napravnik S, Eron JJ, Jr. Splines for trend analysis and continuous confounder control. Epidemiology. 2011;22: 874-875.

12. Akaike H. A new look at the statistical model identification. IEEE Trans Automat Contr. 1974;19: 716-723.

13. Greenland S. Invited commentary: variable selection versus shrinkage in the control of multiple confounders. Am J Epidemiol. 2008;167: 523-529; discussion 530-531.

14. Winer BJ. Statistics and data analysis: trading bias for reduced mean squared error. Annu Rev Psychol. 1978;29: 647-681.
